# An Investigation of the Anti-Depressive Properties of Phenylpropanoids and Flavonoids in *Hemerocallis citrina* Baroni

**DOI:** 10.3390/molecules27185809

**Published:** 2022-09-08

**Authors:** Tiancheng Ma, Yu Sun, Lida Wang, Jinyu Wang, Bo Wu, Tingxu Yan, Ying Jia

**Affiliations:** 1 School of Traditional Chinese Materia Medica, Shenyang Pharmaceutical University, Wenhua Road 103, Shenyang 110016, China; 2 Research Institute of Medicine and Pharmacy, Qiqihar Medical University, Bukui North Street 333, Qiqihar 161006, China; 3 School of Functional Food and Wine, Shenyang Pharmaceutical University, Wenhua Road 103, Shenyang 110016, China

**Keywords:** phenylpropanoids, flavonoids, *Hemerocallis citrina* Baroni, antidepressant activity, anti-neuroinflammatory activity

## Abstract

The World Health Organization predicts that over the next several years, depression will become the most important mental health issue globally. Growing evidence shows that the flower buds of *Hemerocallis citrina* Baroni (*H. citrina*) possess antidepressant properties. In the search for new anti-depression drugs, a total of 15 phenylpropanoids and 22 flavonoids were isolated and identified based on spectral data (1D and 2D NMR, HR-ESI-MS, UV) from *H. citrina*. Among them, compound **8** was a novel compound, while compounds **1**–**4**, **6**, **9**, **10**, **15**, **17**, **24**–**26**, **28**, and **37** were isolated for the first time from *Hemerocallis* genus. To study the antidepressant activity of phenylpropanoids and flavonoids fractions from *H. citrina*, macroporous resin was used to enrich them under the guidance of UV characteristics. UHPLC-MS/MS was applied to identify the constituents of the enriched fractions. According to behavioral tests and biochemical analyses, it showed that phenylpropanoid and flavonoid fractions from *H. citrina* can improve the depressive-like mental state of chronic unpredictable mild stress (CUMS) rats. This might be accomplished by controlling the amounts of the inflammatory proteins IL-6, IL-1β, and TNF-α in the hippocampus as well as corticosterone in the serum. Thus, the monomer compounds were tested for their anti-neuroinflammatory activity and their structure–activity relationship was discussed in further detail.

## 1. Introduction

*Hemerocallis citrina* Baroni (*H. citrina*) belongs to Liliaceae family [1], which is distributed widely from Europe to Asia. *H. citrina* is utilized as food and medicine because it has a pleasant flavor and bioactive secondary metabolites [2]. The flower buds are harvested before the plant blooms and dried, and they are regarded as a useful meal. The benefits, such as improving sleep and curing depression, were initially recorded in the book *Bencao Gangmu Shiyi*. Research conducted in the 21st century indicates that *H. citrina* possesses a number of beneficial effects, including antidepressant properties [3], anti-inflammatory properties [4], alleviation of insomnia [4], improvement of hepatitis [5], and anticancer properties [6]. According to phytochemical studies, several classes of biologically active components are present, including alkaloids [7], flavones [8], terpenes [9], steroidal saponins [10], and phenolic glycosides [11]. The search for antidepressant active ingredients in natural medicines and foods has undergone a new strategy. There is evidence that the total phenols extract of *H. citrina* has antidepressant properties [12], but the exact active ingredients remain unknown. As a result, we conducted a systematic extraction and separation of *H. citrina*, and the total phenols of *H. citrina* were mainly classified as phenylpropanoids and flavonoids. 

Many individuals are afflicted with depressive disorder (DD), a prevalent mental illness [13]. DD has recently emerged as one of the major contributors to disability, and by 2030 it will rank as the second-most significant contributor to illness [14,15]. There is strong evidence that neuroinflammation plays an important role in the pathophysiology of DD. Patients with major depressive disorder have been found to have increased proinflammatory factors such as interleukin (IL)-6, IL-1β, and tumor necrosis factor (TNF)-α [16]. These proinflammatory factors disrupt neurotransmitter synthesis and signal transduction leading to central nervous system disorders that promote depression-like behaviors [17,18]. Inhibition of neuroinflammation is considered to be one of the most crucial aspects of treating DD. Inflammation of the central nervous system (CNS) causes neuroinflammation as a response. Microglial cells, which account for 10–12% of brain cell populations, are the primary immune cells in the CNS [19]. Microglia cells are particularly abundant in the hippocampus [20]. Additionally, it may remove and neutralize certain toxic compounds while strengthening tissues [21]. Microglia cells were activated in response to concrete damage, and pro-inflammatory cytokines (IL-6, IL-1β, and TNF-α) and pro-inflammatory enzymes (inducible nitric oxide synthase) were also upregulated [22,23]. The chronic unpredictable mild stress (CUMS) model is recognized as a good model for simulating stress-induced depression, and has been widely used in preclinical studies with predictive and etiological validity [24]. Thus, in this study, we use the CUMS model to investigate the antidepressant-like activity.

In this work, based on UV characteristics of different types of total phenols, the active fractions of *H. citrina* were identified and enriched by macroporous resin. The antidepressant effects of phenylpropanoids and flavonoids fractions were assessed. It identified that they may treat DD by inhibiting inflammation in the nervous system. Afterwards, we examined the monomeric compounds of *H. citrina* on anti-inflammation and revealed the structure–activity relationship. 

## 2. Results and Discussion

### 2.1. Structure Elucidation of Novel Compound 

**Figure 1:**
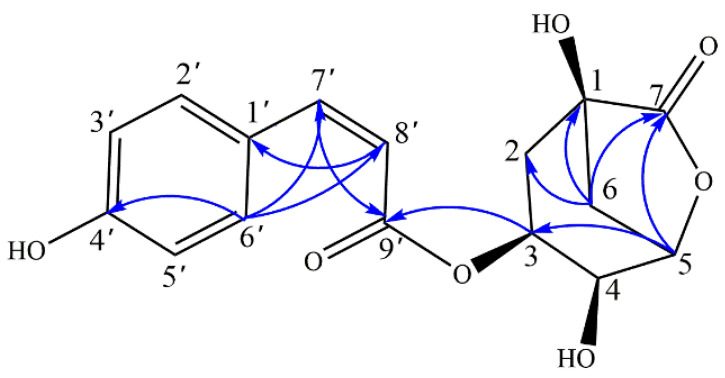
Key HMBC correlations of compound **8**.

Compound **8** was obtained as white powder. The molecular formula of **8** was deduced to be C_16_H_16_O_7_ by HR-ESI-MS analysis, displaying an excimer ion at *m/z* 319.0824 (calcd [M-H]^−^, *m/z* 319.0818). The UV spectrum of **8** exhibited absorption maxima at 310 nm. The ^1^H-NMR spectrum (Table 1), exhibited signals of four aromatic protons of a 1,4-disubstituted benzene ring at *δ*_H_ 6.76 (2H, br d, *J* = 8.7 Hz) and 7.68 (2H, br d, *J* = 8.4 Hz). A pair of double bonds between *δ*_H_ 6.90 (1H, d, *J* = 12.8 Hz) and 5.82 (1H, d, *J* = 12.8 Hz) were assigned to the 7′ and 8′ protons of the olefin portion of the coumaroyl, and the coupling constants indicate that they are cis configuration. Furthermore, the spectrum allowed the identification of three oxygenated methine groups at *δ*_H_ 4.88 (1H, m), 4.28 (1H, t, *J* = 4.6 Hz) and 4.72 (1H, t, *J* = 5.4 Hz) and two methylene groups at 2.03 (1H, t, *J* = 11.8 Hz), 2.12 (1H, ddd, *J* = 2.7, 6.9, 11.5 Hz), 2.29 (1H, ddd, *J* = 2.8, 6.0, 11.5 Hz), and 2.53 (1H, d, *J* = 11.8 Hz). A combination of ^13^C-NMR and HSQC experiments indicated the presence of 16 carbon signals for compound **8**, confirming the presence of the above-mentioned groups and revealing other moieties, including two acetyl groups (*δ*_C_ 167.0 and 179.1). In the HMBC spectrum of **8**, the correlations between H-3 and C-9′ showed that the coumaroyl was linked to the hydroxyl of C-3. The cross peak between H-5 and C-7 showed that the hydroxyl of C-5 dehydrogenated with the carboxyl of C-7 to form a lactone ring. According to the MS data, it had the same molecular formula as compound **7**, indicating that the two are isomers. Careful analysis of spectrum data indicated that they shared similar structure skeleton with the exception of the *cis*-configuration of coumaroyl based on the chemical shift and coupling constants. Thus, the structure of compound **8** was determined as 3-O-(*Z*)-*p*-coumaroylquinide. The key HMBC correlations were shown in Figure 1.

### 2.2. Enrichment of Phenylpropanoids and Flavonoids

**Figure 2:**
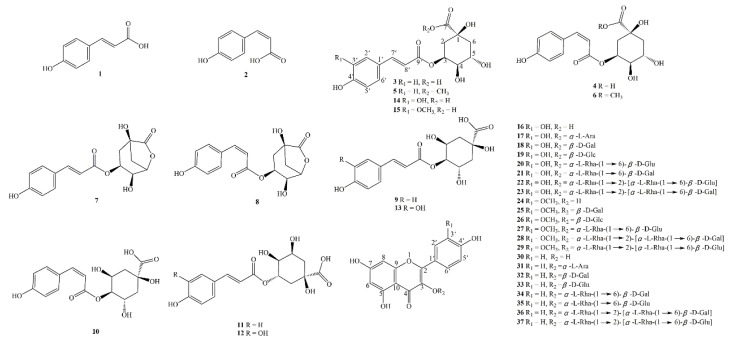
Structures of the isolated compounds.

Literature [12] reported that the total phenols extract of *H. citrina* had antidepressant property. The results obtained from UHPLC-MS/MS showed that the 20%, 30%, and 50% ethanol eluted fraction were rich in phenols. In order to find out the active ingredients from *H. citrina*, a systematic separation of was carried out for these fractions. At last, 15 phenylpropanoids and 22 flavonoids were isolated and identified from *H. citrina* (Figure 2). Among them, compound **8** was a novel compound, compounds **1**–**4**, **6**, **9**, **10**, **15**, **17**, **24**–**26**, **28**, and **37** were isolated from *Hemerocallis* genus for the first time. These findings greatly enrich the compound diversity of *H. citrina.* Their structures were identified by the comparison of their spectroscopic data with literature values and were assigned as (*E*)-*p*-coumaric acid (**1**) [25], *(Z)*-*p*-coumaric acid (**2**) [26], 3-O-(*E*)-*p*-coumaroylquinic acid (**3**) [27], 3-O-(*Z*)-*p*-coumaroylquinic acid (**4**) [28], 3-O-(*E*)-*p*-coumaroylquinic acid methyl ester (**5**) [29], 3-O-(*Z*)-*p*-coumaroylquinic acid methyl ester (**6**) [30], 3-O-(*E*)-*p*-coumaroylquinide (**7**) [31], 4-O-(*E*)-*p*-coumaroylquinic acid (**9**) [32], 4-O-(*Z*)-*p*-coumaroylquinic acid (**10**), 5-O-(*E*)-*p*-coumaroylquinic acid (**11**) [33], neochlorogenic acid (**12**) [34], crypto-chlorogenic acid (**13**) [35], chlorogenic acid (**14**) [36], 3-O-(*E*)-feruloylquinic acid (**15**) [37], quercetin (**16**) [38], quercetin-3-O-*α*-L-arabinopyranoside (**17**) [39], quercetin-3-O-*β*-D-galactpyranoside (**18**) [40], quercetin-3-O-*β*-D-glucopyranoside (**19**) [41], quercetin-3-O-rutinoside (**20**) [41], quercetin-3-O-*α*-L-rhamnopyranosyl-(1→6)-*β*-D-galactpyranoside (**21**) [42], quercetin-3-O-*α*-L-rhamnopyranosyl-(1→2)-[*α*-L-rhamnopyranosyl-(1→6)-*β*-D-glucopyranoside] (**22**) [43], quercetin-3-O-*α*-L-rhamnopyranosyl-(1→2)-[*α*-L-rhamnopyranosyl-(1→6)-*β*-D-galactpyranoside] (**23**) [44], isorhamnetin (**24**) [45], isorhamnetin-3-O-*β*-D-galactopyranoside (**25**) [46], isorhamnetin-3-O-*β*-D-glucopyranoside (**26**) [47,48], isorhamnetin-3-O-[*α*-L-rhamnopyranosyl-(1→6)-β-D-glucopyranoside (**27**) [47], isorhamnetin-3-O-*α*-L-rhamnopyranosyl-(1→2)-[*α*-L-rhamnopyranosyl-(1→6)-*β*-D-galactopyranoside] (**28**) [42], isorhamnetin-3-O-*α*-L-rhamnopyranosyl-(1→2)-[*α*-L-rhamnopyranosyl-(1→6)-*β*-D-glucopyranoside] (**29**) [42], kaempferol (**30**) [49], kaempferol-3-O-*α*-L-arabinoside (**31**) [50], kaempferol-3-O-*β*-D-galactopyranoside (**32**) [50], kaempferol-3-O-*β*-D-glucopyranoside (**33**) [50], kaempferol-3-O-*α*-L-rhamnopyranosyl-(1→6)-*β*-D-galactpyranoside (**34**) [51], kaempferol-3-O-*α*-L-rhamnopyranosyl-(1→6)-*β*-D-glucopyranoside (**35**) [52], kaempferol-3-O-[*α*-L-rhamnopyranosyl-(1→6)]-[*α*-L-rhamnopyranosyl-(1→2)]-*β*-D-galactopyranoside (**36**) [42], kaempferol-3-O-[*α*-L-rhamnopyranosyl-(1→6)]-[*α*-L-rhamnopyranosyl-(1→2)]-*β*-D-glucopyranoside (**37**) [53]. As far as we know, the nuclear magnetic data of compound **10** was reported for the first time. The spectral data of these compounds were shown in supporting information.

Phenylpropanoids and flavonoids were the main phenols of *H. citrina*. In this study, five pairs of phenylpropanoid isomers were isolated. It was interesting that it showed such a high content of phenylpropanoids with *cis*-configuration in *H. citrina*. The flavonoids found in this study were all 3-O-flavonoids. The parent nucleuses of them were composed of quercetin, isorhamnetin, and kaempferol.

**Figure 3:**
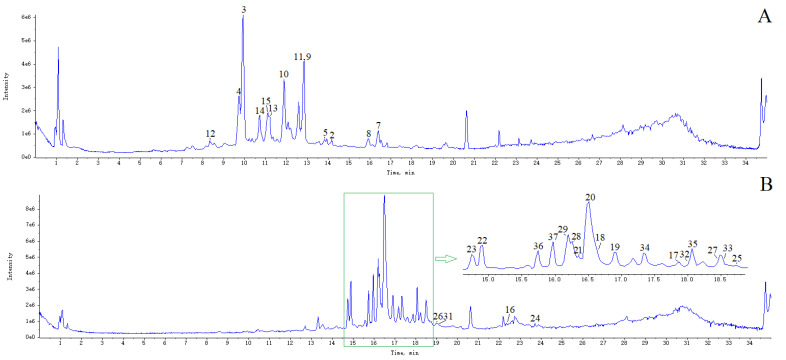
TIC spectra of HFPE (**A**) and HFFE (**B**).

To understand the role of phenylpropanoids and flavonoids of *H. citrina*, on the effect of anti-depression, the target compounds need to be enriched. A total of 70 ethanol elution fractions and 37 monomeric compounds were injected into HPLC system. The UV spectrum of each peak was studied. It showed that different kind of Different compounds exhibited different UV spectrum maximum absorption. (*E*)-*p*-coumaroylquinic acid and (*Z*)-*p*-coumaroylquinic acid exhibited absorption maxima at 310 and 306 nm, respectively. The UV spectrum maximum absorption wavelength of caffeoylquinic acid were around 218 and 326 nm. The UV spectrum maximum absorption wavelength of feruloylquinic acid were around 236 and 324 nm. The UV spectra of the compounds were shown in the supporting information. By analysis of the UV spectrum of the compounds from 70 ethanol elution fractions, 20%−3~30%−5 fractions were mixed and selected as *H. citrina* flower buds total phenylpropanoids extract (HFPE). The UV spectrum maximum absorption wavelength of quercetin and isorhamnetin glycosides were around 256 and 356 nm. The absorption maxima of kaempferol glycosides were around 266 and 348 nm. By analysis of the UV spectrum of the compounds from 70 ethanol elution fractions, 30%−6~70%−1 fractions were mixed and selected as *H. citrina* flower buds total flavonoids extract (HFFE). Its final yields were 0.43% (w/w) for HFPE and 0.61% (w/w) for HFFE compared with the crude drugs. The HFPE and HFFE were stored at 4 ℃ before use. UHPLC-Q-TOF-MS was adopted to characterize the constituents in HFPE and HFFE (Figure 3). The compounds were identified by comparing the MS data and retention times with that of the isolated compounds. At last, a total of 13 phenylpropanoids were confirmed from HFPE and a total of 21 flavonoids were identified from HFFE. Compounds **1**, **6**, and **30** were not detected for their low content. The results showed that the main constituents of HFPE were phenylpropanoids and the main constituents of HFFE were flavonoids. The content of each phenylpropanoid compound in HFPE and each flavonoid compound in HFFE were shown in Tables S1 and S2.

### 2.3. Antidepressant Activity of HFPE and HFFE

#### 2.3.1. Body Weight

**Figure 4:**
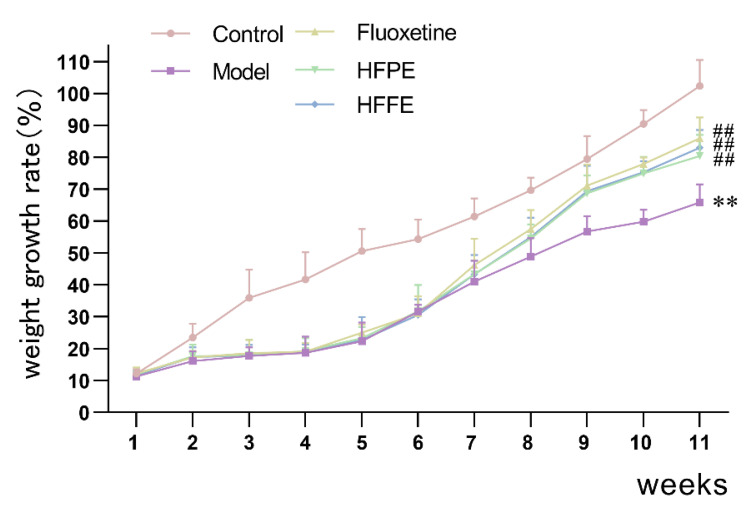
Trends in weight growth rate in each group before and after CUMS treated. The values were represented as mean ± SD (*n* = 10). ** *p* < 0.01 compared with the control group, ^##^ *p* < 0.01 compared with the model group.

As shown in Figure 4, the growth rate of CUMS group’s body weight was significantly lower than that of control group (*p* < 0.01) at the end of the fifth week. However, it was reversed by feeding it 25 mg/kg each of HFPE and HFFE over a period of five weeks. Administration of fluoxetine for 5 weeks also alleviate the reduction in body weight of CUMS rats. In this study, we found CUMS procedure caused slower weight gain, which was consistent with the literature [12,54]. It has been reported that chronic variable stress produced a decrease in body weight along the stress exposure. It may be related to physiological changes, anorexia, or an increase in basal corticosterone [55].

#### 2.3.2. Effects of HFPE and HFFE on Sucrose Preference Test (SPT)

**Figure 5:**
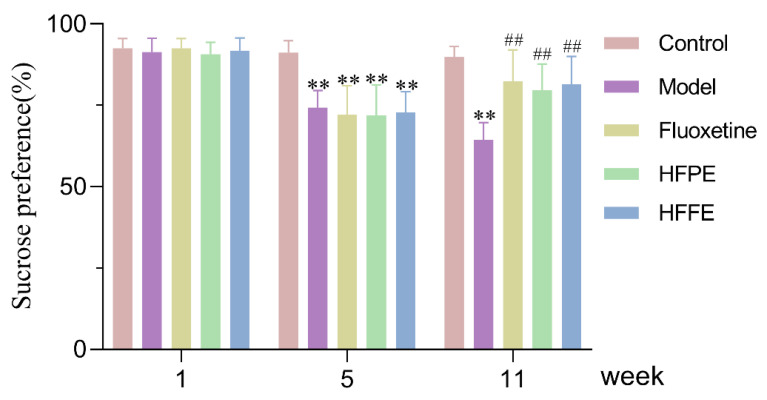
Effects of HFPE and HFFE on the sucrose preference before and after CUMS procedure. The values were represented as mean ± SD (*n* = 10). ** *p* < 0.01 compared with the control group, ^##^ *p* < 0.01 compared with the model group.

Rodents naturally have a strong desire for sweets, however, when the rodents are in a model of CUMS, they are not predisposed to drink sucrose solutions. Therefore, detecting the degree of preference for sucrose solution can be used as a useful means to evaluate the symptoms of anhedonia and the degree of depression in animals [56,57]. As shown in Figure 5, before the CUMS technique, there was not much of a difference between the groups. Sucrose preference was significantly decreased than that of the control group, after 4 weeks of CUMS induced, which indicated that the model was established successfully. After administering fluoxetine, HPPE, and HPFE for five weeks, the fluoxetine group displayed significantly higher sucrose preference (82.40%, *p* < 0.01) in CUMS rats compared to the model group (64.44%). Similarly, oral administration of HFPE and HFFE resulted in significant restoration (*p* < 0.01) of sucrose preference to normal level in CUMS rats. The sucrose preference of the HFPE group was 79.59% and of the HFFE group was 81.47%. The anhedonia-like behavior that CUMS induced in SPT was clearly reversed with the use of HPPE or HPFE.

#### 2.3.3. Effects of HFPE and HFFE on Open-Field Test (OFT)

The OFT was conducted following the SPT. The crossing score (the count of the rats crossing lines) and rearing score (the count of the rats standing up) of rats during a 5 min test could indicate their locomotion activity. OFT was often used to evaluate locomotion, exploratory activity, and anxiety-like behaviors in new environmental conditions [58]. The crossing score could reflect locomotion activity of rats. It was shown that there was no significant difference in crossing score between five groups, which was consistent with discovered in the literature [12]. This indicated that HFPE and HFFE did not affect the spontaneous motor ability of rats.

**Figure 6:**
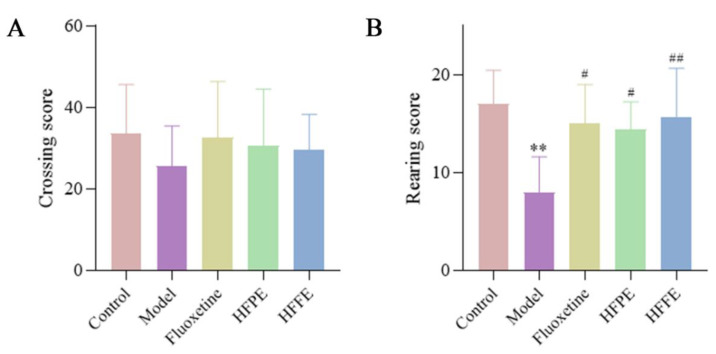
Effects of HFPE and HFFE on OFT test. (**A**) represents crossing score, (**B**) represents rearing score. Results were represented as mean ± SD (*n* = 10). ** *p* < 0.01 compared with the control group, ^#^ *p* < 0.05 and ^##^ *p* < 0.01 compared with the model group.

The rearing score could reflect exploratory activity of rats. The rearing score of depressed rats decreased as a result of a decrease in curiosity or interest in exploring the external environment. The HFPE, HFFE, and fluoxetine treatments improved the rearing score of the CUMS rat, as shown in Figure 6B.

#### 2.3.4. Effects of HFPE and HFFE on the Forced Swimming Test (FST)

**Figure 7:**
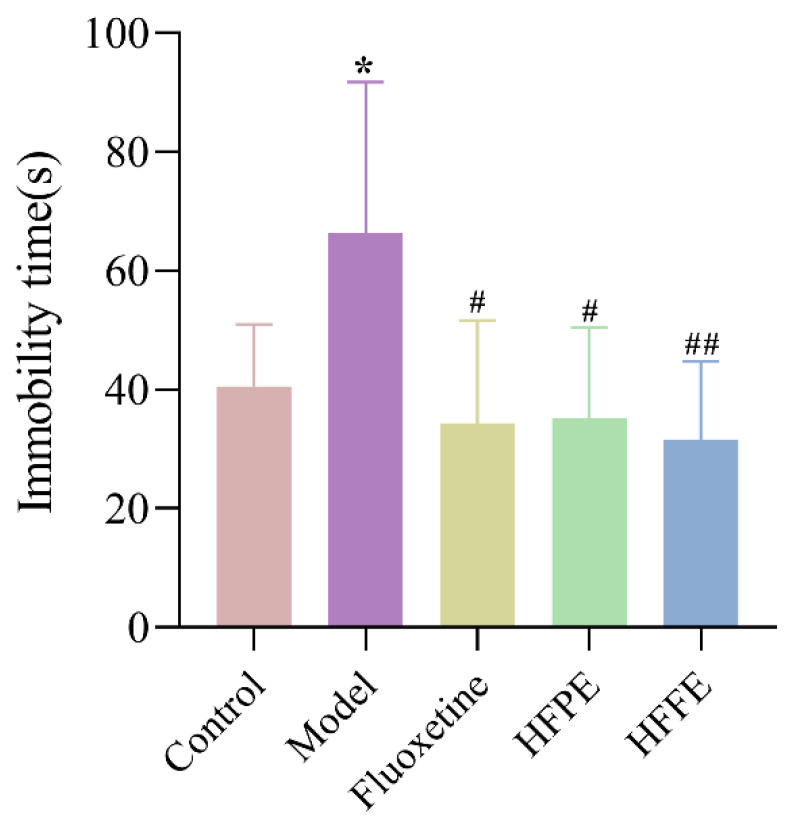
Effects of HFPE and HFFE on FST. Results were represented as mean ± SD (*n* = 10). * *p* < 0.05 compared with the control group, ^#^ *p* < 0.05 and ^##^ *p* < 0.01 compared with the model group.

An immobile posture in the FST reflects a condition of helplessness or despair. FST was often used to assess depression-like behaviors in rats [59]. Figure 7 demonstrated that compared with control group, the immobility time of the CUMS model group was prolonged. The administrations of HFPE, HFFE, or fluoxetine reduced immobility during the FST compared with that in the CUMS model groups. In addition, the crossing score of OFT results revealed that no difference was observed in locomotion activity between the five groups, so HFPE and HFFE indeed have the ability to improve depressive symptoms.

#### 2.3.5. Effects of HFPE and HFFE on Serum Corticosterone (CORT) Level and the Inflammatory Level in Hippocampus

**Figure 8:**
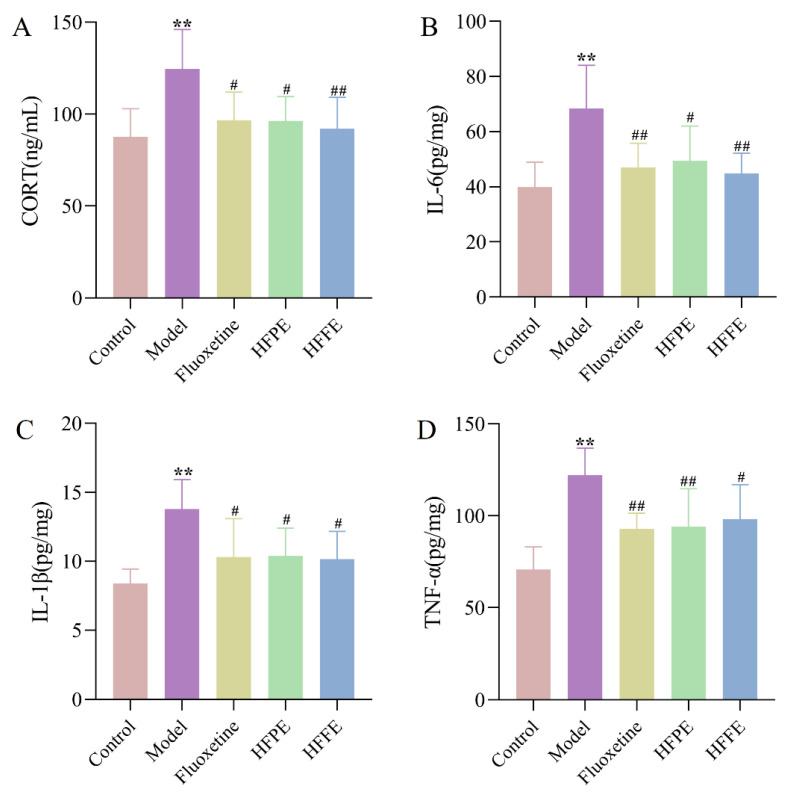
Effects of HFPE and HFFE on serum CORT level and the inflammatory level in hippocampus of CUMS rats. ELISA for detecting CORT (**A**), IL-6 (**B**), IL-1β (**C**), and TNF-α (**D**) levels. Results were represented as mean ± SD (*n* = 8). ** *p* < 0.01 compared with the control group, ^#^ *p* < 0.05 and ^##^ *p* < 0.01 compared with the model group.

As Figure 8A shown, the serum CORT levels were significantly higher in the CUMS group compared to the control group (*p* < 0.01). However, when HFPE and HFFE were administered to CUMS rats, the blood CORT levels significantly decreased. By activating the glucocorticoid receptor and regulating the aberrant activity of the hypothalamus–pituitary–adrenal (HPA) axis, high levels of glucocorticoids may exacerbate depression symptoms [60,61]. Serum CORT is an indicator of depression in laboratory animals. It was shown that CUMS induced an obvious elevation of serum CORT levels in rats, which was consistent with the previous studies [62] and proved the validity of CUMS model. However, HFPE and HFFE treatment could significantly reduce the CORT level in CUMS rats, indicating the alleviation of depression severity.

The results also showed that the levels of IL-6, IL-1β, and TNF-α of the CUMS group were significantly increased compared with those in the control group. However, the levels of the aforementioned pro-inflammatory cytokines were obviously reduced after 5 weeks of therapy with HFPE, HFFE, or fluoxetine (Figure 8). Numerous studies have shown that inflammation plays a significant role in depression. According to the inflammatory theory for depression, stress triggers inflammatory processes, which impair the body’s ability to produce serotonin and regulate the HPA axis, resulting in depression [63]. Pro-inflammatory cytokines—including IL-6, IL-1β, and TNF-α—were increased in depressed patients’ bodies [64]. In this study, CUMS significantly improved the hippocampal levels of IL-6, IL-1β, and TNF-α in rats, while HFPE and HFFE significantly reversed the increase in these pro-inflammatory cytokines. These results implied that the protective effect of HFPE and HFFE on rat behavior may be related to inhibiting the release of pro-inflammatory factors.

### 2.4. Anti-Neuroinflammatory Activity and Structure–Activity Relationship

The anti-neuroinflammatory activity of *H. citrina* flower buds 80% EtOH extract (HFE), HFPE, HFFE, and the 37 isolated compounds were evaluated in LPS-induced BV2 microglial cells model by IC_50_ values of inhibiting NO production as shown in Table 2. The purity of the isolated compounds was more than 95% as calculated from their peak areas of HPLC. Based on the preliminary study, the IC_50_ values of HFPE and HFFE were significantly lower than those of HFE, indicating that anti-neuroinflammatory substances were enriched in HFPE and HFFE. It can be observed that the compounds **3**, **4**, **5**, **7**, **8**, **13**, **16**, **24**, **25**, **28**, **30**, and **37** showed potential anti-neuroinflammatory effects with IC_50_ values less than 100 μM, and compounds **8**, **16**, **24**, **25**, and **30** showed stronger inhibitory effects on NO production in LPS-induced BV2 cells in comparison with the positive drug indomethacin. Thus, this study allows a preliminary discussion of the structure–activity relationship about the role of quinic acid or quinide group of phenylpropanoids, the role of sugar moieties at C-3 of flavonoids, and the role of substituent group at C-3′ of flavonoids.

#### 2.4.1. Role of Quinic Acid or Quinide Group of Phenylpropanoids

In order to investigate the role of quinic acid or quinide group, the activities of compounds **1**, **3**, and **7** were compared. The results indicated that the introduction of quinic acid or quinide group to the coumaroyl increased the activity, as could also be seen from the activities of compounds **2**, **4**, and **8**. Compound **3** (the coumaroyl linked to the C-3 hydroxyl of quinic acid) showed higher activity than compounds **9** and **11** whose connection sites were at C-4 and C-5. It also could be observed that compound **4** (connection site at C-3) had better activity than compounds **10** (connection site at C-4).

#### 2.4.2. Role of Sugar Moieties at C-3 of Flavonoids

It was reported that flavonoid with a hydroxyl at C-3 exhibited a remarkable increase in anti-neuroinflammatory activity [65]. In our results, we compared the anti-neuroinflammatory activities of compounds **16**–**23**, **24**–**29**, and **30**–**37**, it showed that the compounds (**16**, **24**, and **30**) with hydroxyl group at C-3 showed higher activity than compounds (**17**–**23**, **25**–**29**, and **31**–**37**) with sugar moieties at C-3. It concluded that the sugar moieties at C-3 would decrease the activity.

#### 2.4.3. Role of Substituent Group at C-3′ of Flavonoids

To explore the effect of substituent group at C-3′, the activities of compounds **16**, **24**, and **30** were tested. The results showed that compound **24** (IC_50_ = 13.56 μM) with methoxyl at C-3′ showed higher activity than compounds **16** (IC_50_ = 17.48 μM) with hydroxy at C-3′ and **30** (IC_50_ = 21.99 μM) with hydrogen at C-3′. The results suggested that methoxyl or hydroxy at C-3′ played a more important role in the activitythan hydroxy at C-3′.

Although the anti-neuroinflammatory activity of flavonoid glycosides is very weak, they may convert into corresponding aglycones (quercetin, kaempferol, and isorhamnetin) in vivo [66] to exert with strong anti-neuroinflammatory effects. These findings suggest that phenylpropanoids and flavonoids may play a potential inhibitory role in microglia-involved neuroinflammation, thus producing neuroprotective effects in inflammatory-related neuronal diseases including depressive disorder.

## 3. Materials and Methods

### 3.1. Plant Material

*H. citrina* flower buds were provided by Tiancheng Agricultural Development Co. Ltd., Qidong, Hunan, China. A voucher specimen (no. HF20200905) was identified by professor Ying Jia, and deposited at School of Traditional Chinese Materia Medica of Shenyang Pharmaceutical University.

### 3.2. Apparatus and Reagents

HPLC was applied on Agilent 1260 Infinity equipped with an Agilent G1365D multiwavelength detector with a pack of column (YMC-packed C_18_, 250 mm × 10 mm, 5 μm). Methanol (HPLC grade) was bought from Merck Company (Darmstadt, Germany). Silica gel (100–200, 200–300 mesh) was purchased from Qingdao Haiyang Chemical Co., Ltd. (Qingdao, China). ODS XB-C18 (40-70 μm) and Tandex LH-20 (30–120 μm) were bought from Welch Co., Ltd. (Shanghai, China). Petroleum ether, ethyl acetate, ethanol, and dichloromethane were purchased from Tianjin Fuyu chemical Co., Ltd. (Tianjin, China). Fluoxetine tablet was purchased from the Lilly Suzhou Pharmaceutical Co., Ltd. (Suzhou, China). Sodium carboxymethylcellulose (CMC-Na) were purchased from the Tianjin Damao Chemical Reagent Co., Ltd. (Tianjin, China). ELISA kits of CORT, IL-6, IL-1β, and TNF-α were purchased from Shanghai Enzyme-linked Biotechnology Co., Ltd. (Shanghai, China). Cell Counting Kit-8 (CCK8) was purchased from Meilunbio Biotechnology Co., Ltd. (Dalian, China). NO assay kit was purchased from Beyotime Biotechnology Co., Ltd. (Shanghai, China).

### 3.3. Extraction and Isolation

The air-dried flower buds of *H. citrina* (6.0 kg) were extracted with 60 L 80% EtOH by cold-dipping method (three times, 48 h each time). The extract was evaporated under reduced pressure to obtain the residue that was suspended in water. The fraction was subjected to CC (AB macroporous resin; EtOH/H_2_O 0%, 10%, 20%, 30%, 50%, 70%, 90%) to afford seven fractions (Frs.1–7). Fr.3 was subjected to CC (LH-20; MeOH) and afforded two fractions (Frs.3.1–3.2). Fr.3.2 was subjected to CC (reversed-phase C_18_ silica gel; MeOH/H_2_O 10:90→90:10) and afforded four fractions (Frs.3.2.1–3.2.4). Fr.3.2.1 was purified by RP-HPLC with MeOH/H_2_O as mobile phase (22:78) to afford **3** (69.1 mg), **4** (25.3 mg), **6** (3.1 mg), and **15** (13.0 mg). Fr.3.2.2 was purified by RP-HPLC with MeOH/H_2_O as mobile phase (22:78) to afford **5** (2.5 mg) and **10** (23.5 mg). Fr.3.2.3 was purified by RP-HPLC with MeOH/H_2_O as mobile phase (27:72) to afford **12** (5.3 mg), **13** (28.5 mg), and **14** (29.1 mg). Fr.3.2.4 was purified by RP-HPLC with MeOH/H_2_O as mobile phase (35:65) to afford **7** (8.2 mg) and **8** (4.7 mg). Fr.4 was subjected to CC (reversed-phase C_18_ silica gel; MeOH/H_2_O 10:90→90:10) and afforded two fractions (Frs.4.1–4.2). Fr.4.1 was subjected to CC (LH-20; MeOH) and afforded two fractions (Frs.4.1.1–4.1.2). Fr.4.1.1 was purified by RP-HPLC with MeOH/H_2_O as mobile phase (28:72) to afford **1** (15.0 mg). Fr.4.1.1 was purified by RP-HPLC with MeCN/H_2_O as mobile phase (15:85) to afford **2** (2.6 mg). Fr.4.2 was purified by RP-HPLC with MeOH/H_2_O as mobile phase (28:72) to afford **11** (22.0 mg). Fr.4.2 was purified by RP-HPLC with MeCN/H_2_O as mobile phase (15:85) to afford **9** (12.3 mg). Fr.5 was subjected to CC (reversed-phase C_18_ silica gel; MeOH/H_2_O 10:90→90:10) and afforded three fractions (Frs.5.1–5.3). Fr.5.1 was purified by RP-HPLC with MeOH/H_2_O as mobile phase (40:60) to afford **25** (6.2 mg), **35** (13.1 mg). Fr.5.1 was purified by RP-HPLC with MeOH/H_2_O as mobile phase (45:55) to afford **19** (4.3 mg), **21** (6.4 mg), **26** (10.6 mg), **31** (14.2 mg), **32** (6.6 mg), and **33** (6.2 mg). Fr.5.1 was purified by RP-HPLC with MeCN/H_2_O as mobile phase (18:72) to afford **34** (27.3 mg). Fr.5.2 was subjected to CC (LH-20; MeOH) and afforded eight fractions (Frs.5.2.1–5.2.8). Fr.5.2.1 was purified by RP-HPLC with MeOH/H_2_O as mobile phase (37:63) to afford **29** (8.4 mg). Fr.5.2.2 was purified by RP-HPLC with MeCN/H_2_O as mobile phase (19:81) to afford **28** (9.0 mg). Fr.5.2.3 was purified by RP-HPLC with MeOH/H_2_O as mobile phase (30:70) to afford **22** (22.4 mg) and **23** (4.1 mg). Fr.5.2.4 was purified by RP-HPLC with MeCN/H_2_O as mobile phase (13:87) to afford **36** (4.8 mg) and **37** (10.1 mg). Fr.5.2.5 was purified by RP-HPLC with MeCN/H_2_O as mobile phase (19:81) to afford **27** (41.0 mg). Fr.5.2.6 was purified by RP-HPLC with MeCN/H_2_O as mobile phase (18:82) to afford **20** (19.6 mg). Fr.5.2.7 was purified by RP-HPLC with MeOH/H_2_O as mobile phase (45:55) to afford **18** (20.3 mg). Fr.5.2.8 was purified by RP-HPLC with MeOH/H_2_O as mobile phase (43:57) to afford **17** (4.1 mg). Fr.5.3 was subjected to CC (LH-20; MeOH) and afforded three fractions (Frs.5.3.1–5.3.3). Fr.5.3.1 was purified by RP-HPLC with MeOH/H_2_O as mobile phase (53:47) to afford **24** (24.7 mg). Fr.5.3.2 was purified by RP-HPLC with MeOH/H_2_O as mobile phase (53:47) to afford **30** (6.4 mg). Fr.5.3.3 was purified by RP-HPLC with MeOH/H_2_O as mobile phase (53:47) to afford **16** (7.9 mg).

### 3.4. Nuclear Magnetic Resonance Spectrometry (NMR)

One- and two-dimensional NMR experiments were performed on a Bruker Ascend 600 NMR spectrometer (Bremen, Germany), in methanol-*d*_4_, using TMS as an internal standard. Chemical shifts are given in parts per million (*δ*) relative to the residual proton signals of the solvent (MeOH, *δ*_H_ 3.31 and *δ*_C_ 49.00) and coupling constants (*J*) are given in hertz (Hz).

### 3.5. Enrichment of HFPE and HFFE of H. citrina Flower Buds

The air-dried *H. citrina* flower buds (2.5 kg) were extracted with 25 L 80% EtOH by cold-dipping method (three times, 48 h each time). The extract was evaporated under reduced pressure to give a residue that was suspended in water. An appropriate amount of the solution was preserved as *H. citrina* flower buds extract (HFE) to test anti-neuroinflammatory activity. Then, the residual sample was extracted by the same amount *n*-butanol for three times. The *n*-butanol fraction was subjected to CC (AB macroporous resin; EtOH/H_2_O 0%, 10%, 20%, 30%, 50%, 70%, 90%) to afford 70 fractions (10 fractions for each elution solvent). These fractions were injected to 1260 Agilent HPLC system (Agilent, Santa Cara, CA, USA) with a DAD detector. Separation was carried out on a Phenomenex luna C_18_ column (5 μm, 4.6 × 250 mm). Mobile phases were water with 0.1% formic acid (A) and methanol (B). Chromatographic separation was achieved using a gradient elution as follows: 0.01 min, 5%B; 0.01–60 min, 5% to 100% B. The injection volume of sample was 20 μL. The flow rate was 1 mL/min and the column temperature was 35 °C. The fractions were merged under the direction of UV spectra.

### 3.6. UHPLC-MS Detection and Data Analysis

The analyses were performed by UHPLC system (Shimadzu, Kyoto, Japan), which is equipped with a model of LC-30AD pump and a model of SIL-30AC autosampler. The water column (ACQUITY UPLC^®^ HSS T3 1.7 μm, 2.1 × 100 mm) was used for separation. Mobile phases were water with 0.1% of formic acid (A) (Ph = 3.02) and acetonitrile with 0.1% of formic acid (B). Chromatographic separation was achieved using a gradient elution as follows: 0.01–2 min, 2%; 2–16 min, 2% to 20% B; 16–19 min, 20% to 23% B; 19–30 min, 23% to 100% B; 30–33 min, 100% B; 33–33.1 min, 100% to 2% B; 33.1–35 min, 2% B. A sample volume of 4 μL was injected and introduced to the column with 0.4 mL·min^−1^ of the solvent flow rate. The column temperature was set at 35 ℃. MS analysis instrument with a Triple TOF 4600 system (SCIEX, Framingham, MA, USA) was performed in negative mode. The mass range was set at *m/z* 100–1000 Da. The ESI heater temperature was set at 500 °C. The IonSpray Voltage Floating was set at 4500 V. The collision energy and declustering potential energy were set at −10 and −100, respectively. Nebulizer gas, curtain gas, and auxiliary gas were set at 50, 35, and 50 psi. The information-dependent acquisition mode was used for MS/MS ion data acquired. The MS conditions were corrected by APCI negative calibration solution for the AB SCIEX Triple TOF^TM^ systems. PeakView software (version 2.2, AB SCIEX, CA, USA) was used for structural identification of compounds from *H. citrina*.

### 3.7. Antidepressant-Like Effects of HFPE and HFFE

#### 3.7.1. Animals

The Animal Ethics Committee of Shenyang Pharmaceutical University has approved all animal testing (no. SYPU-IACUC-S2021-03-03-202). Male Sprague-Dawley (SD) rats weighing 180–200 g were supplied by Central Animal House of Shenyang Pharmaceutical University (Shenyang, China). Animals were housed in a room that had a 12 h light/dark cycle and a temperature range of 21 to 25 °C. They were also provided water and a regular food. Before the actual trials start, the rats should acclimate to their new environment for seven days.

#### 3.7.2. Groups and Drug Administration

**Figure 9:**
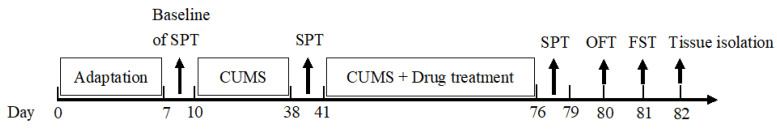
Schedule of the experimental procedure.

A baseline test was conducted as described below before grouping. Rats were divided into five groups of 10 depending on their weight and sucrose preference. The model group, fluoxetine (10 mg/kg) group, HFPE (25 mg/kg) group, and HFFE (25 mg/kg) group were the four treatment groups using CUMS procedures. An untouchable control group was used. Based on what had been reported in the pharmacological literature [2,12] the HFPE and HFFE dosages were selected. Two hours before to the daily CUMS procedure and behavioral testing, the rats received the appropriate solutions. All of the medications were administered through gastric gavage at a volume of 3 mL/kg of body weight and dissolved in sodium carboxymethylcellulose (CMC-Na) at a concentration of 0.3%. The FST, OFT, and SPT were all conducted following the CUMS procedure, as shown in Figure 9. Days 76–79, 80, and 81 after the final dosage of the medication, respectively, saw the completion of the SPT, OFT, and FST. Following the final test, blood samples were collected and centrifuged at 4000 rpm for 10 min. Rat serum and brain tissue were collected and stored at −80 °C for future study.

#### 3.7.3. Body Weight and CUMS Procedure

Body weights were recorded every week. The CUMS procedure was carried out as Willner [67] described with a minor adjustment. CUMS referred to exposure to a variety of different variable stress factors, including: (1) being deprived of food for 24 h, (2) being deprived of water for 24 h, (3) 45° cage tilt for 24 h, (4) being in an empty cage, (5) the light/dark cycle of inversion, (6) 5 min cold swimming (4 °C), (7) 24 h in wet sawdust, (8) horizontal cage shaking for 30 min, (9) 1 min tail nipping, and (10) 4 h physical restraint. The aforementioned stressors were distributed at random to each of the other four rat groups during the course of the experiment’s nine weeks, but not to the blank group.

#### 3.7.4. Sucrose Preference Testing

The SPT is used to evaluate rodent behavior associated with a human clinical symptom of depression by evaluating the capacity to seek pleasure. SPT was carried out in the same manner as previously described [68]. After one week of adapted feeding, the SPT were performed at 4 weeks’ CUMS exposure and the last CUMS exposure. Each rat was given two pre-weighed bottles of either water or a solution containing 1% sucrose (w/v) for 6 h after being without water for 12 h. The bottles were replaced for an additional 6 h. After that, the amount of liquids consumed was recorded. The method used to determine sucrose preference is as follows: consumption of sucrose divided by consumption of sucrose plus water equals sucrose preference.

#### 3.7.5. Open-Field Test

To assess the effects of HFPE and HFFE on exploratory behavior of rats administered CUMS, OFT carried out in the same manner as literature [69]. Each rat was placed in its own square in the center of a black box that measured 100 cm × 100 cm × 40 cm. There were 25 squares on the floor. For five min, the rats were free to walk around. Both the crossing score and the rearing score for each animal were recorded. The apparatus was carefully cleaned with 75% ethanol and rinsed to remove any odors.

#### 3.7.6. Forced Swimming Test

The FST protocol was carried out as described in previous literature [70]. Each rat was placed in a separate transparent bucket filled with 23 ± 2 °C water that was 70 cm high and 40 cm wide. The time the rat’s immobility duration in the last five minutes of their six-minute swim in the bucket was recorded. The apparatus was cleaned after each usage, the rats were dried and returned to their cages.

#### 3.7.7. Determination of Serum CORT Level and IL-6, IL-1β, and TNF-α Level in the Hippocampus

The serum and hippocampal tissue of rats were taken. The tissue was appropriately diluted before the reagents and samples were put one at a time to the microtiter plate in accordance with the ELISA kit’s instructions. The optical density value was calculated after the reaction by setting the microtiter plate at 450 nm. The amount of CORT in the serum and the amounts of IL-6, IL-1β, and TNF-α in the hippocampus tissue were calculated using the standard curve.

### 3.8. Anti-Neuroinflammatory Activity

#### 3.8.1. Cellular Culture

BV2 microglia cells were purchased from iCell Bioscience Inc. (Shanghai, China). The cells were grown at 37 °C in a humid environment with 5% CO_2_. In DMEM with 10% fetal bovine serum and 1% penicillin-streptomycin solution, they were maintained alive. The following assays were carried out while the cells were in the exponential growth phase (subcultured 2–3 times).

#### 3.8.2. CCK8 Cytotoxic Activity

The cytotoxic activity was assessed using the CCK8 test. In a 96-well plate, BV2 microglial cells (5 × 10^4^ cells/well) were placed in each well and incubated for 12 h. After one hour with each substance, LPS (1 μg/mL) was applied to the BV2 microglia cells for an additional 24 h. The CCK8 solution was then incubated with BV2 cells for 1 h at 37 °C. The absorbance at 450 nm was measured using a plate reader (Molecular Devices, San Jose, CA, USA). The cells that had not been treated were assumed to have a 100% optical density.

#### 3.8.3. Inhibition of NO Production

To determine how much NO is produced, the literature [18] was consulted. Equal parts of the Griess reagent (1% sulfanilamide and 0.1% N-(1-naphthyl) ethylenediamine dihydrochloride in 2.5% phosphoric acid) were combined with the sample-treated cell culture medium for 5 min at room temperature and in the dark. It was determined that the absorbance was at 540 nm using a microplate reader. The trials were conducted simultaneously in triplicate.

### 3.9. Statistical Analysis

All data were expressed as the means ± SD from three independent experiments and were analyzed using GraphPad Prism 8.0 software (GraphPad software, San Diego, CA, USA). Statistical analysis was performed by one-way ANOVA with Tukey’s post-hoc test, and *p* < 0.05 was regarded as significant difference.

## 4. Conclusions

In the present study, we describe the isolation and structure identification of bioactive phenylpropanoids and flavonoids from *H. citrina* together with an evaluation of their anti-neuroinflammatory activity. As part of our ongoing interest in discovering active ingredients from natural products, 15 phenylpropanoids and 22 flavonoids were isolated and identified from *H. citrina*. Among them, compound **8** was a novel compound, compounds **1**–**4**, **6**, **9**, **10**, **15**, **17**, **24**–**26**, **28**, and **37** were isolated from *Hemerocallis* genus for the first time.

Importantly, HFPE and HFFE were successfully enriched, and a total of 13 phenylpropanoids were confirmed from HFPE and a total of 21 flavonoids were confirmed from HFFE. According to the present SPT, OFT, and FST studies, HFPE and HFFE can improve depression-like behavior in rats. The biochemistry analyses of serum CORT level and the IL-6, IL-1β, and TNF-α level in hippocampus of CUMS rats indicate that the protective effect of HFPE and HFFE on rat behaviors may be associated with the release of CORT and pro-inflammatory cytokine.

## Figures and Tables

**Table 1:** ^1^H and ^13^C-NMR spectroscopic data of compound **8**.

Position	8	
	*δ*_H_	*δ*_C_
1		73.0
2a	2.03 (1H, t, *J* = 11.8 Hz)	36.8
2b	2.12 (1H, ddd, *J* = 2.7, 6.9, 11.5 Hz)	
3	4.88 (1H, m)	70.0
4	4.28 (1H, t, *J* = 4.6 Hz)	64.7
5	4.72 (1H, t, *J* = 5.4 Hz)	77.7
6a	2.53 (1H, d, *J* = 11.8 Hz)	37.8
6b	2.29 (1H, ddd, *J* = 2.8, 6.0, 11.5 Hz)	
7		178.9
1′		127.5
2′		134.0
3′	6.76 (1H, br d, *J* = 8.7 Hz)	115.8
4′	7.68 (1H, br d, *J* = 8.4 Hz)	134.1
5′	6.76 (1H, br d, *J* = 8.7 Hz)	116.0
6′	7.68 (1H, br d, *J* = 8.4 Hz)	160.3
7′	6.90 (1H, d, *J* = 12.8 Hz)	146.1
8′	5.82 (1H, d, *J* = 12.8 Hz)	116.1
9′		166.9

**Table 2:** Inhibitory effects on NO production induced by LPS in BV2 cells of the compounds or extract from *H. citrina*.

Compounds or Extract	IC_50_ (μM or ug/mL) ^a,b^		Compounds	IC_50_ (μM) ^a,b^	
	NO Inhibitory	Cell Viability		NO Inhibitory	Cell Viability
HFE	497.01 ± 20.45	>100	**19**	>100	>100
HFPE	25.75 ± 5.67	>100	**20**	>100	>100
HFFE	168.52 ± 16.35	>100	**21**	>100	>100
**1**	>100	>100	**22**	>100	>100
**2**	>100	>100	**23**	>100	>100
**3**	78.52 ± 8.23	>100	**24**	13.56 ± 0.66	>100
**4**	70.44 ± 5.86	>100	**25**	48.67 ± 3.75	>100
**5**	95.77 ± 7.18	>100	**26**	>100	>100
**6**	>100	>100	**27**	>100	>100
**7**	94.56 ± 5.62	>100	**28**	90.66 ± 10.37	>100
**8**	36.04 ± 2.78	>100	**29**	>100	>100
**9**	>100	>100	**30**	21.99 ± 2.81	>100
**10**	>100	>100	**31**	>100	>100
**11**	>100	>100	**32**	>100	>100
**12**	>100	>100	**33**	>100	>100
**13**	74.43 ± 6.53	>100	**34**	>100	>100
**14**	>100	>100	**35**	>100	>100
**15**	>100	>100	**36**	>100	>100
**16**	17.48 ± 3.25	>100	**37**	96.11 ± 11.55	>100
**17**	>100	>100	Indomethacin ^c^	52.56 ± 4.58	>100
**18**	>100	>100			

Notes: ^a^ Data were presented as the mean ± SD (*n* = 3). ^b^ The compound test concentrations ranged from 3.125 to 100 μM. HFPE and HFFE test concentrations ranged from 6.25 to 200 μg/mL, HFE test concentrations ranged from 62.5 to 2000 μg/mL. ^c^ Positive control was set to be indomethacin.

## Data Availability

Not available.

## Supplementary Materials

The following supporting information can be downloaded at: https://www.mdpi.com/article/10.3390/molecules27185809/s1, Figure S1. The HR-ESI-MS spectrum of compound **1**; Figure S2: The 1H-NMR (600 MHz, CD3OD) spectrum of compound **1**; Figure S3: The 13C-NMR spectrum (150 MHz, CD3OD) of compound **1**; Figure S4: The ESI-MS spectrum of compound **2**; Figure S5: The 1H-NMR (600 MHz, CD3OD) spectrum of compound **2**; Figure S6: The 13C-NMR spectrum (150 MHz, CD3OD) of compound **2**; Figure S7: The HR-ESI-MS spectrum of compound **3**; Figure S8: The 1H-NMR (600 MHz, CD3OD) spectrum of compound **3**; Figure S9: The 13C-NMR spectrum (150 MHz, CD3OD) of compound **3**; Figure S10: The HR-ESI-MS spectrum of compound **4**; Figure S11: The 1H-NMR (600 MHz, CD3OD) spectrum of compound **4**; Figure S12: The 13C-NMR spectrum (150 MHz, CD3OD) of compound **4**; Figure S13: The 1H-NMR (600 MHz, CD3OD) spectrum of compound **5**; Figure S14: The 13C-NMR spectrum (150 MHz, CD3OD) of compound **5**; Figure S15: The HR-ESI-MS spectrum of compound **6**; Figure S16: The 1H-NMR (600 MHz, CD3OD) spectrum of compound **6**; Figure S17: The 13C-NMR spectrum (150 MHz, CD3OD) of compound **6**; Figure S18: The HR-ESI-MS spectrum of compound **7**; Figure S19: The 1H-NMR (600 MHz, CD3OD) spectrum of compound **7**; Figure S20: The 13C-NMR spectrum (150 MHz, CD3OD) of compound **7**; Figure S21: The HR-ESI-MS spectrum of compound **8**; Figure S22: The 1H-NMR (600 MHz, CD3OD) spectrum of compound **8**; Figure S23: The 13C-NMR spectrum (150 MHz, CD3OD) of compound **8**; Figure S24: The HSQC spectrum (CD3OD) of compound **8**; Figure S25: The HMBC spectrum (CD3OD) of compound **8**; Figure S26: The HR-ESI-MS spectrum of compound **9**; Figure S27: The 1H-NMR (600 MHz, CD3OD) spectrum of compound **9**; Figure S28: The 13C-NMR spectrum (150 MHz, CD3OD) of compound **9**; Figure S29: The HR-ESI-MS spectrum of compound **10**; Figure S30: The 1H-NMR (600 MHz, CD3OD) spectrum of compound **10**; Figure S31: The 13C-NMR spectrum (150 MHz, CD3OD) of compound **10**; Figure S32: The HR-ESI-MS spectrum of compound **11**; Figure S33: The 1H-NMR (600 MHz, CD3OD) spectrum of compound **11**; Figure S34: The 13C-NMR spectrum (150 MHz, CD3OD) of compound **11**; Figure S35: The HR-ESI-MS spectrum of compound **12**; Figure S36: The 1H-NMR (600 MHz, CD3OD) spectrum of compound **12**; Figure S37: The 13C-NMR spectrum (150 MHz, CD3OD) of compound **12**; Figure S38: The HR-ESI-MS spectrum of compound **13**; Figure S39: The 1H-NMR (600 MHz, CD3OD) spectrum of compound **13**; Figure S40: The 13C-NMR spectrum (150 MHz, CD3OD) of compound **13**; Figure S41: The HR-ESI-MS spectrum of compound **14**; Figure S42: The 1H-NMR (600 MHz, CD3OD) spectrum of compound **14**; Figure S43: The 13C-NMR spectrum (150 MHz, CD3OD) of compound **14**; Figure S44: The ESI-MS spectrum of compound **15**; Figure S45: The 1H-NMR (600 MHz, CD3OD) spectrum of compound **15**; Figure S46: The 13C-NMR spectrum (150 MHz, CD3OD) of compound **15**; Figure S47: The HR-ESI-MS spectrum of compound **16**; Figure S48: The 1H-NMR (600 MHz, DMSO-*d*6) spectrum of compound **16**; Figure S49: The 13C-NMR spectrum (150 MHz, DMSO-*d*6) of compound **16**; Figure S50: The HR-ESI-MS spectrum of compound **17**; Figure S51: The 1H-NMR (600 MHz, CD3OD) spectrum of compound **17**; Figure S52: The 13C-NMR spectrum (150 MHz, CD3OD) of compound **17**; Figure S53: The HR-ESI-MS spectrum of compound **18**; Figure S54: The 1H-NMR (600 MHz, CD3OD) spectrum of compound **18**; Figure S55: The 13C-NMR spectrum (150 MHz, CD3OD) of compound **18**; Figure S56: The HR-ESI-MS spectrum of compound **19**; Figure S57: The 1H-NMR (600 MHz, CD3OD) spectrum of compound **19**; Figure S58: The 13C-NMR spectrum (150 MHz, CD3OD) of compound **19**; Figure S59: The HR-ESI-MS spectrum of compound **20**; Figure S60: The 1H-NMR (600 MHz, CD3OD) spectrum of compound **20**; Figure S61: The 13C-NMR spectrum (150 MHz, CD3OD) of compound **20**; Figure S62: The ESI-MS spectrum of compound **21**; Figure S63: The 1H-NMR (600 MHz, CD3OD) spectrum of compound **21**; Figure S64: The 13C-NMR spectrum (150 MHz, CD3OD) of compound **21**; Figure S65: The ESI-MS spectrum of compound **22**; Figure S66: The 1H-NMR (600 MHz, CD3OD) spectrum of compound **22**; Figure S67: The 13C-NMR spectrum (150 MHz, CD3OD) of compound **22**; Figure S68: The ESI-MS spectrum of compound **23**; Figure S69: The 1H-NMR (600 MHz, CD3OD) spectrum of compound **23**; Figure S70: The 13C-NMR spectrum (150 MHz, CD3OD) of compound **23**; Figure S71: The HR-ESI-MS spectrum of compound **24**; Figure S72: The 1H-NMR (600 MHz, CD3OD) spectrum of compound **24**; Figure S73: The 13C-NMR spectrum (150 MHz, DMSO-*d*6) of compound **24**; Figure S74: The ESI-MS spectrum of compound **25**; Figure S75: The 1H-NMR (600 MHz, CD3OD) spectrum of compound **25**; Figure S76: The 13C-NMR spectrum (150 MHz, CD3OD) of compound **25**; Figure S77: The HR-ESI-MS spectrum of compound **26**; Figure S78: The 1H-NMR (600 MHz, CD3OD) spectrum of compound **26**; Figure S79: The 13C-NMR spectrum (150 MHz, CD3OD) of compound **26**; Figure S80: The HR-ESI-MS spectrum of compound **27**; Figure S81: The 1H-NMR (600 MHz, CD3OD) spectrum of compound **27**; Figure S82: The 13C-NMR spectrum (150 MHz, CD3OD) of compound **27**; Figure S83: The ESI-MS spectrum of compound **28**; Figure S84: The 1H-NMR (600 MHz, CD3OD) spectrum of compound **28**; Figure S85: The 13C-NMR spectrum (150 MHz, CD3OD) of compound **28**; Figure S86: The HR-ESI-MS spectrum of compound **29**; Figure S87: The 1H-NMR (600 MHz, CD3OD) spectrum of compound **29**; Figure S88: The 13C-NMR spectrum (150 MHz, CD3OD) of compound **29**; Figure S89: The HR-ESI-MS spectrum of compound **30**; Figure S90: The 1H-NMR (600 MHz, CD3OD) spectrum of compound **30**; Figure S91: The 13C-NMR spectrum (150 MHz, CD3OD) of compound **30**; Figure S92: The HR-ESI-MS spectrum of compound **31**; Figure S93: The 1H-NMR (600 MHz, CD3OD) spectrum of compound **31**; Figure S94: The 13C-NMR spectrum (150 MHz, CD3OD) of compound **31**; Figure S95: The ESI-MS spectrum of compound **32**; Figure S96: The 1H-NMR (600 MHz, CD3OD) spectrum of compound **32**; Figure S97: The 13C-NMR spectrum (150 MHz, CD3OD) of compound **32**; Figure S98: The HR-ESI-MS spectrum of compound **33**; Figure S99: The 1H-NMR (600 MHz, CD3OD) spectrum of compound **33**; Figure S100: The 13C-NMR spectrum (150 MHz, CD3OD) of compound **33**; Figure S101: The HR-ESI-MS spectrum of compound **34**; Figure S102: The 1H-NMR (600 MHz, CD3OD) spectrum of compound **34**; Figure S103: The 13C-NMR spectrum (150 MHz, CD3OD) of compound **34**; Figure S104: The ESI-MS spectrum of compound **35**; Figure S105: The 1H-NMR (600 MHz, CD3OD) spectrum of compound **35**; Figure S106: The 13C-NMR spectrum (150 MHz, CD3OD) of compound **35**; Figure S107: The ESI-MS spectrum of compound **36**; Figure S108: The 1H-NMR (600 MHz, CD3OD) spectrum of compound **36**; Figure S109: The 13C-NMR spectrum (150 MHz, CD3OD) of compound **36**; Figure S110: The ESI-MS spectrum of compound **37**; Figure S111: The 1H-NMR (600 MHz, CD3OD) spectrum of compound **37**; Figure S112: The 13C-NMR spectrum (150 MHz, CD3OD) of compound **37**; Figure S113: The UV spectrum of compound **1**–**37**; Figure S114: The UHPLC chromatogram of reference substance (A), HFPE(B) and HFPE(C); Table S1: The phenylpropanoids compounds content in HFPE; Table S2: The flavonoids compounds content in HFFE [71].

## Sample Availability

Samples of the compounds are available from the authors.
