# Supplementary material for: An Investigation of the Anti-Depressive Properties of Phenylpropanoids and Flavonoids in Hemerocallis citrina Baroni
Source: Molecules. 2022 Sep 8;27(18):5809. doi: 10.3390/molecules27185809 (PMC9502271; doi:10.3390/molecules27185809)
Supplement: Supplementary file 1 [file molecules-27-05809-s001.zip › molecules-1882083-supplementary.pdf]

Supplementary materials An investigation of the anti-depressive properties of phenylpropanoids and flavonoids in *Hemerocallis citrina* Baroni

Tiancheng Ma[1,2], Yu Sun[2], Lida Wang[1], Jinyu Wang[3], Bo Wu[3], Tingxu Yan[3], Ying Jia[3*]

[1] *School of Traditional Chinese Materia Medica, Shenyang Pharmaceutical University, Wenhua Road 103, Shenyang 110016, PR China*

[2] *Research Institute of Medicine and Pharmacy, Qiqihar Medical University, Bukui North Street 333, Qiqihar 161006, PR China*

[3] *School of Functional Food and wine, Shenyang Pharmaceutical University, Wenhua Road 103, Shenyang 110016, PR China*

[*] *Corresponding author*

Ying Jia − *School of Functional Food and wine, Shenyang Pharmaceutical University, Wenhua Road 103, Shenyang 110016, PR China; E-mail:jysyphu@126.com;Tel: +86 24 2398 6933*;

Contents

The NMR data about the known compounds......1

Figure S1. The HR-ESI-MS spectrum of compound 1......12

Figure S2. The ^{1}H-NMR (600MHz, CD_3OD) spectrum of compound 1......12

Figure S3. The ^{13}C-NMR spectrum (150MHz, CD_3OD) of compound 1......12

Figure S4. The ESI-MS spectrum of compound 2......13

Figure S5. The ^{1}H-NMR (600MHz, CD_3OD) spectrum of compound 2......13

Figure S6. The ^{13}C-NMR spectrum (150MHz, CD_3OD) of compound 2......13

Figure S7. The HR-ESI-MS spectrum of compound 3......14

Figure S8. The ^{1}H-NMR (600MHz, CD_3OD) spectrum of compound 3......14

Figure S9. The ^{13}C-NMR spectrum (150MHz, CD_3OD) of compound 3......14

Figure S10. The HR-ESI-MS spectrum of compound 4......15

Figure S11. The ^{1}H-NMR (600MHz, CD_3OD) spectrum of compound 4......15

Figure S12. The ^{13}C-NMR spectrum (150MHz, CD_3OD) of compound 4......15

Figure S13. The ^{1}H-NMR (600MHz, CD_3OD) spectrum of compound 5......16

Figure S14. The ^{13}C-NMR spectrum (150MHz, CD_3OD) of compound 5......16

Figure S15. The HR-ESI-MS spectrum of compound 6......17

Figure S16. The ^{1}H-NMR (600MHz, CD_3OD) spectrum of compound 6......17

Figure S17. The ^{13}C-NMR spectrum (150MHz, CD_3OD) of compound 6......17

Figure S18. The HR-ESI-MS spectrum of compound 7......18

Figure S19. The ^{1}H-NMR (600MHz, CD_3OD) spectrum of compound 7......18

Figure S20. The ^{13}C-NMR spectrum (150MHz, CD_3OD) of compound 7......18

Figure S21. The HR-ESI-MS spectrum of compound 8......19

Figure S22. The ^{1}H-NMR (600MHz, CD_3OD) spectrum of compound 8......19

Figure S23. The ^{13}C-NMR spectrum (150MHz, CD_3OD) of compound 8......19

Figure S24. The HSQC spectrum (CD_3OD) of compound 8......20

Figure S25. The HMBC spectrum (CD_3OD) of compound 8......20

Figure S26. The HR-ESI-MS spectrum of compound 9......21

Figure S27. The ^{1}H-NMR (600MHz, CD_3OD) spectrum of compound 9......21

Figure S28. The ^{13}C-NMR spectrum (150MHz, CD_3OD) of compound 9......21

Figure S29 The HR-ESI-MS spectrum of compound 10......22

Figure S30. The ^{1}H-NMR (600MHz, CD_3OD) spectrum of compound 10......22

Figure S31. The ^{13}C-NMR spectrum (150MHz, CD_3OD) of compound 10......22

Figure S32. The HR-ESI-MS spectrum of compound 11......23

Figure S33. The ^{1}H-NMR (600MHz, CD_3OD) spectrum of compound 11......23

Figure S34. The ^{13}C-NMR spectrum (150MHz, CD_3OD) of compound 11......23

Figure S35. The HR-ESI-MS spectrum of compound 12......24

Figure S36. The ^{1}H-NMR (600MHz, CD_3OD) spectrum of compound 12......24

Figure S37. The ^{13}C-NMR spectrum (150MHz, CD_3OD) of compound 12......24

Figure S38. The HR-ESI-MS spectrum of compound 13......25

Figure S39. The ^{1}H-NMR (600MHz, CD_3OD) spectrum of compound 13......25

Figure S40. The ^{13}C-NMR spectrum (150MHz, CD_3OD) of compound 13......25

Figure S41. The HR-ESI-MS spectrum of compound 14......26

Figure S42. The ^{1}H-NMR (600MHz, CD_3OD) spectrum of compound 14......26

Figure S43. The ^{13}C-NMR spectrum (150MHz, CD_3OD) of compound 14......26

Figure S44. The ESI-MS spectrum of compound 15......27

Figure S45. The ^{1}H-NMR (600MHz, CD_3OD) spectrum of compound 15......27

Figure S46. The ^{13}C-NMR spectrum (150MHz, CD_3OD) of compound 15......27

Figure S47. The HR-ESI-MS spectrum of compound 16......28

Figure S48. The ^{1}H-NMR (600MHz, DMSO-d_6) spectrum of compound 16......28

Figure S49. The ^{13}C-NMR spectrum (150MHz, DMSO-d_6) of compound 16......28

Figure S50. The HR-ESI-MS spectrum of compound 17......29

Figure S51. The ^{1}H-NMR (600MHz, CD_3OD) spectrum of compound 17......29

Figure S52. The ^{13}C-NMR spectrum (150MHz, CD_3OD) of compound 17......29

Figure S53. The HR-ESI-MS spectrum of compound 18......30

Figure S54. The ^{1}H-NMR (600MHz, CD_3OD) spectrum of compound 18......30

Figure S55. The ^{13}C-NMR spectrum (150MHz, CD_3OD) of compound 18......30

Figure S56. The HR-ESI-MS spectrum of compound 19......31

Figure S57. The ^{1}H-NMR (600MHz, CD_3OD) spectrum of compound 19......31

Figure S58. The ^{13}C-NMR spectrum (150MHz, CD_3OD) of compound 19......31

Figure S59. The HR-ESI-MS spectrum of compound 20..........32

Figure S60. The ^{1}H-NMR (600MHz, CD_3OD) spectrum of compound 20..........32

Figure S61. The ^{13}C-NMR spectrum (150MHz, CD_3OD) of compound 20..........32

Figure S62. The ESI-MS spectrum of compound 21..........33

Figure S63. The ^{1}H-NMR (600MHz, CD_3OD) spectrum of compound 21..........33

Figure S64. The ^{13}C-NMR spectrum (150MHz, CD_3OD) of compound 21..........33

Figure S65. The ESI-MS spectrum of compound 22..........34

Figure S66. The ^{1}H-NMR (600MHz, CD_3OD) spectrum of compound 22..........34

Figure S67. The ^{13}C-NMR spectrum (150MHz, CD_3OD) of compound 22..........34

Figure S68. The ESI-MS spectrum of compound 23..........35

Figure S69. The ^{1}H-NMR (600MHz, CD_3OD) spectrum of compound 23..........35

Figure S70. The ^{13}C-NMR spectrum (150MHz, CD_3OD) of compound 23..........35

Figure S71. The HR-ESI-MS spectrum of compound 24..........36

Figure S72. The ^{1}H-NMR (600MHz, CD_3OD) spectrum of compound 24..........36

Figure S73. The ^{13}C-NMR spectrum (150MHz, DMSO-d_6) of compound 24..........36

Figure S74. The ESI-MS spectrum of compound 25..........37

Figure S75. The ^{1}H-NMR (600MHz, CD_3OD) spectrum of compound 25..........37

Figure S76. The ^{13}C-NMR spectrum (150MHz, CD_3OD) of compound 25..........37

Figure S77. The HR-ESI-MS spectrum of compound 26..........38

Figure S78. The ^{1}H-NMR (600MHz, CD_3OD) spectrum of compound 26..........38

Figure S79. The ^{13}C-NMR spectrum (150MHz, CD_3OD) of compound 26..........38

Figure S80. The HR-ESI-MS spectrum of compound 27..........39

Figure S81. The ^{1}H-NMR (600MHz, CD_3OD) spectrum of compound 27..........39

Figure S82. The ^{13}C-NMR spectrum (150MHz, CD_3OD) of compound 27..........39

Figure S83. The ESI-MS spectrum of compound 28..........40

Figure S84. The ^{1}H-NMR (600MHz, CD_3OD) spectrum of compound 28..........40

Figure S85. The ^{13}C-NMR spectrum (150MHz, CD_3OD) of compound 28..........40

Figure S86. The HR-ESI-MS spectrum of compound 29..........41

Figure S87. The ^{1}H-NMR (600MHz, CD_3OD) spectrum of compound 29..........41

Figure S88. The ^{13}C-NMR spectrum (150MHz, CD_3OD) of compound 29..........41

Figure S89. The HR-ESI-MS spectrum of compound 30..42
Figure S90. The ^{1}H-NMR (600MHz, CD_3OD) spectrum of compound 30...................................42
Figure S91. The ^{13}C-NMR spectrum (150MHz, CD_3OD) of compound 30.................................42
Figure S92. The HR-ESI-MS spectrum of compound 31..43
Figure S93. The ^{1}H-NMR (600MHz, CD_3OD) spectrum of compound 31...................................43
Figure S94. The ^{13}C-NMR spectrum (150MHz, CD_3OD) of compound 31.................................43
Figure S95. The ESI-MS spectrum of compound 32..44
Figure S96. The ^{1}H-NMR (600MHz, CD_3OD) spectrum of compound 32...................................44
Figure S97. The ^{13}C-NMR spectrum (150MHz, CD_3OD) of compound 32.................................44
Figure S98. The HR-ESI-MS spectrum of compound 33..45
Figure S99. The ^{1}H-NMR (600MHz, CD_3OD) spectrum of compound 33...................................45
Figure S100. The ^{13}C-NMR spectrum (150MHz, CD_3OD) of compound 33...............................45
Figure S101. The HR-ESI-MS spectrum of compound 34..46
Figure S102. The ^{1}H-NMR (600MHz, CD_3OD) spectrum of compound 34.................................46
Figure S103. The ^{13}C-NMR spectrum (150MHz, CD_3OD) of compound 34...............................46
Figure S104. The ESI-MS spectrum of compound 35..47
Figure S105. The ^{1}H-NMR (600MHz, CD_3OD) spectrum of compound 35.................................47
Figure S106. The ^{13}C-NMR spectrum (150MHz, CD_3OD) of compound 35...............................47
Figure S107. The ESI-MS spectrum of compound 36..48
Figure S108. The ^{1}H-NMR (600MHz, CD_3OD) spectrum of compound 36.................................48
Figure S109. The ^{13}C-NMR spectrum (150MHz, CD_3OD) of compound 36...............................48
Figure S110. The ESI-MS spectrum of compound 37..49
Figure S111. The ^{1}H-NMR (600MHz, CD_3OD) spectrum of compound 37.................................49
Figure S112. The ^{13}C-NMR spectrum (150MHz, CD_3OD) of compound 37...............................49
Figure S113. The UV spectrum of compound 1-37...50
Figure S114 The UHPLC chromatogram of reference substance (A), HFPE(B) and HFPE(C).51
Table S1 The phenylpropanoids compounds content in HFPE...52Table S2 The flavonoids compounds content in HFFE...53
Determination of total phenolic...54

The NMR data about the known compounds

*(E)-p-coumaric acid (**1**)*, yellow oil, $C_9H_8O_3$. HR-ESI-MS (negative) *m/z* 163.0400 $[M-H]^-$ (calcd. for 163.0395). UV (MeOH) λ_{max} 226, 310 nm.^{1}H-NMR (600 MHz, CD_3OD): δ_H 6.28 (1H, d, *J* = 15.9, H-8), 6.79 (2H, d, *J* = 8.6 Hz, H-3, 5), 7.44 (2H, d, *J* = 8.6 Hz, H-2, 6), 7.59 (1H, d, *J* = 15.9 Hz, H-7). ^{13}C-NMR (150 MHz, CD_3OD): δ_C 116.1 (C-8), 116.8 (C-3, 5), 127.3 (C-1), 131.0 (C-2, 6), 146.3 (C-7), 161.1 (C-4), 171.4 (C-9).

*(Z)-p-coumaric acid (**2**)*, yellow oil, $C_9H_8O_3$. ESI-MS (negative) *m/z* 163.00 $[M-H]^-$. UV (MeOH) λ_{max} 296 nm. ^{1}H-NMR (600 MHz, CD_3OD): δ_H 5.81 (1H, d, *J* = 12.7 Hz, H-8), 6.60 (1H, d, *J* = 12.7 Hz, H-7), 6.71(2H, d, *J* = 8.4 Hz, H-3, 5), 7.54 (2H, d, *J* = 8.4 Hz, H-2, 6). ^{13}C-NMR (150 MHz, CD_3OD): δ_C 115.8 (C-3, 5), 116.8 (C-8), 128.4 (C-1), 132.7 (C-2, 6), 139.1 (C-7), 159.2 (C-4).

*3-O-(E)-p-coumaroylquinic acid (**3**)*, colorless oil, $C_{16}H_{18}O_8$. HR-ESI-MS (negative) *m/z* 337.0938 $[M-H]^-$ (calcd. for 337.0923). UV (MeOH) λ_{max} 226, 312 nm. ^{1}H-NMR (600 MHz, CD_3OD): δ_H 1.95 (1H, dd, *J* = 13.5, 10.0 Hz, H-6ax), 2.14 (2H, m, H-2eq, H-6eq), 2.20 (1H, dd, *J* = 14.8, 3.4 Hz, H-2ax), 3.63 (1H, dd, *J* = 8.5, 3.4 Hz, H-4), 4.15(1H, m, H-5), 5.35(1H, dd, *J* = 7.6, 3.6 Hz, H-3), 6.36 (1H, d, *J* = 16.0 Hz, H-8′), 6.80 (2H, d, *J* = 8.6 Hz, H-3′, 5′), 7.45 (2H, d, *J* = 8.6Hz, H-2′, 6′), 7.65(1H, d, *J* = 16.0 Hz, H-7′). ^{13}C-NMR (150 MHz, CD_3OD): δ_C 36.7 (C-2), 41.5 (C-6), 68.3 (C-5), 73.0 (C-3), 74.8 (C-4), 75.4 (C-1), 115.9 (C-8′), 116.8 (C-3′, 5′), 127.4 (C-1′), 131.1 (C-2′, 6′), 146.4 (C-7′), 161.1 (C-4′), 169.0 (C-9′), 178.3 (C-7).

*3-O-(Z)-p-coumaroylquinic acid (**4**)*, colorless oil, $C_{16}H_{18}O_8$. HR-ESI-MS (negative) *m/z* 337.0935 $[M-H]^-$ (calcd. for 337.0923). UV (MeOH) λ_{max} 306 nm. ^{1}H-NMR (600 MHz, CD_3OD): δ_H 1.95-2.20(4H, m, H-2, 6), 3.64 (1H, dd, *J* = 3.3, 8.2 Hz, H-4), 4.08 (1H, m, H-3), 5.31 (1H, dd, *J* = 7.9, 3.7Hz, H-5), 5.83 (1H, d, *J* = 12.8 Hz, H-8′), 6.73 (2H, d, *J* = 8.7 Hz, H-3′, 5′), 6.82 (1H, d, *J* = 12.9Hz, H-7′), 7.68 (2H, d, *J* = 8.7 Hz, H-2′, 6′). ^{13}C-NMR (150 MHz, CD_3OD): δ_C 36.6 (C-6), 41.3 (C-2), 68.4 (C-3), 72.7 (C-4), 74.5 (C-5), 75.4 (C-1), 115.8 (C-3′, 5′), 117.3 (C-8′), 127.7 (C-1′), 133.8 (C-2′, 6′), 144.8 (C-7′), 160.0 (C-4′), 167.9 (C-9′), 178.4 (C-7).

*3-O-(E)-p-coumaroylquinic acid methyl ester (**5**)*, white powder, $C_{17}H_{20}O_8$. UV (MeOH) λ_{max} 226, 312 nm. ^{1}H-NMR (600 MHz, CD_3OD): δ_H 2.04 (1H, m, H-2ax), 2.10 (1H, m, H-2eq), 2.14 (1H, m, H-6 eq), 2.24 (1H, dd, *J* = 14.0, 3.8 Hz, H-6ax), 3.70 (1H, dd, *J* = 7.7, 3.2 Hz, H-4), 3.75 (3H, s, H-8), 4.11 (1H, td, *J* = 8.2, 4.0 Hz, H-3), 5.35 (1H, m, H-5), 6.38 (1H, d, *J* = 15.9 Hz, H-8′), 6.82 (2H, d, *J* = 8.6 Hz, H-3′, 5′), 7.48 (2H, d, *J* = 8.6 Hz, H-2′, 6′), 7.67 (1H, d, *J* = 15.9 Hz, H-7′). ^{13}C-NMR (150 MHz, CD_3OD): δ_C 36.4 (C-6), 40.8 (C-2), 52.9 (C-8), 68.6 (C-3), 72.6 (C-5), 73.8 (C-1), 75.3

(C-4), 115.8 (C-8′), 116.8 (C-3′, 5′), 127.4 (C-1′), 131.1 (C-2′, 6′), 146.5 (C-7′), 161.2 (C-4′), 168.9 (C-9′), 176.4 (C-7).

*3-O-(Z)-p-coumaroylquinic acid methyl ester (**6**)*, white powder, $C_{17}H_{20}O_8$. ESI-MS (negative) *m/z* 351.16 $[M-H]^-$. UV (MeOH) λ_{max} 210, 306 nm. ^{1}H-NMR (600 MHz, CD_3OD): δ_H 2.04 (1H, m, H-2ax), 2.10 (1H, m, H-2eq), 2.14 (1H, m, H-6eq), 2.24 (1H, dd, J = 14.0, 3.8 Hz, H-6ax), 3.69 (1H, dd, J = 7.3, 3.1 Hz, H-4), 3.73 (3H, s, H-8), 4.06 (1H, td, J = 7.7, 4.1 Hz, H-5), 5.32 (1H, m, H-3), 5.84 (1H, d, J = 12.8 Hz, H-8′), 6.74 (2H, d, J = 8.7 Hz, H-3′, 5′), 6.84 (1H, d, 12.8, H-7′), 7.68 (2H, d, J = 8.7 Hz, H-2′, 6′). ^{13}C-NMR (150 MHz, CD_3OD): δ_C 36.2 (C-6), 40.6 (C-2), 52.8 (C-8), 68.7 (C-5), 72.3 (C-3), 73.5 (C-1), 75.2 (C-4), 115.8 (C-8′), 117.2 (C-3′, 5′), 127.7 (C-1′), 133.8 (C-2′, 6′), 144.9 (C-7′), 160.0 (C-4′), 167.8 (C-9′), 176.4 (C-7).

3-O-(E)-p-coumaroylquinide (7), white powder, $C_{16}H_{16}O_7$. ESI-MS (negative) *m/z* 319.0821 $[M-H]^-$ (calcd. for 319.0818). UV (MeOH) λ_{max} 228, 314 nm. ^{1}H-NMR (600 MHz, CD_3OD): 2.09 (1H, t, J = 11.6 Hz, H-2ax), 2.16 (1H, ddd, J = 2.6, 6.7, 11.5 Hz, H-2eq), 2.30 (1H, ddd, J = 2.7, 6.0, 11.5 Hz, H-6eq), 2.56 (1H, d, J = 11.6 Hz, H-6ax), 4.29 (1H, t, J = 4.6 Hz, H-4eq), 4.74 (1H, t, J = 5.4 Hz, H-5eq), 4.92 (1H, m, H-3ax), 6.36 (1H, d, J = 15.9 Hz, H-8′), 6.81 (2H, br d, J = 8.6 Hz, H-3′, 5′), 7.47 (2H, br d, J = 8.6 Hz, H-2′, 6′), 7.68 (1H, d, J = 16.0, H-7′). ^{13}C-NMR (150 MHz, CD_3OD): δ_C 36.9 (C-2), 37.8 (C-6), 64.8 (C-4), 70.2 (C-3), 73.0 (C-1), 77.6 (C-5), 114.8 (C-8′), 116.8 (C-3′, 5′), 127.1 (C-1′), 131.2 (C-2′, 6′), 147.2 (C-7′), 161.4 (C-4′), 168.0 (C-9′), 178.9 (C-7).

*3-O-(Z)-p-coumaroylquinide (**8**)*, white powder, $C_{16}H_{16}O_7$. ESI-MS (negative) *m/z* 319.0824 $[M-H]^-$ (calcd. for 319.0818). UV (MeOH) λ_{max} 310 nm. ^{1}H-NMR (600 MHz, CD_3OD): 2.03 (1H, t, J = 11.8 Hz, H-2ax), 2.12 (1H, ddd, J = 2.7, 6.9, 11.5 Hz, H-2eq), 2.29 (1H, ddd, J = 2.8, 6.0, 11.5 Hz, H-6eq), 2.53 (1H, d, J = 11.8 Hz, H-6ax), 4.28 (1H, t, J = 4.6 Hz, H-4eq), 4.72 (1H, t, J = 5.4 Hz, H-5eq), 4.88 (1H, m, H-3ax), 5.82 (1H, d, J = 12.8 Hz, H-8′), 6.76 (2H, br d, J = 8.7 Hz, H-3′, 5′), 6.90 (1H, d, J = 12.8 Hz, H-7′), 7.68 (2H, br d, J = 8.4 Hz, H-2′, 6′). ^{13}C-NMR (150 MHz, CD_3OD): δ_C 36.8 (C-2), 37.8 (C-6), 64.7 (C-4), 70.0 (C-3), 73.0 (C-1), 77.7 (C-5), 115.8 (C-3′, 5′), 116.0 (C-8′), 127.5 (C-1′), 134.0 (C-2′, 6′), 146.1 (C-7′), 160.3 (C-4′), 166.9 (C-9′), 178.9 (C-7).

*4-O-(E)-p-coumaroylquinic acid (**9**)*, colorless oil, $C_{16}H_{18}O_8$. ESI-MS (negative) *m/z* 337.0931 $[M-H]^-$ (calcd. for 337.0923). UV (MeOH) λ_{max} 228, 312 nm. ^{1}H-NMR (600 MHz, CD_3OD): δ_H 2.04 (1H, m, H-6eq), 2.05 (1H, m, H-2eq), 2.16 (1H, m, H-2ax), 2.18 (1H, m, H-6ax), 4.28 (1H, m, H-5), 4.30 (1H, m, H-3), 4.81 (1H, m, H-4), 6.45 (1H, d, J = 16.0 Hz, J H-8′), 6.83 (2H, d, = 8.5 Hz, H-3′, 5′), 7.50 (2H, d, J = 8.4 Hz, H-2′, 6′), 7.72 (1H, d, J = 15.9 Hz, H-7′). ^{13}C-NMR (150 MHz, CD_3OD):

δ_C 38.7 (C-2), 42.4 (C-6), 65.9 (C-5), 69.5 (C-3), 77.0 (C-1), 79.2 (C-4), 115.5 (C-8′), 116.8 (C-3′, 5′), 127.3 (C-1′), 131.2 (C-2′, 6′), 146.7 (C-7′), 161.2 (C-4′), 169.0 (C-9′), 178.5 (C-7).

*4-O-(Z)-p-coumaroylquinic acid (**10**)*, colorless oil, $C_{16}H_{18}O_8$. ESI-MS (negative) *m/z* 337.0928 $[M-H]^-$ (calcd. for 337.0923). UV (MeOH) λ_{max} 308 nm. ^{1}H-NMR (600 MHz, CD_3OD): δ_H 2.00 (1H, dd, *J* = 10.8, 13.3 Hz, H-6ax), 2.05 (1H, m, H-2ax), 2.16 (1H, dd, *J* = 2.9, 14.5 Hz, H-2eq), 2.18 (1H, m, H-6eq), 4.24 (1H, m, H-3), 4.29 (1H, dd, *J* = 3.2, 7.0 Hz, H-5), 4.77 (1H, dd, *J* = 3.0 Hz, 9.3 Hz H-4), 5.91 (1H, d, *J* = 12.8 Hz, H-8′), 6.75 (2H, d, *J* = 8.7 Hz, H-3′, 5′), 6.88 (1H, d, *J* = 12.8 Hz, H-7′), 7.70 (2H, d, *J* = 8.7 Hz, H-2′, 6′). ^{13}C-NMR (150 MHz, CD_3OD): δ_C 38.4 (C-2), 42.7 (C-6), 65.5 (C-5), 69.6 (C-3), 76.6 (C-1), 79.0 (C-4), 115.8 (C-3′, 5′), 116.7 (C-8′), 127.6 (C-1′), 133.9 (C-2′, 6′), 145.3 (C-7′), 160.1 (C-4′), 167.8 (C-9′), 177.3 (C-7).

*5-O-(E)-p-coumaroylquinic acid (**11**)*, colorless oil, $C_{16}H_{18}O_8$. HR-ESI-MS (negative) *m/z* 337.0928 $[M-H]^-$ (calcd. for 337.0923). UV (MeOH) λ_{max} 228, 312 nm. ^{1}H-NMR (600 MHz, CD_3OD): δ_H 1.95-2.22 (4H, m, H-2, 6), 3.72 (1H, dd, *J* = 2.2, 8.3 Hz, H-4), 4.17 (1H, brs, H-3), 5.35 (1H, m, H-5), 6.32 (1H, d, *J* = 16.0 Hz, H-8′), 6.80 (2H, d, *J* = 8.3 Hz, H-3′, 5′), 7.46 (2H, d, *J* = 8.6 Hz, H-2′, 6′), 7.62(1H, d, *J* = 16.0 Hz, H-7′). ^{13}C-NMR (150 MHz, CD_3OD): δ_C 38.3 (C-2), 39.2 (C-6), 71.8 (C-4), 72.1 (C-3), 73.8 (C-5), 76.8 (C-1), 115.4 (C-8′), 116.8 (C-3′, 5′), 127.2 (C-1′), 131.2 (C-2′, 6′), 146.6 (C-7′), 161.3 (C-4′), 168.8 (C-9′).

*Neochlorogenic acid (**12**)*, amorphous white powder, $C_{16}H_{18}O_9$. ESI-MS (negative) *m/z* 353.17 $[M-H]^-$. UV (MeOH) λ_{max} 218, 240sh, 300sh, 326. ^{1}H-NMR (600 MHz, CD_3OD): δ_H 1.94-2.14 (4H, m, H-2ax, H-2eq, H-6ax, H-6eq), 3.69 (1H, m, H-4), 4.10 (1H, m, H-3), 5.37 (1H, m, H-5), 6.30 (1H, d, J =15.8 Hz, H-7′), 6.77 (1H, d, J =8.0 Hz, H-5′), 6.94 (1H, d, J =7.7 Hz, H-6′), 7.05 (1H, s, H-2′), 7.56 (1H, d, J = 15.8 Hz, H-8′). ^{13}C-NMR (150 MHz, CD_3OD): 36.9 (C-2), 40.8 (C-6), 68.8 (C-3), 72.8 (C-4), 74.3 (C-5), 75.7 (C-1), 115.1 (C-8’), 115.8 (C-2’), 116.5 (C-5′), 122.9 (C-6′), 128.0 (C-1′), 146.8 (C-7′), 146.8 (C-3′), 149.4 (C-4′), 168.9 (C-9′), 179.4 (COO-).

*Crypto-chlorogenic acid (**13**)*, amorphous white powder, $C_{16}H_{18}O_9$. ESI-MS (negative) *m/z* 353.0875 $[M-H]^-$ (calcd. for 353.0873). UV (MeOH) λ_{max} 218, 240, 302sh, 328 nm. ^{1}H-NMR (600 MHz, CD_3OD): δ_H 1.90-2.30 (4H, m, H-2ax, H-2eq, H-6ax, H-6eq), 4.29 (1H, m, H-5), 4.30 (1H, m, H-3), 4.80 (1H, dd, *J* = 2.8, 9.3 Hz, H-4), 6.36 (1H, d, *J* = 15.9 Hz, H-8′), 6.78 (1H, d, *J* = 8.2 Hz, H-5′), 6.96 (1H, dd, *J* =1.6, 8.2 Hz, H-6′), 7.06 (1H, d, *J* = 1.6 Hz, H-2′), 7.64 (1H, d, *J* = 15.9 Hz, H-7′). ^{13}C-NMR (150 MHz, CD_3OD): δ_C 38.4 (C-2), 42.7 (C-6), 65.6 (C-5), 69.6 (C-3), 76.7 (C-1), 79.3 (C-4), 115.2 (C-8′), 115.3 (C-2′), 116.5 (C-5′), 123.0 (C-6′), 127.8 (C-1′), 146.8 (C-3′), 147.1 (C-7′),

149.5 (C-4′), 169.0 (C-9′), 177.5(COO-).

*chlorogenic acid (**14**)*, amorphous white powder, $C_{16}H_{18}O_9$. ESI-MS (negative) *m/z* 353.0883 $[M-H]^-$ (calcd. for $C_{16}H_{17}O_9$, 353.0873). UV (MeOH) λ_{max} 218, 240sh, 300sh, 326 nm. ^{1}H-NMR (600 MHz, CD_3OD): δ_H 2.00-2.30 (4H, m, H-2ax, H-2eq, H-6ax, H-6eq), 3.73 (1H, dd, *J* = 3.0, 8.5 Hz, H-4), 4.17 (1H, m, H-3), 5.34 (1H, m, H-5), 6.26 (1H, d, *J* = 15.9 Hz, H-8′), 6.78 (1H, d, *J* = 8.2 Hz, H-5′), 6.95 (1H, dd, *J* = 1.9, 8.2 Hz, H-6′), 7.05 (1H, d, *J* = 1.9 Hz, H-2′), 7.56 (1H, d, *J* = 15.9 Hz, H-7′). ^{13}C-NMR (150 MHz, CD_3OD): δ_C 38.2 (C-2), 38.8 (C-6), 71.3 (C-3), 72.0 (C-5), 73.5 (C-4), 76.2 (C-1), 115.2 (C-2′), 115.2 (C-8′), 116.5 (C-5′), 123.0 (C-6′), 127.8 (C-1′), 146.8 (C-3′), 147.1 (C-7′), 149.5 (C-4′), 168.7 (C-9′), 177.1 (C-7).

*3-O-(E)-feruloylquinic acid (**15**),* amorphous white powder, $C_{17}H_{20}O_9$. ESI-MS (negative) *m/z* 367.25 $[M-H]^-$. UV (MeOH) λ_{max} 218, 236, 324 nm. ^{1}H-NMR (600 MHz, CD_3OD): δ_H 1.95-2.18 (4H, m, H-2ax, H-2eq, H-6ax, H-6eq), 3.79 (1H, m, H-4), 3.88 (3H, s, H-8), 4.01 (1H, m, H-3), 5.42 (1H, m, H-5), 6.38 (1H, d, *J* = 15.8 Hz, H-8′), 6.80 (1H, d, *J* = 8.0 Hz, H-5′), 7.05 (1H, d, *J* = 8.0 Hz, H-6′), 7.17 (1H, s, H-2′), 7.65 (1H, d, *J* = 15.8 Hz, H-7′). ^{13}C-NMR (150 MHz, CD_3OD): δ_C 37.1 (C-2), 37.2 (C-6), 56.4 ($-OCH_3$), 69.9 (C-3), 72.4 (C-5), 73.4 (C-4), 76.6 (C-1), 111.8 (C-2′), 116.0 (C-8′), 116.5 (C-5′), 124.0 (C-6′), 127.9 (C-1′), 146.8 (C-3′), 149.3 (C-7′), 150.5 (C-4′), 168.7 (C-9′).

*Quercetin (**16**)*, amorphous yellow powder, $C_{15}H_{10}O_7$. ESI-MS (negative) *m/z* 301.0358 $[M-H]^-$ (calcd. for 301.0348). UV (MeOH) λ_{max} 256, 372 nm. ^{1}H-NMR (600 MHz, DMSO-d_6): δ_H 6.19 (1H, s, H-6), 6.41 (1H, s, H-8), 6.88 (1H, d, *J* = 8.5, H-5′), 7.54 (1H, dd, *J* = 8.4, 2.0, H-6′), 7.68 (1H, d, *J* = 2.0, H-2′), 12.50 (1H, s, 5-OH). ^{13}C-NMR (150 MHz, DMSO-d_6): δ_C 93.4 (C-8), 98.2 (C-6), 103.0 (C-10), 115.0 (C-2′),115.6 (C-5′), 120.0 (C-6′), 122.0 (C-1′), 135.8 (C-3), 145.1 (C-3′), 146.8 (C-2), 147.7 (C-4′), 156.2 (C-9), 160.7 (C-5), 164.0 (C-7), 175.9 (C-4).

*Quercetin-3-O-α-L-arabinopyranoside (**17**)*, amorphous yellow powder, $C_{20}H_{18}O_{11}$. ESI-MS (negative) *m/z* 433.0770 $[M-H]^-$ (calcd. for 433.0771). UV (MeOH) λ_{max} 256, 356 nm. ^{1}H-NMR (600 MHz, CD_3OD): δ_H 3.45 (1H, dd, J=2.7,13.4 Hz, H-5), 3.66 (1H, dd, J=3.0, 8.5 Hz, H-3″), 3.82 (1H, m, H-5″), 3.84 (1H, m, H-4″), 3.91 (1H, dd, *J*=6.7, 8.3 Hz, H-2″), 5.13 (1H, d, *J*=6.6 Hz, H-1″), 6.23 (1H, d, *J*=2.0 Hz, H-6), 6.43 (1H, brs, H-8), 6.90 (1H, d, *J*=8.5 Hz, H-5′), 7.58 (1H, dd, *J*=2.0, 8.5 Hz, H-6′), 7.75 (1H, d, *J*=2.0 Hz, H-2′). ^{13}C-NMR (150 MHz, CD_3OD): δ_C 67.0 (C-5″), 69.1 (C-4″), 72.8 (C-2″), 74.0 (C-3″), 94.8 (C-8), 100.0 (C-6), 104.5 (C-1″), 105.6 (C-10), 116.3 (C-5′), 117.5 (C-2′), 122.8 (C-1′), 123.1 (C-6′), 135.5 (C-3), 145.9 (C-3′), 149.9 (C-4′), 158.4 (C-2), 158.8 (C-9), 162.8 (C-5), 166.0 (C-7), 179.5 (C-4).

Quercetin-3-O-β-D-galactoside ***(18)***, amorphous yellow powder, $C_{21}H_{20}O_{12}$. HR-ESI-MS (positive) *m/z* 487.0827 $[M+Na]^+$ (calcd. for 487.0852). UV (MeOH) λ_{max} 256, 354 nm. ^{1}H-NMR (600 MHz, CD_3OD): δ_H 3.47 (1H, t, *J* = 6.1 Hz, H-5″), 3.55 (2H, m, H-3″, 6″b), 3.64 (1H, dd, *J* = 6.0, 11.2 Hz, 6″a), 3.82 (1H, dd, *J* = 8.0, 9.5 Hz, H-2″), 3.85 (1H, d, *J* = 3.1 Hz, H-4″), 5.17 (1H, d, *J* = 7.8 Hz, H-1″), 6.20 (1H, d, *J* = 1.8 Hz, H-6), 6.40 (1H, d, *J* = 1.8 Hz, H-8), 6.86 (1H, d, *J* = 8.5 Hz, H-5′), 7.59 (H, dd, *J* = 8.5, 2.0 Hz, H-6′), 7.84 (1H, d, *J* = 2.0 Hz, H-2′). ^{13}C-NMR (150 MHz, CD_3OD): δ_C 61.9 (C-6″), 70.0 (C-4″), 73.2 (C-2″), 75.1 (C-3″), 77.2 (C-5″), 94.7 (C-8), 99.9 (C-6), 105.4 (C-1″), 105.6 (C-10), 116.1 (C-2′), 117.8 (C-5′), 122.9 (C-1′, 6′), 135.8 (C-3), 145.8 (C-4′), 150.0 (C-3′), 158.5 (C-2), 158.8 (C-9), 163.0 (C-5), 166.1 (C-7), 179.5 (C-4).

Quercetin-3-O-β-D-glucoside ***(19)***, amorphous yellow powder, $C_{21}H_{20}O_{12}$. HR-ESI-MS (positive) *m/z* 487.0837 $[M+Na]^+$ (calcd. for 487.0852). UV (MeOH) λ_{max} 256, 354 nm.^{1}H-NMR (600 MHz, CD_3OD): δ_H 3.22 (1H, ddd, *J* = 2.1, 5.3, 9.4 Hz, H-5″), 3.34 (1H, t, *J* = 9.9 Hz, H-4″), 3.42 (1H, t, *J* = 8.9 Hz, H-3″), 3.47(1H, dd, *J* = 7.9, 8.9Hz, H-2″), 3.57 (1H, dd, *J* = 5.3, 11.9 Hz, H-6″b), 3.71(1H, dd, *J* = 2.1, 11.9 Hz, H-6″a), 5.25 (1H, d, *J* = 7.7 Hz, H-1″), 6.20 (1H, s, H-6), 6.39 (1H, s, H-8), 6.86 (1H, d, *J* = 8.4 Hz, H-5′), 7.59 (H, dd, *J* = 8.4, 1.9 Hz, H-6′), 7.71 (1H, d, *J* = 1.9 Hz, H-2′). ^{13}C-NMR (150 MHz, CD_3OD): δ_C 62.5 (C-6″), 71.2 (C-4″), 75.7 (C-2″), 78.1 (C-3″), 78.4 (C-5″), 94.7 (C-8), 99.9 (C-6), 104.3 (C-1″), 105.7 (C-10), 116.0 (C-2′), 117.5 (C-5′), 123.1 (C-6′), 123.2 (C-1′), 135.6 (C-3), 145.9 (C-4′), 149.9 (C-3′), 158.5 (C-2), 159.0 (C-9), 163.1 (C-5), 166.1 (C-7), 179.5 (C-4).

Quercetin-3-O-rutinoside ***(20)***, amorphous yellow powder, $C_{27}H_{30}O_{16}$. ESI-MS (negative) *m/z* 609.1465 $[M-H]^-$ (calcd. for 609.1456). UV (MeOH) λ_{max} 256, 354 nm. ^{1}H-NMR (600 MHz, CD_3OD): δ_H 1.12 (3H, d, *J* = 6.2 Hz, H-6‴ CH_3), 3.27 (1H, m, H-4″), 3.29 (1H, m, H-4‴), 3.34 (1H, m, H-5″), 3.39 (1H, m, H-6″b), 3.40 (1H, m, H-3″), 3.45 (1H, m, H-5‴), 3.48 (1H, m, H-2″), 3.55 (1H, dd, *J* =3.4, 9.5 Hz, H-3‴), 3.65 (1H, dd, *J* = 1.2, 2.9 Hz, H-2‴), 3.81 (1H, d, *J* = 11 Hz, H-6″a), 4.51 (1H, s, H-1‴), 5.10 (1H, d, *J* = 7.6 Hz, H-1″), 6.20 (1H, s, H-6), 6.38 (1H, s, H-8), 6.87 (1H, d, *J* = 8.4 Hz, H-5′), 7.62 (1H, dd, *J* = 2.0, 8.4 Hz, H-6′), 7.67 (1H, d, *J* = 2.0 Hz, H-2′). ^{13}C-NMR (150 MHz, CD_3OD): δ_C 17.9 (C-6‴), 68.5 (C-6″), 69.7 (C-5‴), 71.3 (C-4″), 72.1 (C-2‴), 72.2 (C-3‴), 73.9 (C-4‴), 75.7 (C-2″), 77.2 (C-5″), 78.1 (C-3″), 94.9 (C-8), 99.9 (C-6), 102.4 (C-1‴), 104.7 (C-1″), 105.6 (C-10), 116.0 (C-2′), 117.7 (C-5′), 123.1 (C-1′), 123.6 (C-6′), 135.6 (C-3), 145.8 (C-4′), 149.8 (C-3′), 158.5 (C-2), 159.3 (C-9), 162.9 (C-5), 166.0 (C-7), 179.3 (C-4).

Quercetin-3-O-α-L-rhamnopyranosyl-(1→6)-β-D-galactpyranoside ***(21)***, amorphous yellow

powder, $C_{27}H_{30}O_{16}$. ESI-MS (negative) *m/z* 609.44 $[M-H]^-$. UV (MeOH) λ_{max} 256, 354 nm. ^{1}H-NMR (600 MHz, CD_3OD): δ_H 1.19 (3H, d, *J* = 6.2 Hz, H-6'''), 3.29 (1H, m, H-4'''), 3.41 (1H, m, H-6'' b), 3.49 (1H, m, H-3'''), 3.53 (1H, dd, *J* = 9.5, 3.4 Hz, H-5'''), 3.56 (1H, m, H-3''), 3.58 (1H, m, H-2'''), 3.65 (1H, m, H-5''), 3.74 (1H, dd, *J* = 10.1, 5.7 Hz, H-6''a), 3.79 (1H, m, H-4''), 3.82 (1H, m, H-2''), 4.53 (1H, s, H-1'''), 5.07 (1H, d, *J* = 7.8 Hz, H-1''), 6.21 (1H, d, *J* = 1.8 Hz, H-6), 6.40 (1H, d, *J* = 1.8 Hz, H-8), 6.87 (1H, d, *J* = 8.4 Hz, H-5'), 7.59 (1H, dd, *J* = 8.4, 1.8 Hz, H-6'), 7.87 (1H, d, *J* = 1.9 Hz, H-2'). ^{13}C-NMR (150 MHz, CD_3OD): δ_C 18.0 (C-6'''), 67.3 (C-6''), 69.7 (C-5'''), 70.2 (C-4''), 72.0 (C-2'''), 72.3 (C-3'''), 73.1 (C-2''), 73.9 (C-4'''), 75.1 (C-3''), 75.3 (C-5''), 94.8 (C-8), 100.0 (C-6), 101.9 (C-1'''), 105.5 (C-10), 106.0 (C-1''), 116.1 (C-5'), 117.9 (C-2'), 122.8 (C-1'), 123.0 (C-6'), 135.9 (C-3), 145.7 (C-3'), 150.0 (C-4'), 158.5 (C-2), 159.0 (C-9), 162.9 (C-5), 166.2 (C-7), 179.5 (C-4).

Quercetin-3-O-α-L-rhamnopyranosyl-(1→2)-[α-L-rhamnopyranosyl-(1→6)-β-D-glucopyranoside] ***(22)***, amorphous yellow powder, $C_{33}H_{40}O_{20}$. ESI-MS (negative) *m/z* 755.32 $[M-H]^-$. UV (MeOH) λ_{max} 256, 354 nm. ^{1}H-NMR (600 MHz, CD_3OD): δ_H 1.01 (3H, d, *J* = 6.2 Hz, H-6'''), 1.08 (3H, d, *J* = 6.2 Hz, H-6''''), 3.24 (1H, t, *J* = 9.5 Hz, H-4''''), 3.28 (1H, t, *J* = 9.5 Hz, H-4'''), 3.34 (1H, m, H-5''), 3.35 (1H, m, H-4''), 3.40 (1H, m, H-6b''), 3.43 (1H, m, H-5''''), 3.49 (1H, dd, *J* = 9.5, 3.3 Hz, H-3''''), 3.54 (1H, t, *J* = 8.9 Hz, H-3''), 3.59 (1H, brs, H-2''''), 3.64 (1H, dd, *J* = 8.9, 7.9 Hz, H-2''), 3.80 (1H, m, H-3'''), 3.82 (1H, m, H-6a''), 4.00 (1H, brs, H-2'''), 4.08 (1H, dd, *J* = 9.3, 6.2 Hz, H-5'''), 4.51 (1H, brs, H-1''''), 5.22 (1H, brs, H-1'''), 5.59 (1H, d, *J* = 7.8 Hz, H-1''), 6.18 (1H, d, *J* = 1.8 Hz, H-6), 6.37(1H, d, *J* = 1.8 Hz, H-8), 6.87 (1H, d, *J* = 8.2 Hz, H-5'), 7.59 (1H, dd, *J* = 8.2, 2.0 Hz, H-6'), 7.61 (1H, d, *J* = 2.0 Hz, H-2'). ^{13}C-NMR (150 MHz, CD_3OD): δ_C 17.5 (C-6'''), 17.9 (C-6''''), 68.3 (C-6''), 69.8 (C-5''''), 70.0 (C-5'''), 71.9 (C-4''), 72.2 (C-2''''), 72.3 (C-3'''), 72.4 (C-2'''), 73.9 (C-4''''), 74.1 (C-4'''), 77.1 (C-5''), 78.9 (C-3''), 80.1 (C-2''), 94.7 (C-8), 99.8 (C-6), 100.5 (C-1''), 102.3 (C-1''''), 102.7 (C-1'''), 105.9 (C-10), 116.1 (C-5'), 117.4 (C-2'), 123.5 (C-1'), 123.6 (C-6'), 134.5 (C-3), 146.0 (C-3'), 149.6 (C-4'), 158.5 (C-9), 159.0 (C-2), 163.1 (C-5), 165.7 (C-7), 179.3 (C-4).

Quercetin-3-O-α-L-rhamnopyranosyl-(1→2)-[α-L-rhamnopyranosyl-(1→6)-β-D-galactpyranoside] ***(23)***, amorphous yellow powder, $C_{33}H_{40}O_{20}$. ESI-MS (negative) *m/z* 755.35 $[M-H]^-$. UV (MeOH) λ_{max} 256, 354 nm.^{1}H-NMR (600 MHz, CD_3OD): 0.95 (3H, d, *J* = 6.2 Hz, H-6'''), 1.18 (3H, d, *J* = 6.3 Hz, H-6''''), 3.22-4.10 (14H, sugar-H), 4.54 (1H, d, *J* = 1.2 Hz, H-1''''), 5.21 (1H, brs, H-1'''), 5.66 (1H, d, *J* = 7.8 Hz, H-1''), 6.18 (1H, brs, H-6), 6.37 (1H, brs, H-8), 6.87 (1H, d, *J* = 8.5 Hz, H-5'), 7.56 (1H, dd, *J* = 2.0, 8.5 Hz, H-6'), 7.69 (1H, d, *J* = 2.0 Hz, H-2').^{13}C-NMR (150 MHz, CD_3OD): δ_C 17.4 (C-6'''), 18.0 (C-6''''), 67.0 (C-6''), 69.7 (C-5''''), 69.8 (C-5'''), 70.9 (C-4''), 72.1 (C-

2''''),72.3 (C-3''''), 72.3 (C-3'''),72.4 (C-2'''), 73.9 (C-4''''), 74.1 (C-4'''), 75.3 (C-5''), 75.7 (C-3''), 77.5 (C-2''), 94.6 (C-8), 99.8 (C-6), 101.0 (C-1''), 101.9 (C-1''''), 102.6 (C-1'''), 105.8 (C-10), 116.1 (C-5'), 117.3 (C-2'), 123.0 (C-6'), 123.3 (C-1'), 134.5 (C-3), 145.9 (C-3'), 149.6 (C-4'), 158.4 (C-9), 158.4 (C-2), 163.1 (C-5), 165.8 (C-7), 179.3 (C-4).

*Isorhamnetin (**24**)*, amorphous yellow powder, $C_{16}H_{12}O_7$. HR-ESI-MS (negative) *m/z* 315.0515 $[M-H]^-$ (calcd. for 315.0505). UV (MeOH) λ_{max} 254, 372 nm. ^{1}H-NMR (600 MHz, CD_3OD): δ_H 3.94 (3H, s, –OCH_3), 6.19 (1H, d, *J* = 2.0 Hz, H-6), 6.43 (1H, d, *J* = 2.0 Hz, H-8), 6.93 (1H, d, *J* = 8.5 Hz, H-5′), 7.73 (1H, dd, *J* = 8.4, 2.0 Hz, H-6′), 7.86 (1H, d, *J* = 2.0 Hz, H-2′). ^{13}C-NMR (150 MHz, DMSO-d_6): δ_C 55.8 (–OCH_3), 93.7 (C-8), 98.3 (C-6), 103.1 (C-10), 111.7 (C-2′), 115.6 (C-5′), 121.8 (C-6′), 122.0 (C-1′), 135.9 (C-3), 146.7 (C-4′), 147.5 (C-2), 148.9 (C-3′), 156.3 (C-9), 160.8 (C-5), 164.1 (C-7), 176.0 (C-4).

*Isorhamnetin-3-O-β-D-galactopyranoside (**25**)*, amorphous yellow powder, $C_{22}H_{22}O_{12}$. ESI-MS (negative) *m/z* 477.23 $[M-H]^-$. UV (MeOH) λ_{max} 256, 352 nm. ^{1}H-NMR (600 MHz, CD_3OD): δ_H 3.47 (1H, t, *J* = 6.3 Hz, H-5″), 3.56 (2H, m, H-3″, 6″a), 3.65 (1H, dd, *J* = 5.8, 11.2 Hz, H-6″b), 3.81 (1H, dd, *J* = 7.8, 9.7 Hz, H-2″), 3.84 (1H, d, *J* = 2.8 Hz, H-4″), 3.96 (3H, s, 3'-OCH_3), 5.33 (1H, d, *J* = 7.7 Hz, Gal H-1), 6.21 (1H, d, *J* = 2.0 Hz, H-6), 6.41 (1H, d, *J* = 2.0 Hz, H-8), 6.90 (1H, d, *J* = 8.5 Hz, H-5′), 7.58 (H, dd, *J* = 8.5, 2.0 Hz, H-6′), 8.03 (1H, d, *J* = 2.0 Hz, H-2′). ^{13}C-NMR (150 MHz, CD_3OD): δ_C 57.0 (-OCH_3), 62.2 (C-6″), 70.0 (C-4″), 73.2 (C-2″), 75.0 (C-3″), 77.3 (C-5″), 94.7 (C-8), 99.9 (C-6), 104.4 (C-1″), 105.8 (C-10), 114.5 (C-2′), 115.9 (C-5′), 123.0 (C-1′), 123.6 (C-6′), 135.4 (C-3), 148.4 (C-3′), 150.8 (C-4′), 158.5 (C-2), 158.6 (C-9), 163.1 (C-5), 166.0 (C-7), 179.5 (C-4).

*Isorhamnetin-3-O-β-D-glucopyranoside (**26**)*, amorphous yellow powder, $C_{22}H_{22}O_{12}$. ESI-MS (negative) *m/z* 477.1054 $[M-H]^-$ (calcd. for 477.1033). UV (MeOH) λ_{max} 254, 354 nm. ^{1}H-NMR (600 MHz, CD_3OD): δ_H 3.24 (1H, m, H-5″), 3.33 (1H, brs, H-4″), 3.44 (1H, m, H-3″), 3.46 (1H, m, H-2″), 3.56 (1H, dd, *J* = 5.5, 11.9 Hz, H-6″b), 3.74 (1H, dd, *J* = 2.0, 11.9 Hz, H-6″a), 3.94 (3H, s, 3'-OCH_3), 5.40 (1H, d, *J* = 7.3 Hz, H-1″), 6.19 (1H, s, H-6), 6.38 (1H, s, H-8), 6.89 (1H, d, *J* = 8.5 Hz, H-5′), 7.57 (1H, dd, *J* = 8.5, 1.0 Hz, H-6′), 7.92 (1H, d, *J* = 1.0 Hz, H-2′). ^{13}C-NMR (150 MHz, CD_3OD): δ_C 56.8 (-OCH_3), 62.5 (C-6″), 71.5 (C-4″), 75.9 (C-2″), 78.0 (C-5″), 78.5 (C-3″), 94.7 (C-8), 99.9 (C-6), 103.6 (C-1″), 105.8 (C-10), 114.3 (C-2′), 116.0 (C-5′), 123.1 (C-1′), 123.8 (C-6′), 135.3 (C-3), 148.4 (C-4′), 150.8 (C-3′), 158.4 (C-2), 158.6 (C-9), 163.1 (C-5), 166.0 (C-7), 179.4 (C-4).

*Isorhamnetin-3-O-[α-L-rhamnopyranosyl-(1→6)-β-D-glucopyranoside (**27**)*, amorphous yellow powder, $C_{28}H_{32}O_{16}$. HR-ESI-MS (positive) *m/z* 647.1564 $[M+Na]^+$ (calcd. for 647.1588). UV (MeOH)

λ_{max} 254, 356 nm. ^{1}H-NMR (600 MHz, CD_3OD): δ_H 1.10 (3H, d, *J* = 6.2 Hz, H-6'''), 3.22-3.52(8H, m, H-2'', 3'', 4'', 5'', 6b''), 3.63 (1H, brs, H-2'''), 3.81 (1H, d, *J* = 11.0, H-6a″), 3.94 (3H, s, 3'-OCH_3), 4.53 (1H, brs, H-1'''), 5.23 (1H, d, *J* = 6.8 Hz, H-1''), 6.19 (1H, s, H-6), 6.38 (1H, s, H-8), 6.90 (1H, d, *J* = 7.4 Hz, H-5'), 7.60 (1H, d, *J* = 7.4Hz, H-6'), 7.94 (1H, s, H-2'). ^{13}C-NMR (150 MHz, CD_3OD): δ_C 17.9 (C-6'''), 56.7 (-OCH_3), 68.5 (C-6''), 69.7 (C-5'''), 71.6 (C-4''), 72.0 (C-3'''), 72.2 (C-2'''), 73.8 (C-4'''), 75.9 (C-2''), 77.3 (C-5''), 78.1 (C-3''), 94.9 (C-8), 99.9 (C-6), 102.5 (C-1'''), 104.4 (C-1''), 105.6 (C-10), 114.5 (C-2'), 116.0 (C-5'), 122.9 (C-6'), 123.9 (C-1'), 135.4 (C-3), 148.2 (C-3'), 150.8 (C-4'), 158.4 (C-2), 158.8 (C-9), 162.9 (C-5), 166.0 (C-7), 179.2 (C-4).

*Isorhamnetin-3-O-α-L-rhamnopyranosyl-(1→2)-[α-L-rhamnopyranosyl-(1→6)-β-D-galactopyranoside] (**28**)*, amorphous yellow powder, $C_{34}H_{42}O_{20}$. ESI-MS (positive) *m/z* 793.2132 $[M+Na]^+$ (calcd. for 793.2167). UV (MeOH) λ_{max} 254, 354 nm. ^{1}H-NMR (600 MHz, CD_3OD): δ_H 0.88 (3H, d, *J* = 6.2 Hz, H-6'''), 1.17 (1H, d, *J* = 6.3 Hz, H-6''''), 3.00-4.10 (14H, m, H-2'', H-3'', H-4'', H-5'', H-6''a, H-6''b, H-2''', H-3''', H-4''', H-5''',H-2'''', H-3'''', H-4'''', H-5''''), 4.00 (3H, s, 3'-OCH_3), 4.56 (1H, d, *J* = 1.5 Hz, H-1''''), 5.16 (1H, s, H-1'''), 5.79 (1H, d, *J* = 7.8 Hz, H-1''), 6.18 (1H, d, *J* = 1.6 Hz, H-6), 6.38 (1H, brs, H-8), 6.91 (1H, d, *J* = 8.4 Hz, H-5'), 7.52 (1H, dd, *J* =1.8, 8.4 Hz, H-6'), 8.07 (1H, d, *J* = 1.8 Hz, H-2'). ^{13}C-NMR (150 MHz, CD_3OD): δ_C 17.4 (C-6'''), 18.0 (C-6''''), 57.2 (-OCH_3), 67.1 (C-6''), 69.7 (C-5''''), 69.8 (C-5'''), 70.5 (C-4''), 72.1 (C-2''''), 72.3 (C-3''''), 72.4 (C-3'''), 72.4 (C-2'''), 73.8 (C-4''''), 74.0 (C-4'''), 75.4 (C-5''), 75.6 (C-3''), 77.8 (C-2''), 94.6 (C-8), 99.7 (C-6), 100.8 (C-1''), 101.8 (C-1''''), 102.8 (C-1'''), 105.9 (C-10), 114.6 (C-2'), 115.9 (C-5'), 123.2 (C-6'), 123.3 (C-1'), 134.3 (C-3), 148.4 (C-3'), 150.5 (C-4'), 158.4 (C-2), 158.4 (C-9), 163.3(C-5), 165.7 (C-7), 179.3 (C-4).

*Isorhamnetin-3-O-α-L-rhamnopyranosyl-(1→2)-[α-L-rhamnopyranosyl-(1→6)-β-D-glucopyranoside] (**29**)*, amorphous yellow powder, $C_{34}H_{42}O_{20}$. ESI-MS (negative) *m/z* 769.2219 $[M-H]^-$ (calcd. for 769.2191). UV (MeOH) λ_{max} 254, 354 nm. ^{1}H-NMR (600 MHz, CD_3OD): δ_H 0.92 (3H, d, *J* = 6.2 Hz, H-6'''), 1.07 (3H, d, *J* = 6.3 Hz, H-6''''), 3.32 (1H, brs, H-4'''), 3.24 (1H, t, *J* = 9.4 Hz, H-4''''), 3.25 (1H, m, H-4''), 3.39 (1H, m, H-5''), 3.40 (1H, m, H-5''''), 3.45 (1H, m, H-6''b), 3.52 (1H, m, H-3''''), 3.58 (1H, m, H-3''), 3.59 (1H, m, H-2''''), 3.64 (1H, m, H-2''), 3.78 (1H, m, H-3'''), 3.83 (1H, m, H-6''a), 3.97 (3H, s, -OCH_3), 4.00 (1H, brs, H-2'''), 4.04 (1H, dd, *J* = 6.2, 9.7 Hz, H-5'''), 4.54 (1H, d, *J* = 1.5 Hz, H-1''''), 5.19 (1H, d, *J* = 1.0 Hz, H-1'''), 5.73 (1H, d, *J* = 7.7 Hz, H-1''), 6.19 (1H, d, *J* = 1.8 Hz, H-6), 6.39 (1H, d, *J* = 1.8 Hz, H-8), 6.92 (1H, d, *J* = 8.5 Hz, H-5'), 7.58 (1H, dd, *J* = 2.0, 8.4 Hz, H-6'), 7.94 (1H, d, *J* = 2.0 Hz, H-2'). ^{13}C-NMR (150 MHz, CD_3OD): δ_C 17.4 (C-6'''), 17.8

(C-6''''), 57.0 (3'-OCH_3), 68.2 (C-6''), 69.8 (C-5''''), 69.9 (C-5'''), 72.0 (C-4''), 72.1 (C-2''''), 72.3 (C-3''''), 72.3 (C-3'''), 72.4 (C-2'''), 73.8 (C-4''''), 73.9 (C-4'''), 77.2 (C-5''), 78.8 (C-3''), 80.1 (C-2''), 94.7 (C-8), 99.8 (C-6), 100.5 (C-1''), 102.4 (C-1''''), 102.7 (C-1'''), 105.9 (C-10), 114.5 (C-2'), 116.1 (C-5'), 123.3 (C-1'), 123.7 (C-6'), 134.3 (C-3), 148.4 (C-3'), 150.6 (C-4'), 158.4 (C-9), 158.5 (C-2), 163.2 (C-5), 165.7 (C-7), 179.2 (C-4).

*Kaempferol (**30**)*, amorphous yellow powder, $C_{15}H_{10}O_6$. ESI-MS (negative) *m/z* 285.0404 [M-H]$^-$ (calcd. for 285.0399). UV (MeOH) λ_{max} 266, 366 nm. ^{1}H-NMR (600 MHz, CD_3OD): δ_H 6.18 (1H, s, H-6), 6.40 (1H, s, H-8), 6.90 (2H, d, J = 8.5 Hz, H-3', 5'), 8.09 (2H, d, J = 8.5 Hz, H-2', 6'). ^{13}C-NMR (150 MHz, CD_3OD): δ_C 94.4 (C-8), 99.3 (C-6), 104.5 (C-10), 116.3 (C-3', 5'), 123.8 (C-1'), 130.7 (C-2', 6'), 137.4 (C-3), 148.1 (C-2), 158.3 (C-9), 160.5 (C-4'), 162.5 (C-5), 165.6 (C-7), 177.5 (C-4).

*Kaempferol-3-O-α-L-arabinoside (**31**)*, amorphous yellow powder, $C_{20}H_{18}O_{10}$. HR-ESI-MS (negative) *m/z* 417.0821 [M-H]$^-$ (calcd. for 417.0822). UV (MeOH) λ_{max} 266, 366 nm. ^{1}H-NMR (600 MHz, CD_3OD): δ_H 3.40 (1H, d, J = 11.0 Hz, H-5″a), 3.63 (1H, dd, J = 2.3, 8.1 Hz, H-3″), 3.78 (1H, m, H-5″b), 3.79 (1H, brs, H-4″), 3.89 (1H, dd, J = 6.7, 8.0 Hz, H-2″), 5.13 (1H, d, J = 6.3 Hz, H-1″), 6.20 (1H, s, H-6), 6.39 (1H, s, H-8), 6.88 (2H, d, J = 8.5 Hz, H-3′, 5′), 8.05 (2H, d, J = 8.5 Hz, H-2′, 6′). ^{13}C-NMR (150 MHz, CD_3OD): δ_C 66.8 (C-5″), 68.9 (C-4″), 72.8 (C-2″), 74.0 (C-3″), 94.7 (C-8), 99.9 (C-6), 104.4 (C-1″), 105.7 (C-10), 116.2 (C-3′, C-5′), 122.6 (C-1′), 132.3 (C-2′, C-6′), 135.6 (C-3), 158.4 (C-2), 158.8 (C-9), 161.6 (C-4′), 163.0 (C-5), 166.0 (C-7), 179.6 (C-4).

*Kaempferol-3-O-β-D-galactopyranoside (**32**)*, amorphous yellow powder, $C_{21}H_{20}O_{11}$. ESI-MS (negative) *m/z* 447.22 [M-H]$^-$. UV (MeOH) λ_{max} 264, 346 nm. ^{1}H-NMR (600 MHz, CD_3OD): δ_H 3.44 (1H, t, J = 6.1 Hz, H-5″), 3.52 (2H, m, H-3″, 6″b), 3.62 (1H, dd, J = 5.9, 11.2 Hz, H-6″a), 3.78 (1H, dd, J = 8.2, 9.3 Hz, H-2″), 3.82 (1H, d, J = 2.8 Hz, H-4″), 5.13 (1H, d, J = 7.7 Hz, H-1″), 6.20 (1H, s, H-6), 6.40 (1H, s, H-8), 6.87 (1H, d, J = 8.7 Hz, H-3′, 5′), 8.08 (2H, d, J = 8.7 Hz, H-2′, 6′). ^{13}C-NMR (150 MHz, CD_3OD): δ_C 62.0 (C-6″), 70.0 (C-4″), 73.0 (C-2″), 75.0 (C-3″), 77.1 (C-5″), 94.8 (C-8), 99.9 (C-6), 105.0 (C-1″), 105.7 (C-10), 116.1 (C-3′, 5′), 122.7 (C-1′), 132.4 (C-2′, 6′), 135.6 (C-3), 158.5 (C-9), 159.1 (C-2), 161.6 (C-4′), 163.1 (C-5), 166.1 (C-7), 179.7 (C-4).

*Kaempferol-3-O-β-D-glucopyranoside (**33**),* amorphous yellow powder, $C_{21}H_{20}O_{11}$. HR-ESI-MS (negative) *m/z* 447.0931 [M-H]$^-$ (calcd. for 447.0927). UV (MeOH) λ_{max} 264, 348 nm. ^{1}H-NMR (600 MHz, CD_3OD): δ_H 3.20 (1H, ddd, J = 2.1, 5.5, 9.1 Hz, H-5″), 3.34 (1H, brs, H-4″), 3.44 (1H, m, H-2″), 3.41(1H, t, J = 9.2 Hz, H-3″), 3.52 (1H, dd, J = 5.5, 11.8 Hz, H-6″b), 3.69 (1H, dd, J = 2.1, 11.8

Hz, H-6″a), 5.25 (1H, d, J = 7.3 Hz, H-1″), 6.20 (1H, s, H-6), 6.40 (1H, s, H-8), 6.88 (2H, d, J = 8.6 Hz, H-3′, 5′), 8.05 (2H, dd, J = 8.6 Hz, H-2′, 6′). ^{13}C-NMR (150 MHz, CD_3OD): δ_C 62.6 (C-6″), 71.3 (C-4″), 75.7 (C-2″), 78.0 (C-3″), 78.4 (C-5″), 94.7 (C-8), 99.9 (C-6), 104.0 (C-1″), 105.7 (C-10), 116.1 (C-3′, C-5′), 122. 8 (C-1′), 132.3 (C-2′, C-6′), 135.4 (C-3), 158.5 (C-2), 159.1 (C-9), 161.6 (C-4′), 163.1 (C-5), 166.0 (C-7), 179.5 (C-4).

*Kaempferol-3-O-robinobioside (**34**),* amorphous yellow powder, $C_{27}H_{30}O_{15}$. ESI-MS (negative) *m/z* 593.1534 $[M-H]^-$ (calcd. for 593.1507). UV (MeOH) λ_{max} 264, 346 nm. ^{1}H-NMR (600 MHz, CD_3OD): δ_H 1.18 (3H, d, J = 6.2 Hz, H-6‴), 3.38 (1H, dd, J = 6.8, 10.1 Hz, H-6″a), 3.49-3.79 (8H, H-2″, 3″, 4″, 5″, 2‴, 3‴, 4‴, 5‴), 3.71 (1H, dd, J = 5.7, 10.2 Hz, H-6″b), 4.52 (1H, s, H-1‴), 5.04 (1H, d, J = 7.8 Hz, H-1″), 6.21 (1H, s, H-6), 6.40 (1H, s, H-8), 6.88 (2H, d, J = 8.5 Hz, H-3', 5'), 8.09 (2H, d, J = 8.5 Hz, H-2', H-6'). ^{13}C-NMR (150 MHz, CD_3OD): δ_C 18.0 (C-6‴), 67.4 (C-6″), 69.7 (C-5‴) , 70.1 (C-4″), 72.0 (C-2″), 72.3 (C-3‴), 73.0 (C-2‴), 73.8 (C-4‴), 75.0 (C-3″), 75.3 (C-5″), 94.9 (C-8), 100.0 (C-6), 101.9 (C-1), 105.5 (C-1″), 105.6 (C-10), 116.1 (C-3', 5'), 122.6 (C-1'), 132.5 (C-2', 6'), 135.7 (C-3), 158.5 (C-2), 159.3 (C-9), 161.6 (C-4'), 163.0 (C-5), 166.1 (C-7), 179.6 (C-4).

*Kaempferol-3-O-α-L-rhamnopyranosyl-(1→6)-β-D-glucopyranoside (**35**)*, amorphous yellow powder, $C_{27}H_{30}O_{15}$. ESI-MS (negative) *m/z* 593.38 $[M-H]^-$. UV (MeOH) λ_{max} 264, 346 nm. ^{1}H-NMR (600 MHz, CD_3OD): δ_H 1.12 (3H, d, J = 6.2 Hz, H-6‴ CH_3), 3.25 (1H, m, H-4″), 3.27 (1H, m, H-4‴), 3.32-3.46 (5H, m, H-2″, 3″, 5″, 6″b, H-5‴), 3.55 (1H, dd, J = 3.4, 9.5 Hz, H-3‴), 3.62 (1H, dd, J = 1.6, 3.4 Hz, H-2‴), 3.80 (1H, dd, J = 1.5, 11 Hz, H-6″a), 4.51 (1H, d, J = 1.4 Hz, H-1‴), 5.13 (1H, d, J = 7.5 Hz, H″-1), 6.21 (1H, d, J = 2.0 Hz, H-6), 6.41 (1H, d, J = 2.0 Hz, H-8), 6.89 (2H, d, J = 9.0 Hz, H-3′, 5′), 8.06 (2H, d, J = 9.0 Hz, H-2′, 6′). ^{13}C-NMR (150 MHz, CD_3OD): δ_C 18.0 (C-6‴), 68.6 (C-6″), 69.8 (C-5‴), 71.5 (C-4″), 72.1 (C-2‴), 72.3 (C-3‴), 73.9 (C-4‴), 75.8 (C-2″), 77.3 (C-5″), 78.2 (C-3″), 95.0 (C-8), 100.1 (C-6), 102.5 (C-1‴), 104.6 (C-1″), 105.7 (C-10), 116.2 (C-3′, 5′), 122.8 (C-1′), 132.4 (C-2′, 6′), 135.5 (C-3), 158.6 (C-2), 159.5 (C-9), 161.6 (C-5′), 163.1 (C-5), 166.3 (C-7), 179.5 (C-4).

*Kaempferol-3-O-[α-L-rhamnopyranosyl-(1→6)]-[α-L-rhamnopyranosyl-(1→2)]-β-D-galactopyranoside (**36**)*, amorphous yellow powder, $C_{33}H_{40}O_{19}$. ESI-MS (negative): *m/z* 739.54 $[M-H]^-$. UV (MeOH) λ_{max} 264, 346 nm. ^{1}H-NMR (600 MHz, CD_3OD): δ_H 0.97 (3H, d, J = 6.2 Hz, H-6'''), 1.17 (3H, d, J = 6.2 Hz, H-6''''), 3.26 (1H, m, H-4''''), 3.49 (1H, m, H-3''''), 3.50 (1H, m, H-5''''), 3.34 (1H, m, H-4'''), 3.44 (1H, m, H-6''b), 3.55 (1H, m, H-2''''), 3.62 (1H, m, H-5''), 3.69 (1H, m, H-6''a), 3.72 (1H, m, H-3''), 3.76 (1H, m, H-3'''), 3.78 (1H, m, H-4''), 3.92 (1H, dd, J = 7.9, 9.5 Hz, H-2''),

3.99 (1H, m, H-2'''), 4.06 (1H, m, H-5'''), 4.52 (1H, s, H-1''''), 5.21 (1H, s, H-1'''), 5.60 (1H, d, *J* = 7.7 Hz, H-1''), 6.19 (1H, s, H-6), 6.38 (1H, s, H-8), 6.89 (2H, d, *J* = 8.8 Hz, H-3', 5'), 8.06 (2H, d, *J* = 8.8 Hz, H-2', 6'). ^{13}C-NMR (150 MHz, CD_3OD): δ_C 17.5 (C-6'''), 17.9 (C-6''''), 67.2 (C-6''), 69.7 (C-5''''), 69.8 (C-5'''), 70.7 (C-4''), 72.1 (C-2''''), 72.3 (C-3''''), 72.3 (C-3'''), 72.4 (C-2'''), 73.9 (C-4''''), 74.1 (C-4'''), 75.3 (C-5''), 75.7 (C-3''), 77.6 (C-2''), 94.7 (C-8), 99.8 (C-6), 100.9 (C-1''), 101.9 (C-1''''), 102.6 (C-1'''), 105.9 (C-10), 116.2 (C-3', 5'), 123.0 (C-1'), 132.2 (C-2', 6'), 134.5 (C-3), 158.4 (C-9), 158.7 (C-2), 161.3 (C-4'), 163.2 (C-5), 165.7 (C-7), 179.4 (C-4).

*Kaempferol-3-O-[α-L-rhamnopyranosyl-(1→6)]-[α-L-rhamnopyranosyl-(1→2)]-β-D-glucopyranoside (**37**),* amorphous yellow powder, $C_{33}H_{40}O_{19}$. ESI-MS (negative): *m/z* 739.38 [M-H]$^-$. UV (MeOH) λ_{max} 264, 346 nm. ^{1}H-NMR (600 MHz, CD_3OD): δ_H 0.98 (3H, d, *J* = 6.3 Hz, H-6''''), 1.08 (3H, d, *J* = 6.3 Hz, H-6'''), 3.22-4.07 (15H, sugar-H), 4.50 (1H, brs, H-1'''), 5.20 (1H, brs, H-1''''), 5.60 (1H, d, *J* = 7.6 Hz, H-1''), 6.19 (1H, brs, H-6), 6.38 (1H, brs, H-8), 6.89 (2H, d, *J* = 8.8 Hz, H-3', 5'), 8.02 (2H, d, *J* = 8.8 Hz, H-2', 6'). ^{13}C-NMR (150 MHz, CD_3OD): δ_C 17.5 (C-6''''), 17.8 (C-6'''), 68.3 (C-6''), 69.7 (C-5'''), 69.9 (C-5''''), 71.9 (C-4''), 72.1 (C-2'''), 72.3 (C-3'''), 72.3 (C-3''''), 72.4 (C-2''''), 73.8 (C-4'''), 74.0 (C-4''''), 77.1 (C-5''), 78.9 (C-3''), 79.9 (C-2''), 94.8 (C-8), 99.8 (C-6), 100.4 (C-1''), 102.3 (C-1'''), 102.6 (C-1''''), 105.9 (C-10), 116.1 (C-3', 5'), 123.2 (C-1'), 132.1 (C-2', 6'), 134.3 (C-3), 158.5 (C-9), 159.0 (C-2), 161.2 (C-4'), 163.1 (C-5), 165.7 (C-7), 179.3 (C-4).

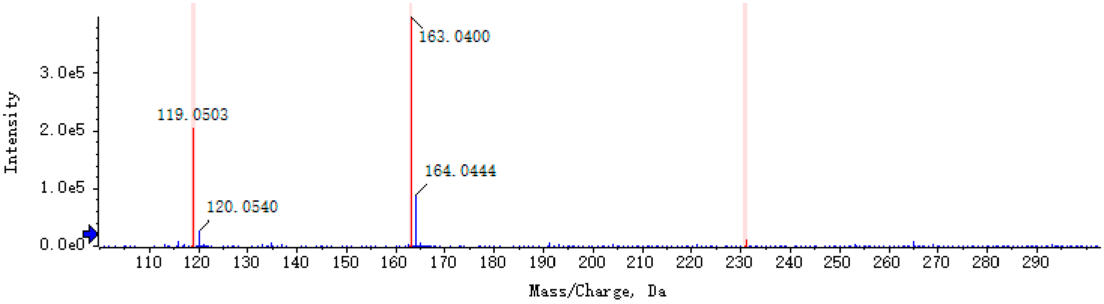

Figure S1. The HR-ESI-MS spectrum of compound 1

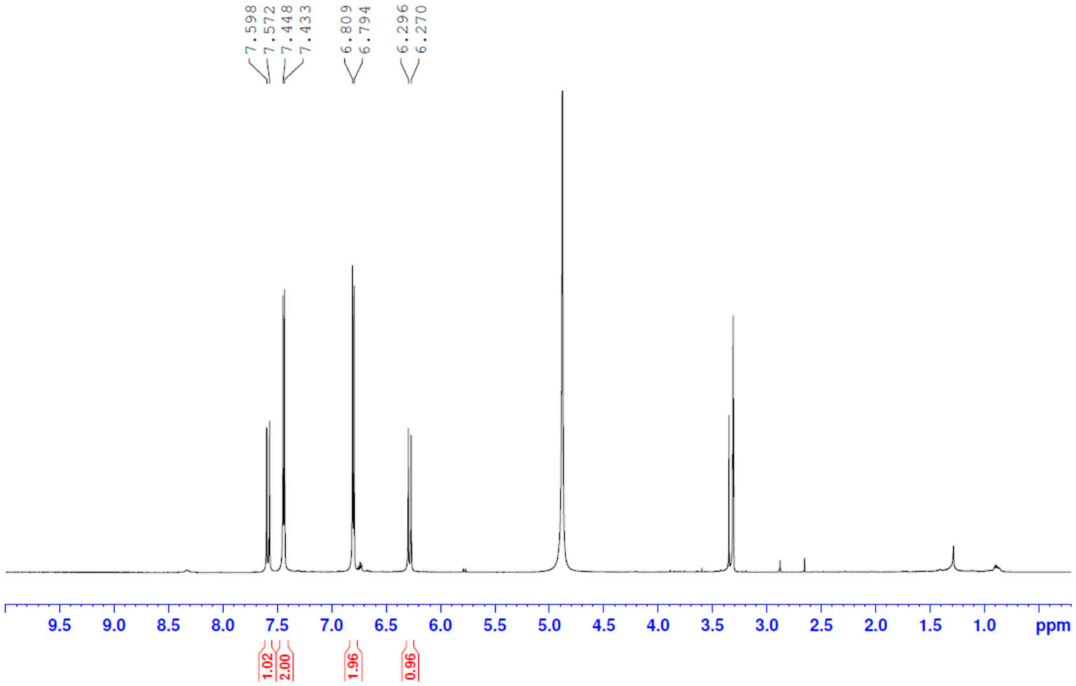

Figure S2. The ^{1}H-NMR (600MHz, CD_3OD) spectrum of compound 1

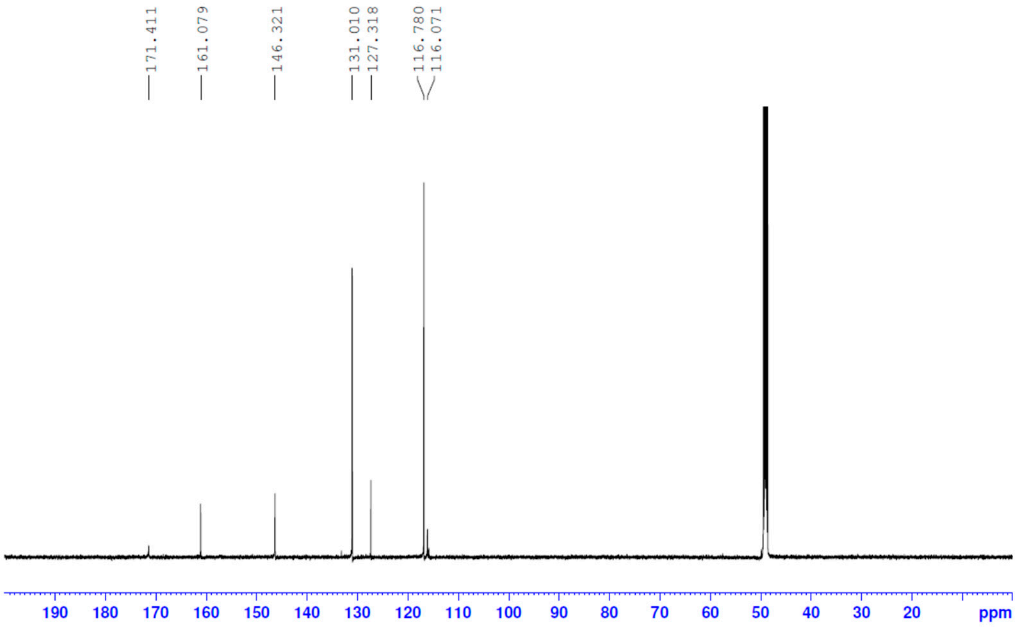

Figure S3. The ^{13}C-NMR (600MHz, CD_3OD) spectrum of compound 1

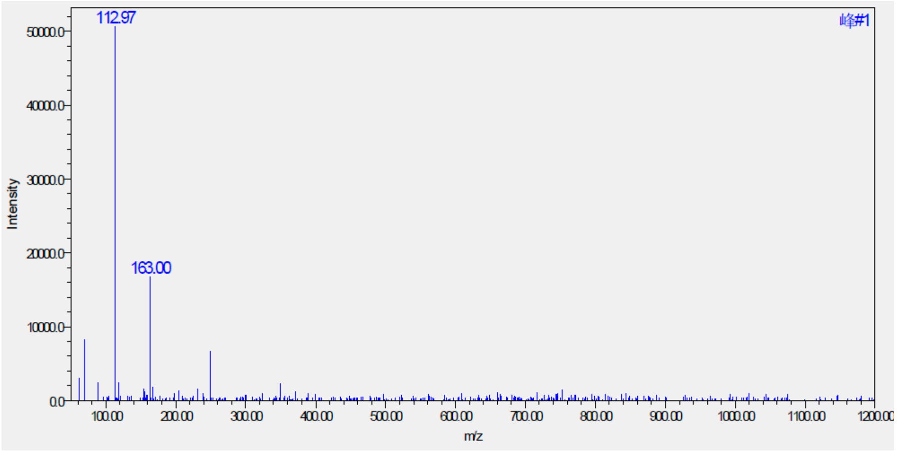

Figure S4. The ESI-MS spectrum of compound 2

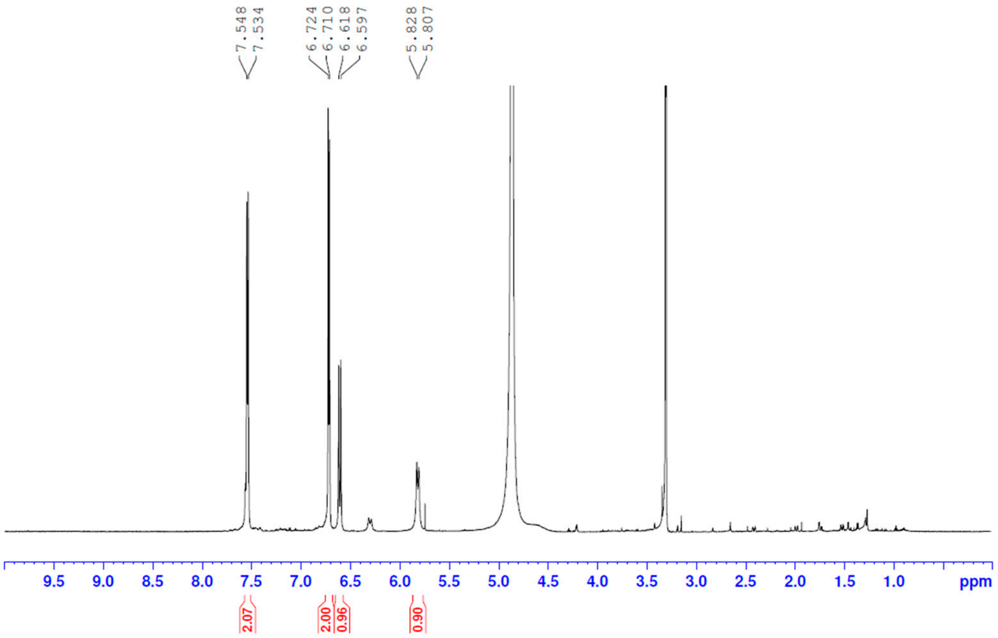

Figure S5. The ^{1}H-NMR (600MHz, CD_3OD) spectrum of compound 2

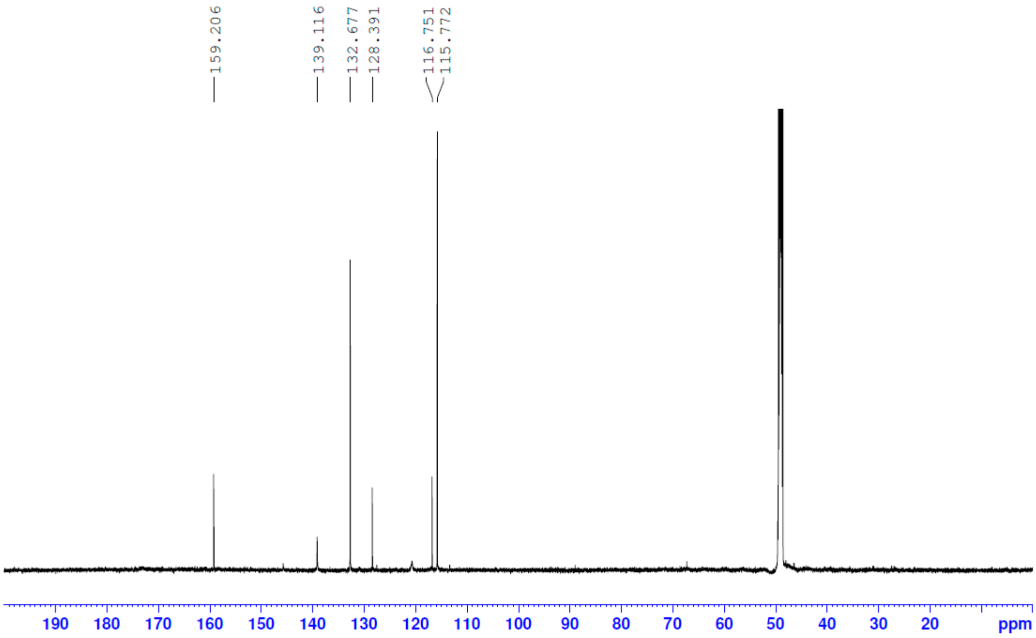

Figure S6. The ^{13}C-NMR (600MHz, CD_3OD) spectrum of compound 2

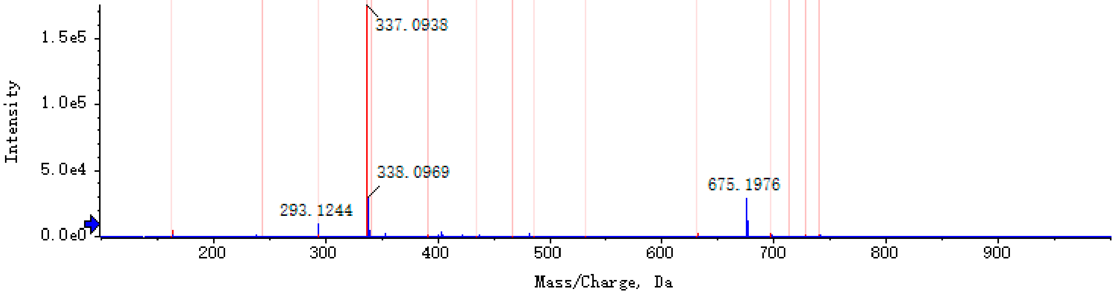

Figure S7. The HR-ESI-MS spectrum of compound 3

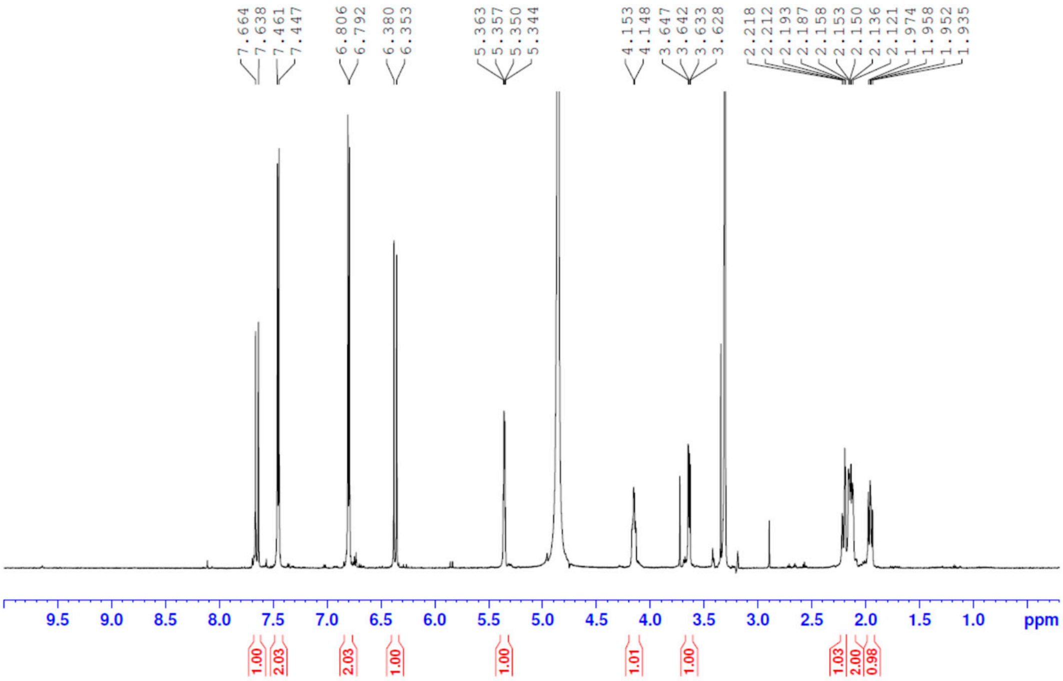

Figure S8. The ^{1}H-NMR (600MHz, CD_3OD) spectrum of compound 2

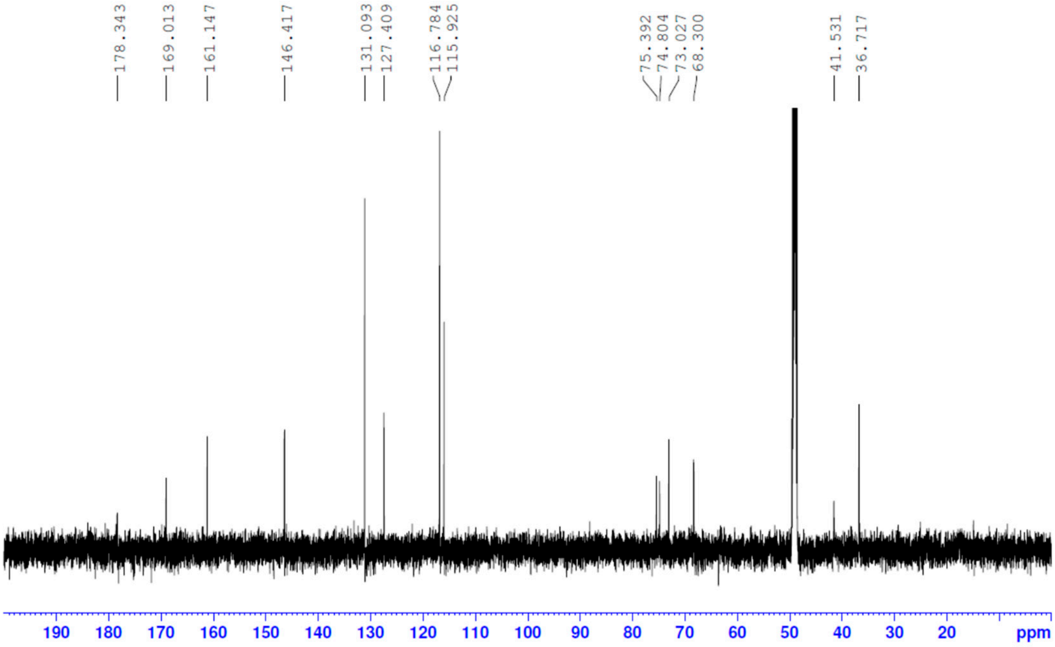

Figure S9. The ^{13}C-NMR (150MHz, CD_3OD) spectrum of compound 3

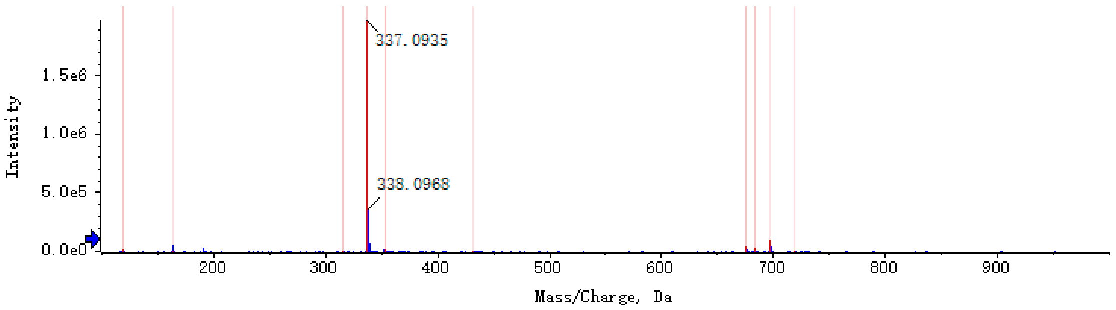

Figure S10. The HR-ESI-MS spectrum of compound 4

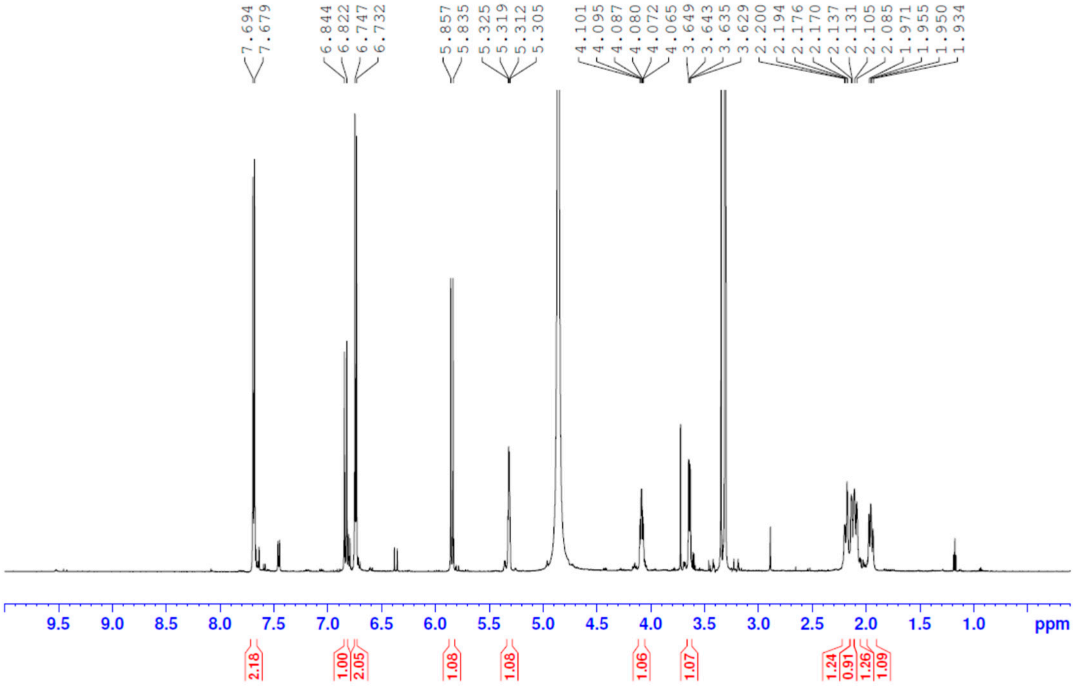

Figure S11. The ^{1}H-NMR (600MHz, CD_3OD) spectrum of compound 4

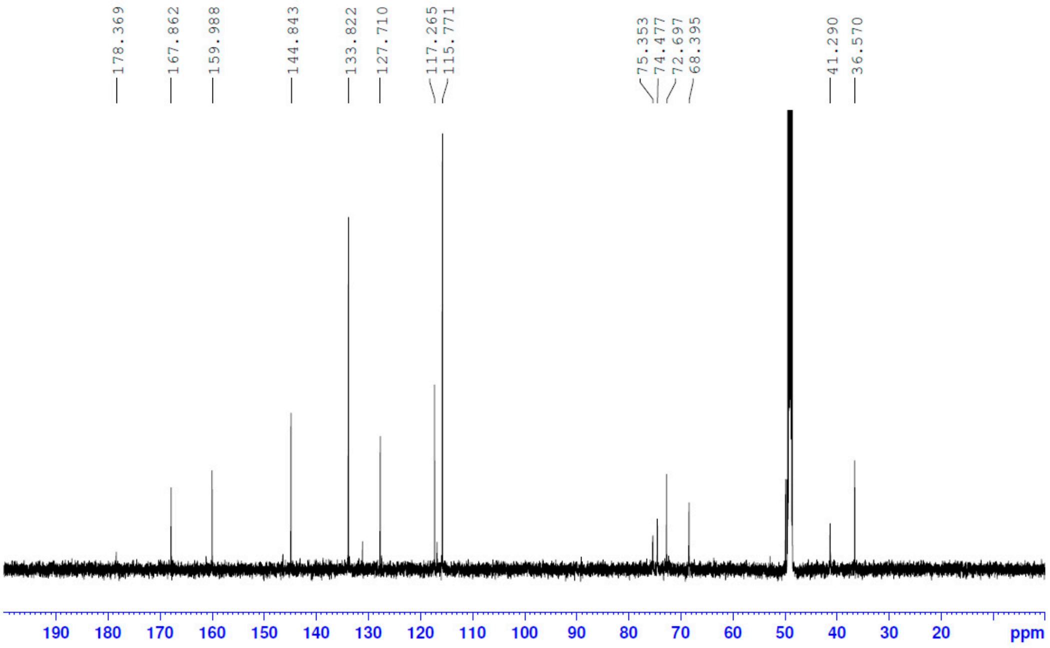

Figure S12. The ^{13}C-NMR (150MHz, CD_3OD) spectrum of compound 4

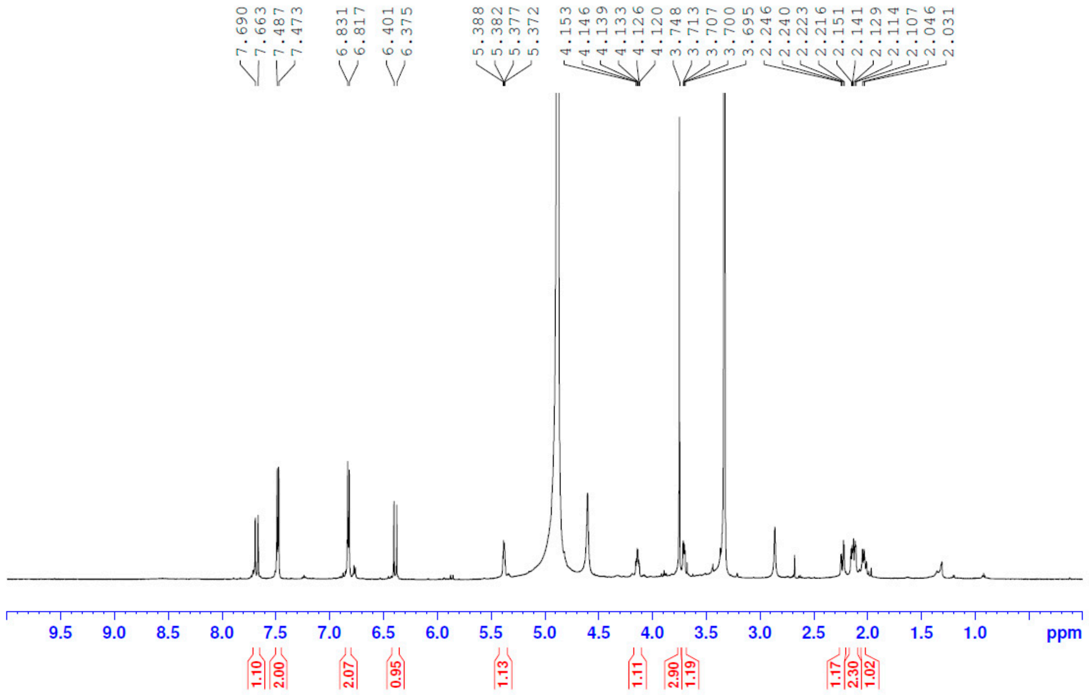

Figure S13. The ^{1}H-NMR (600MHz, CD_3OD) spectrum of compound 5

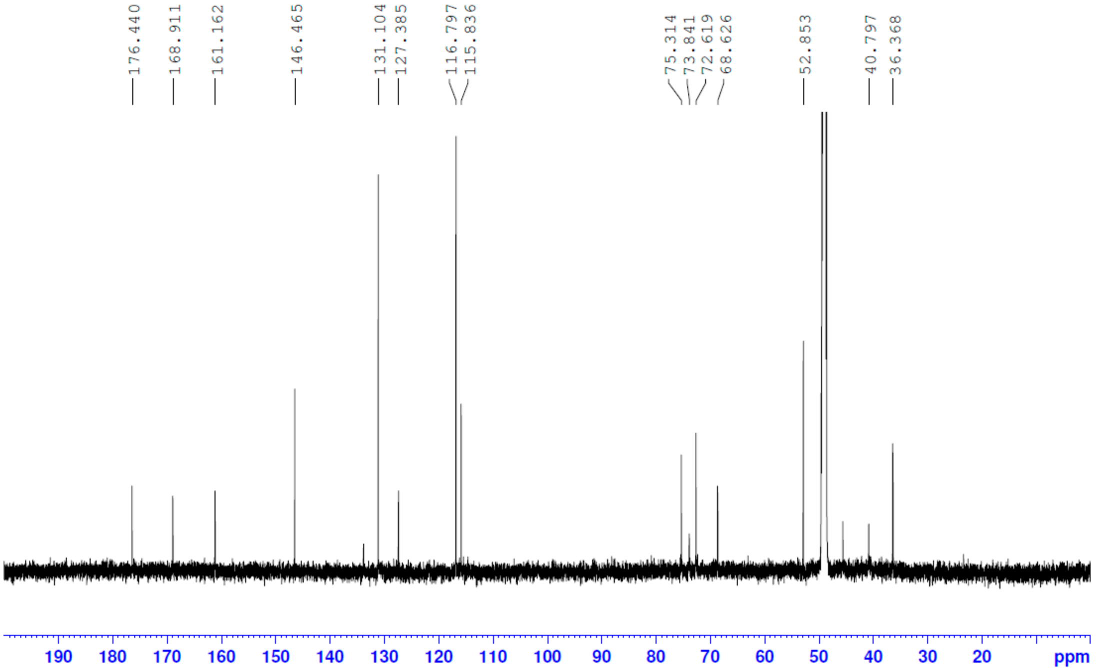

Figure S14. The ^{1}H-NMR (150MHz, CD_3OD) spectrum of compound 5

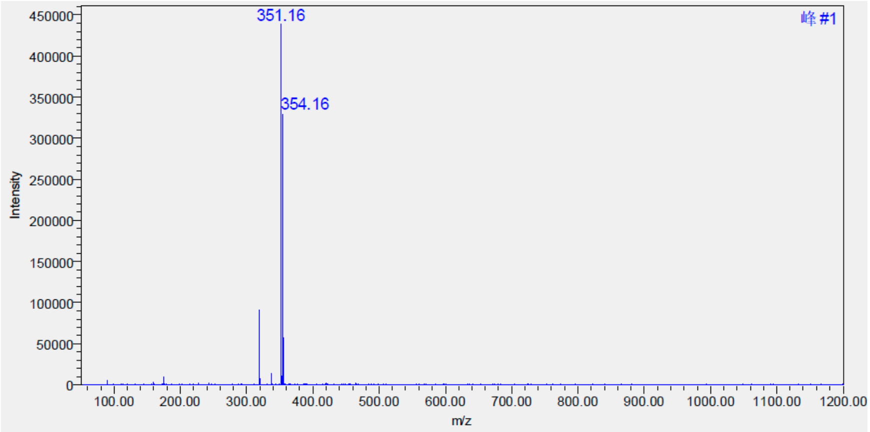

Figure S15. The ESI-MS spectrum of compound 6

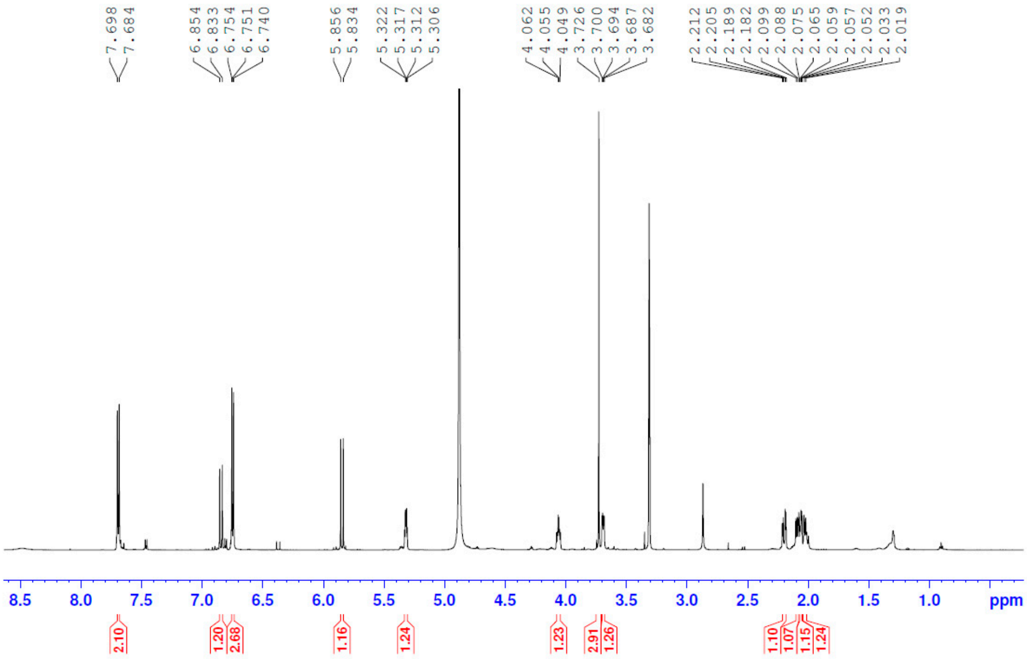

Figure S16. The ^{1}H-NMR (600MHz, CD_3OD) spectrum of compound 6

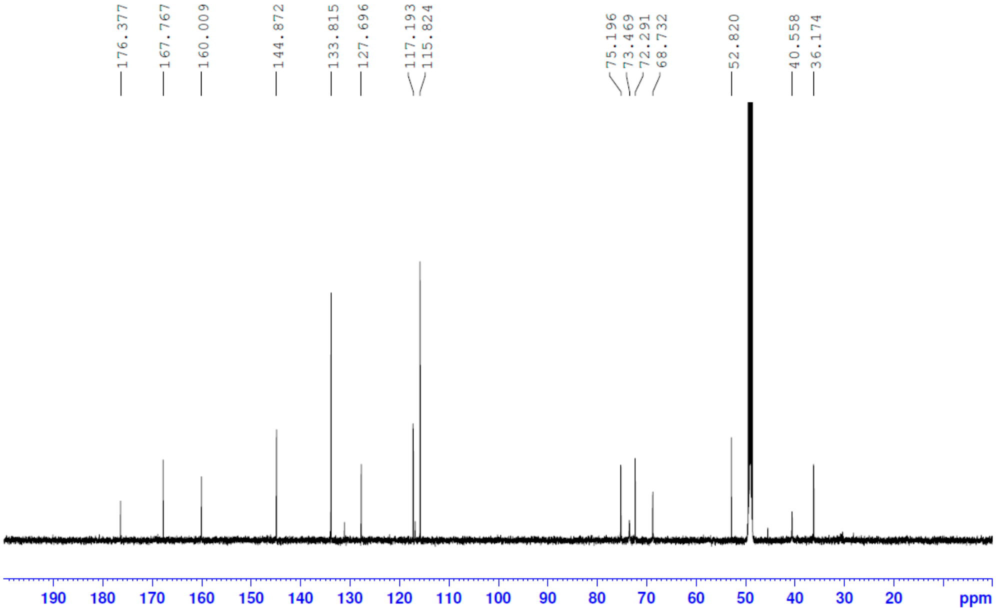

Figure S17. The ^{13}C-NMR (150MHz, CD_3OD) spectrum of compound 6

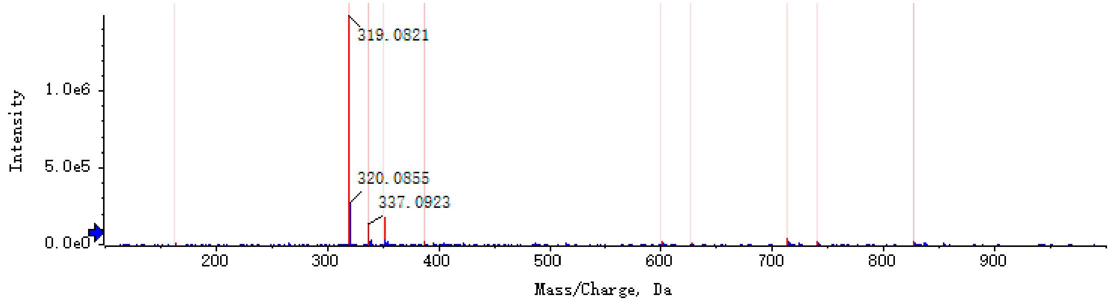

Figure S18. The HR-ESI-MS spectrum of compound 7

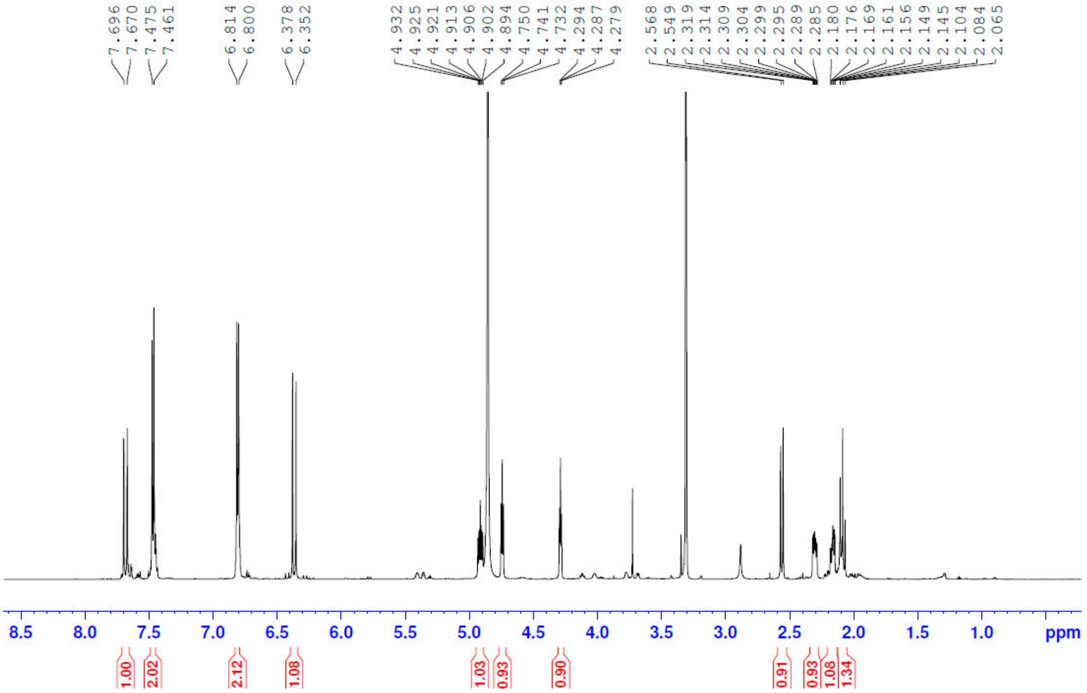

Figure S19. The ^{1}H-NMR (600MHz, CD_3OD) spectrum of compound 7

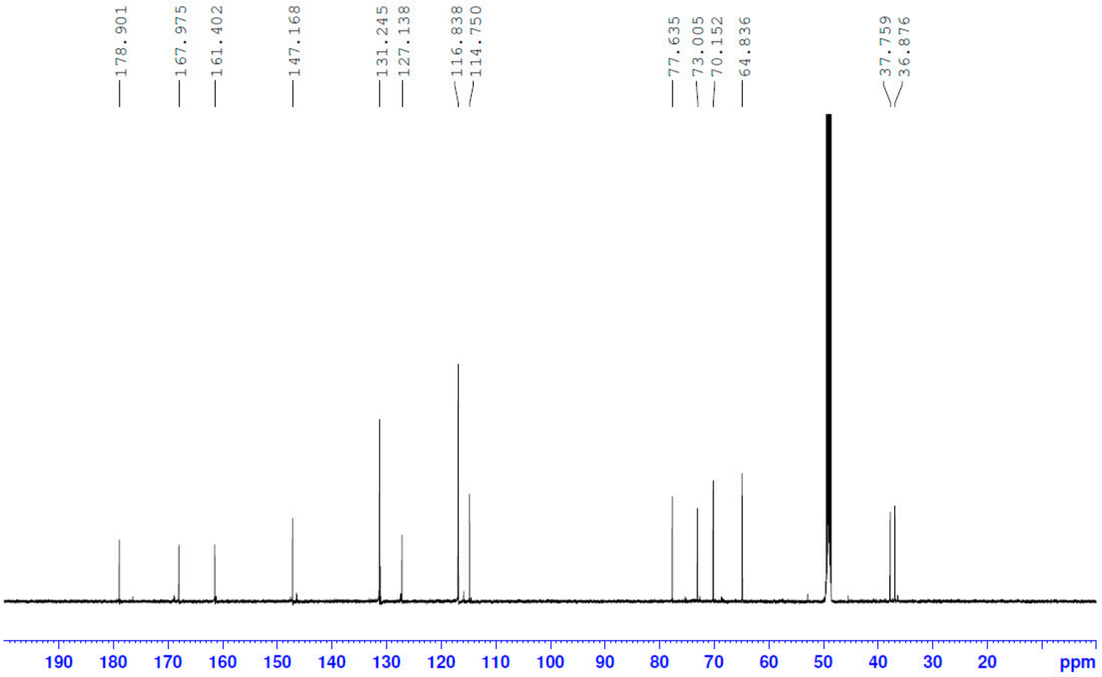

Figure S20. The ^{13}C-NMR (150MHz, CD_3OD) spectrum of compound 7

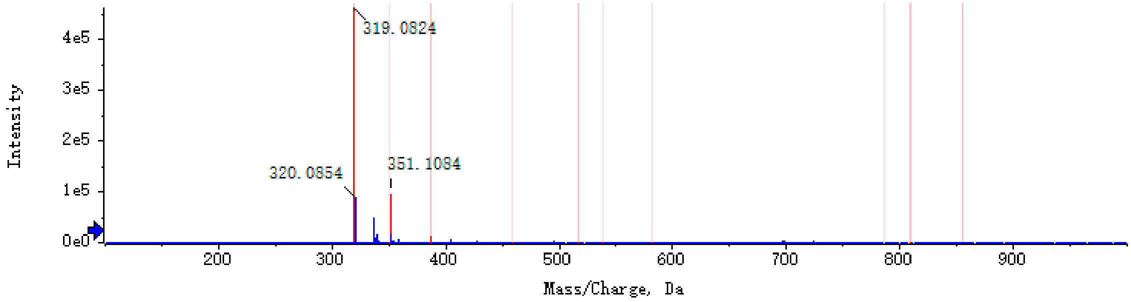

Figure S21. The HR-ESI-MS spectrum of compound 8

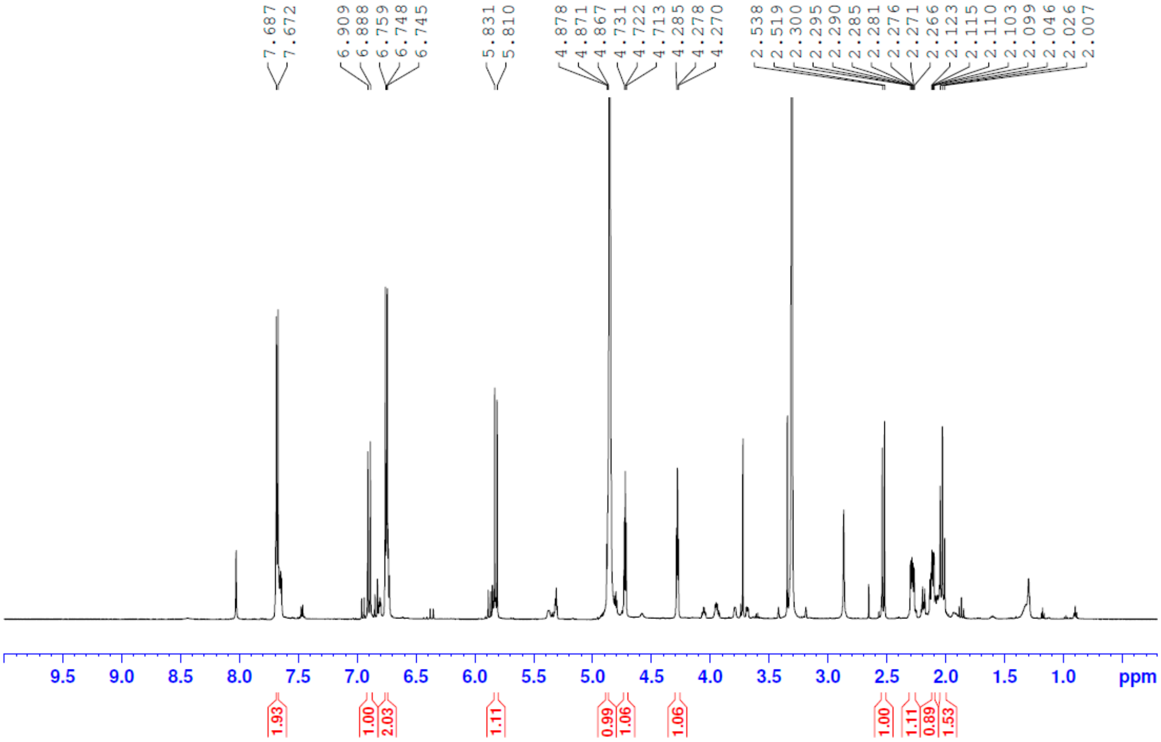

Figure S22. The ^{1}H-NMR (600MHz, CD_3OD) spectrum of compound 8

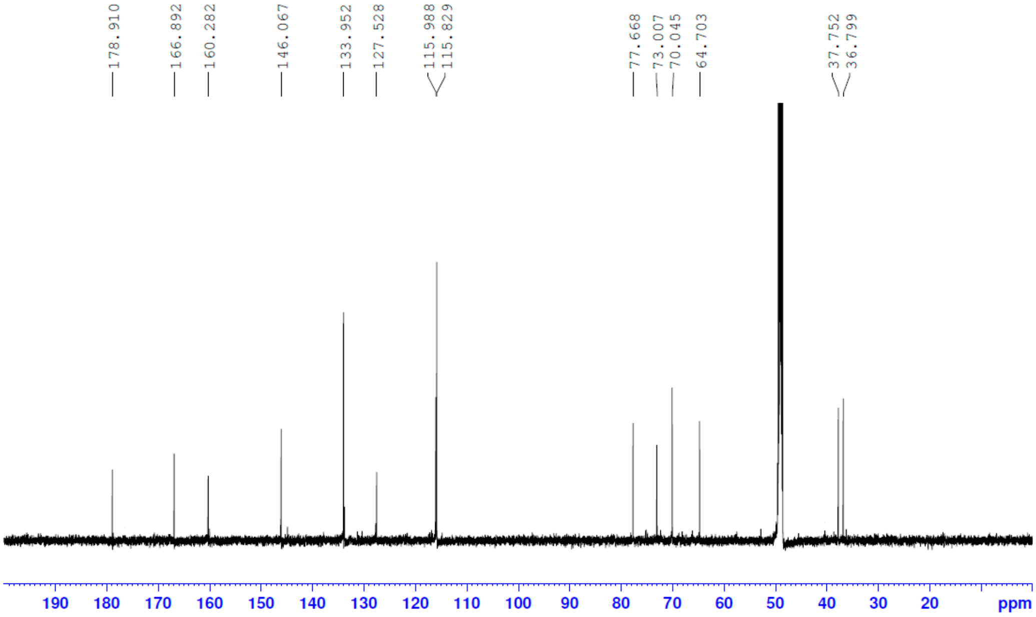

Figure S23. The ^{13}C-NMR (150MHz, CD_3OD) spectrum of compound 8

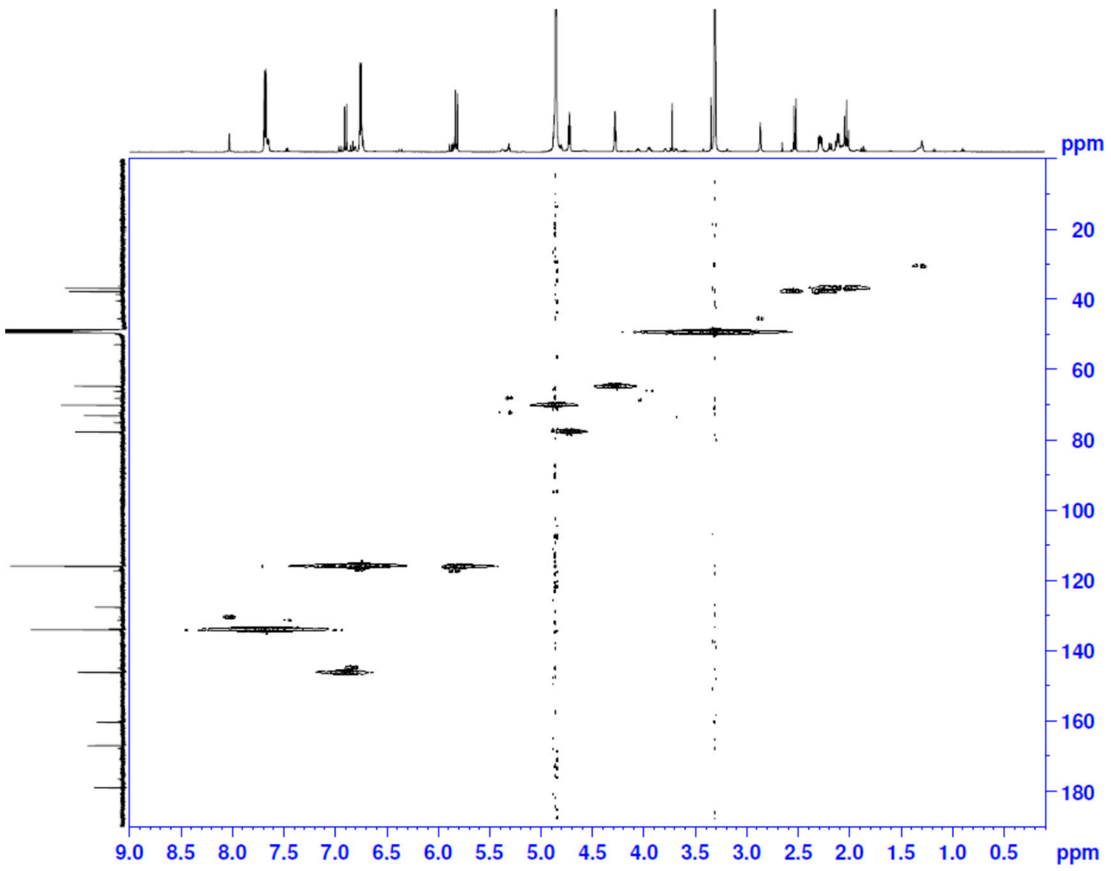

Figure S24. The HSQC spectrum of compound 8

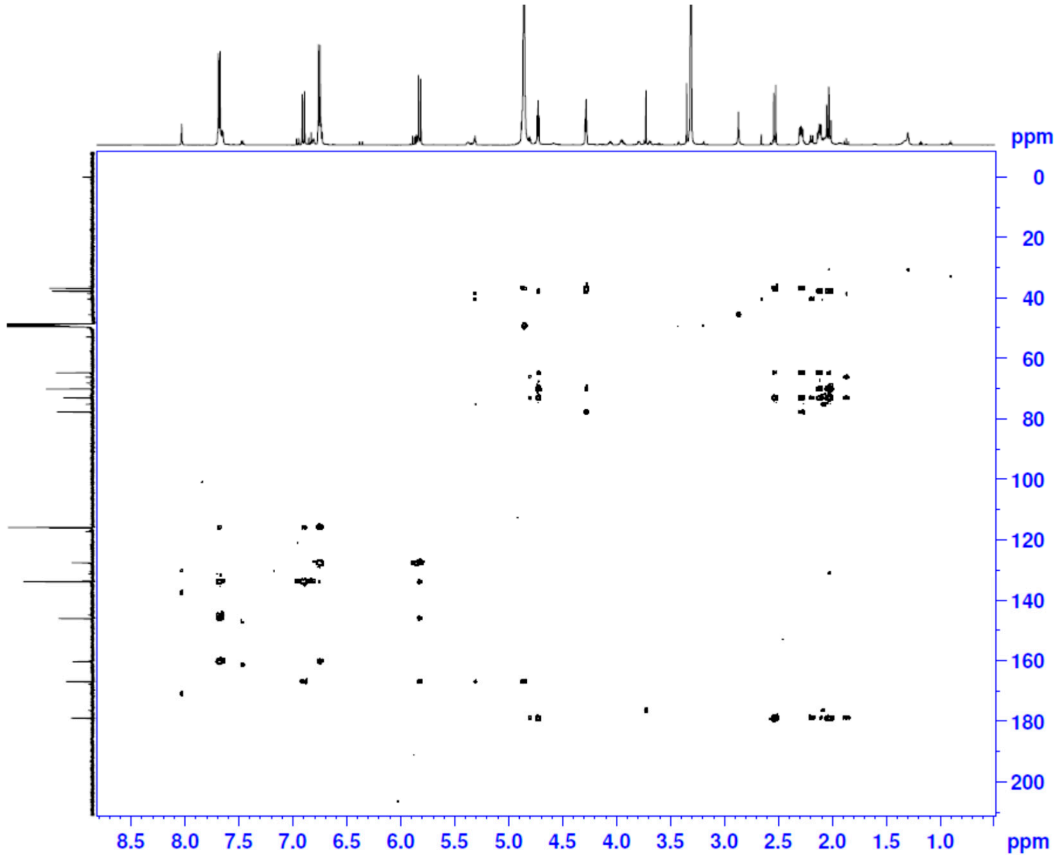

Figure S25. The HMBC spectrum of compound 8

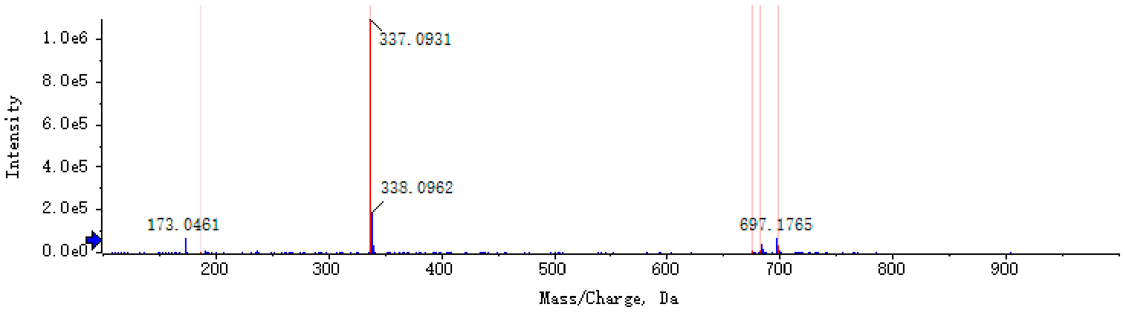

Figure S26. The HR-ESI-MS spectrum of compound 9

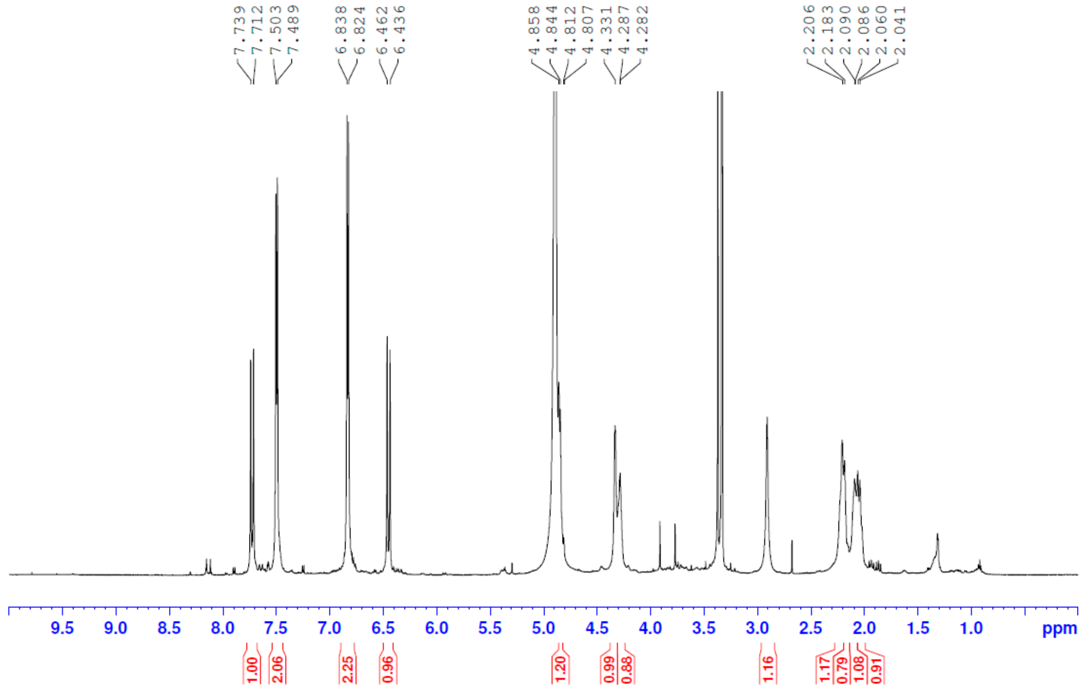

Figure S27. The ^{1}H-NMR (600MHz, CD_3OD) spectrum of compound 9

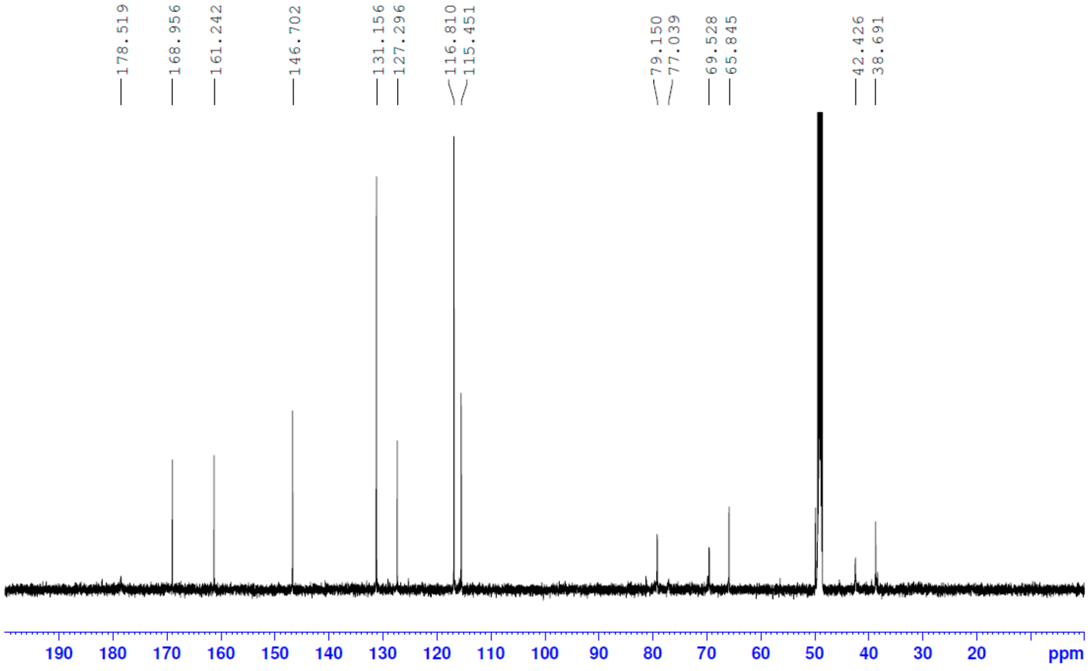

Figure S28. The ^{13}C-NMR (150MHz, CD_3OD) spectrum of compound 9

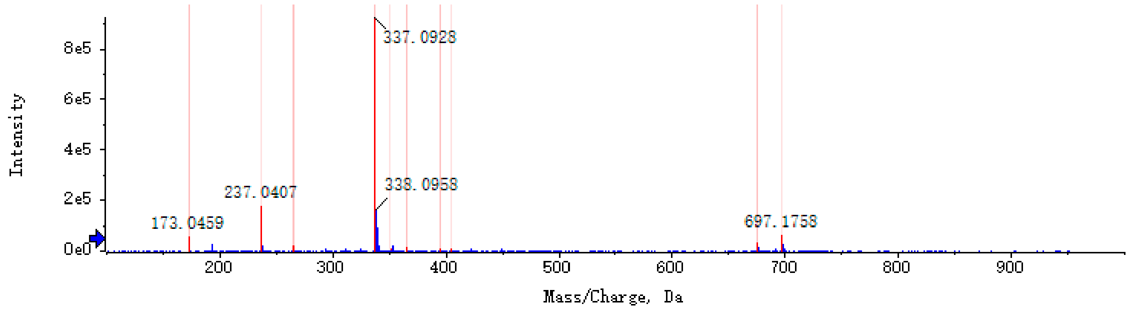

Figure S29. The HR-ESI-MS spectrum of compound 10

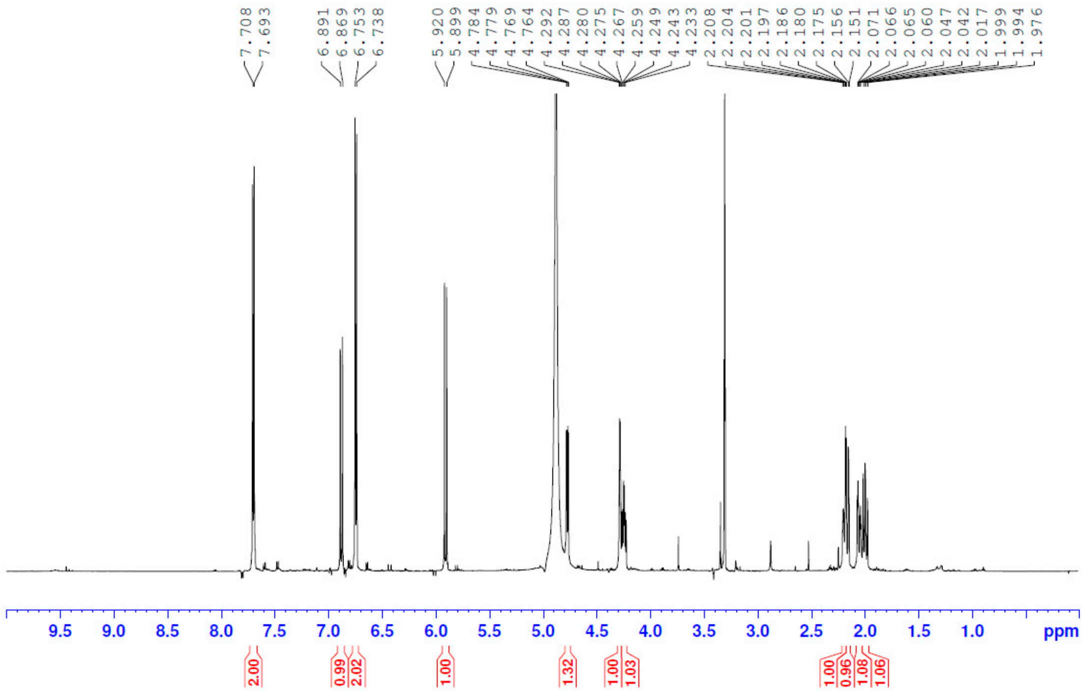

Figure S30. The ^{1}H-NMR (600MHz, CD_3OD) spectrum of compound 10

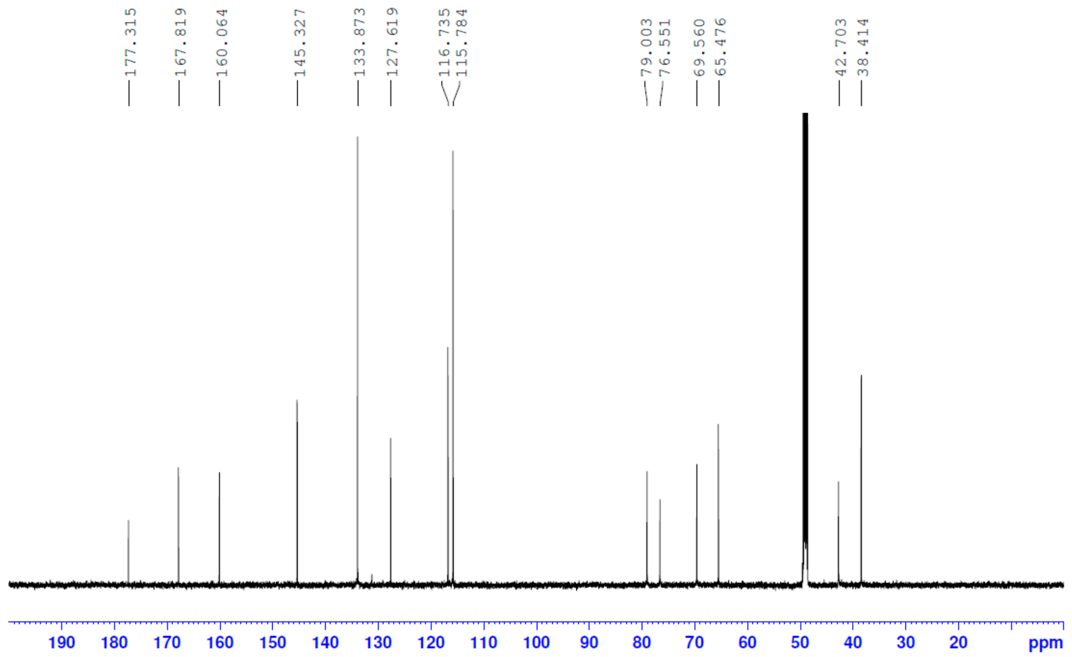

Figure S31. The ^{13}C-NMR (150MHz, CD_3OD) spectrum of compound 10

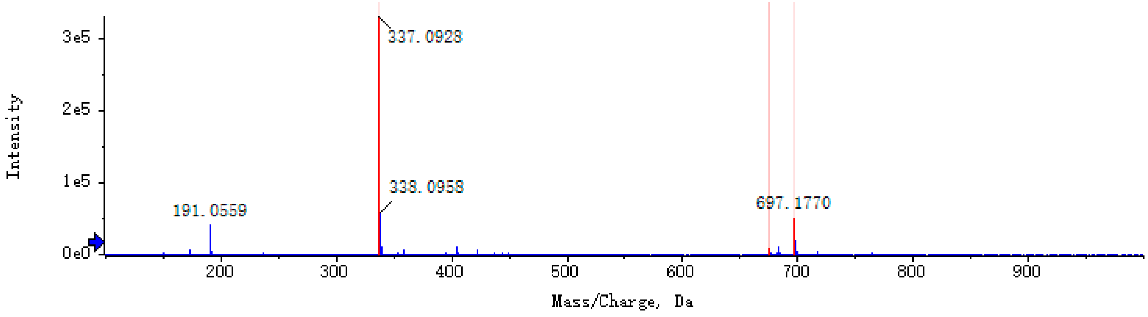

Figure S32. The HR-ESI-MS spectrum of compound 11

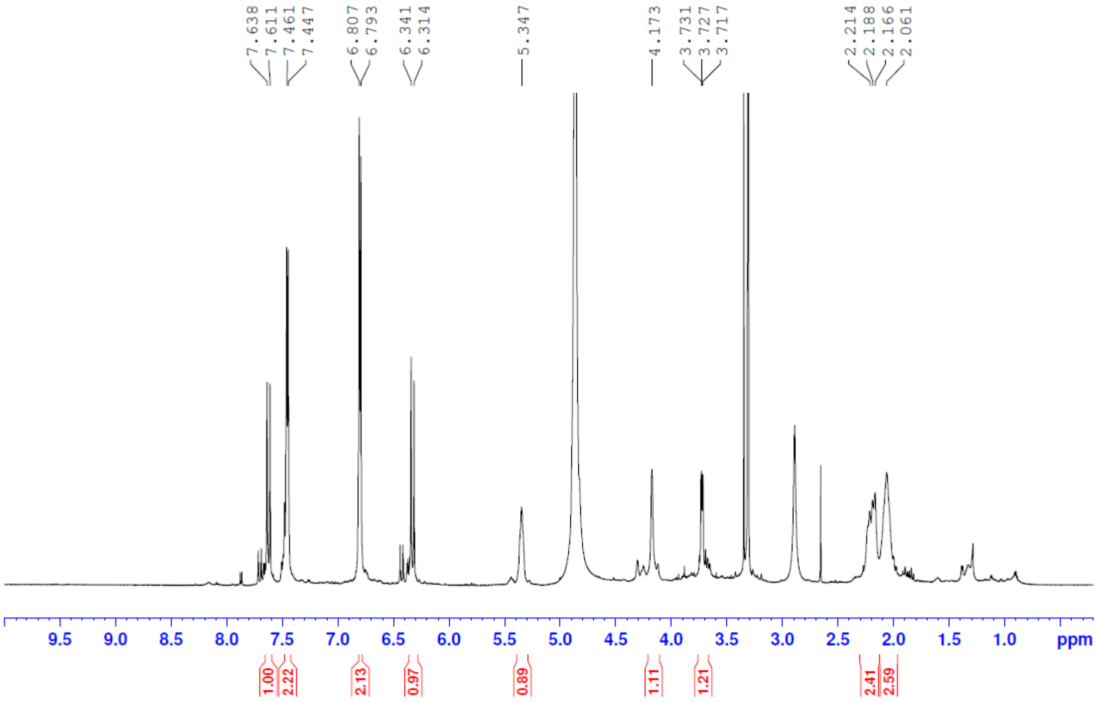

Figure S33. The ^{1}H-NMR (600MHz, CD_3OD) spectrum of compound 11

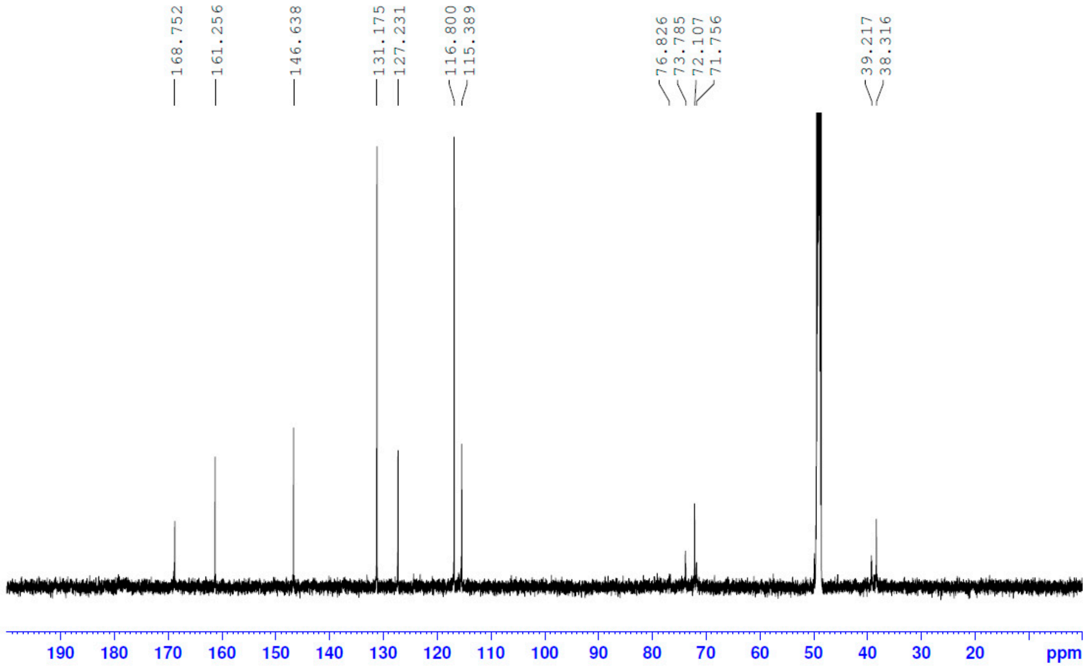

Figure S34. The ^{13}C-NMR (150MHz, CD_3OD) spectrum of compound 11

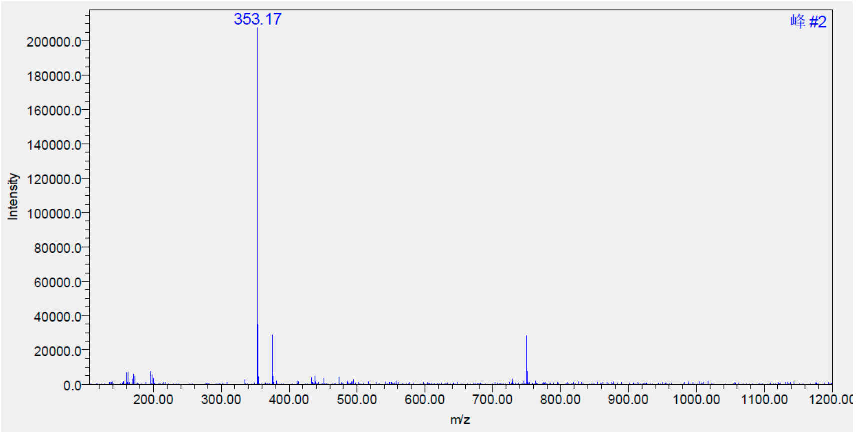

Figure S35. The ESI-MS spectrum of compound 12

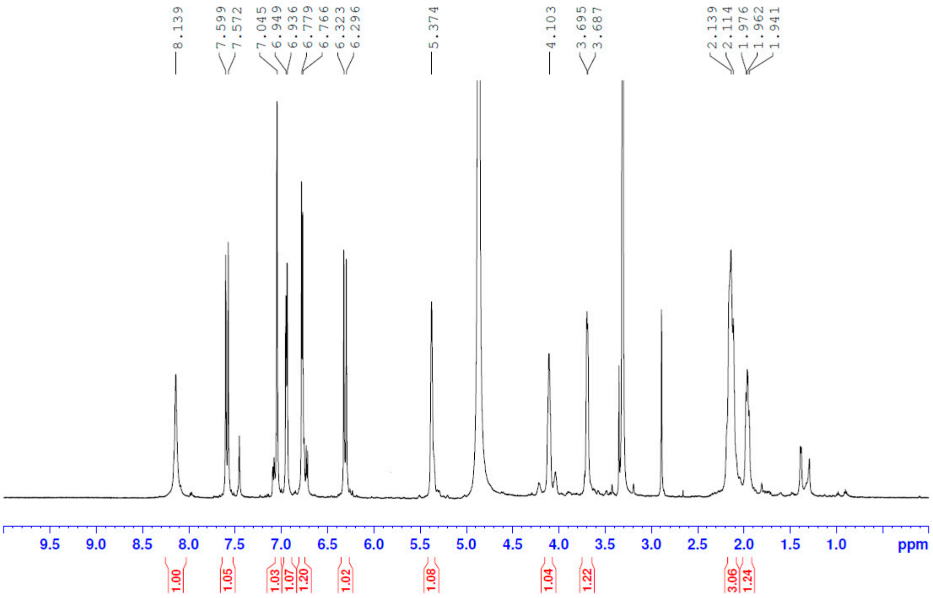

Figure S36. The ^{1}H-NMR (600MHz, CD_3OD) spectrum of compound 12

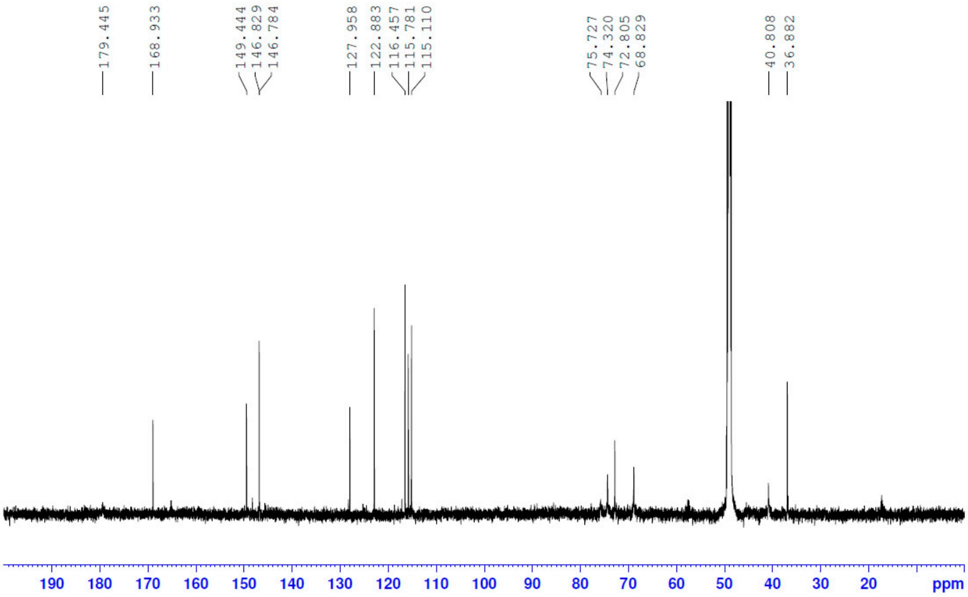

Figure S37. The ^{13}C-NMR (150MHz, CD_3OD) spectrum of compound 12

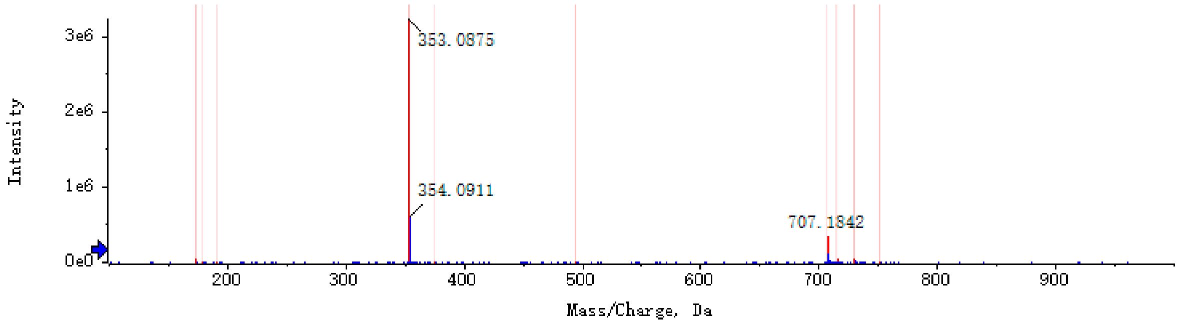

Figure S38. The ESI-MS spectrum of compound 13

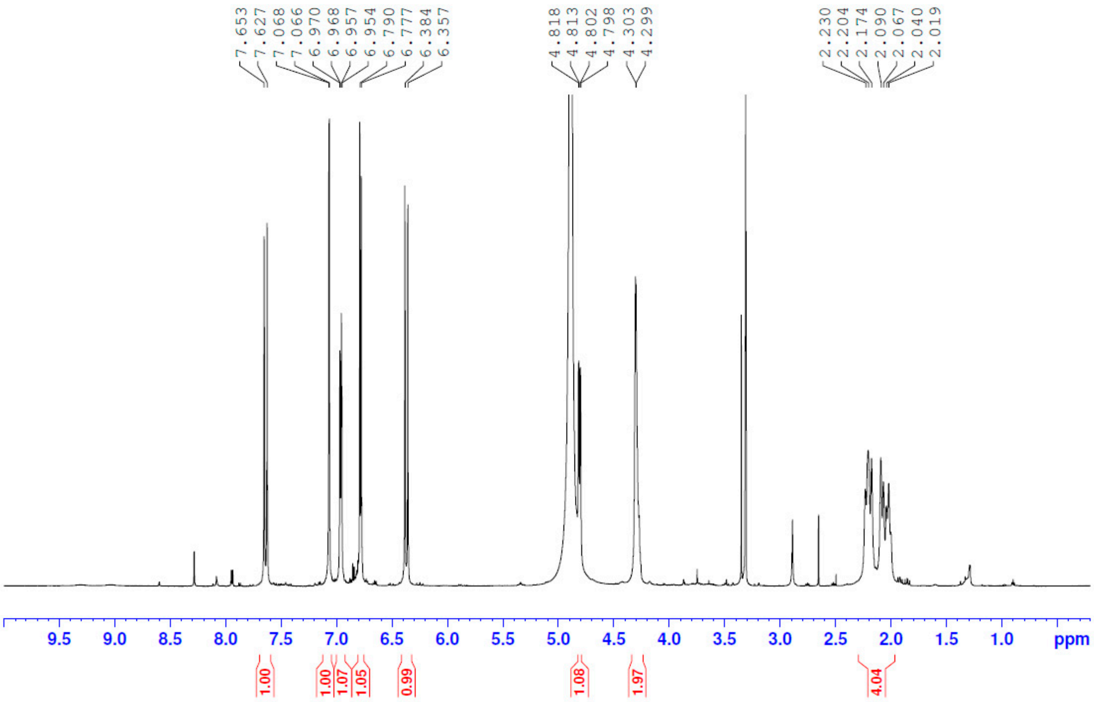

Figure S39. The ^{1}H-NMR (600MHz, CD_3OD) spectrum of compound 13

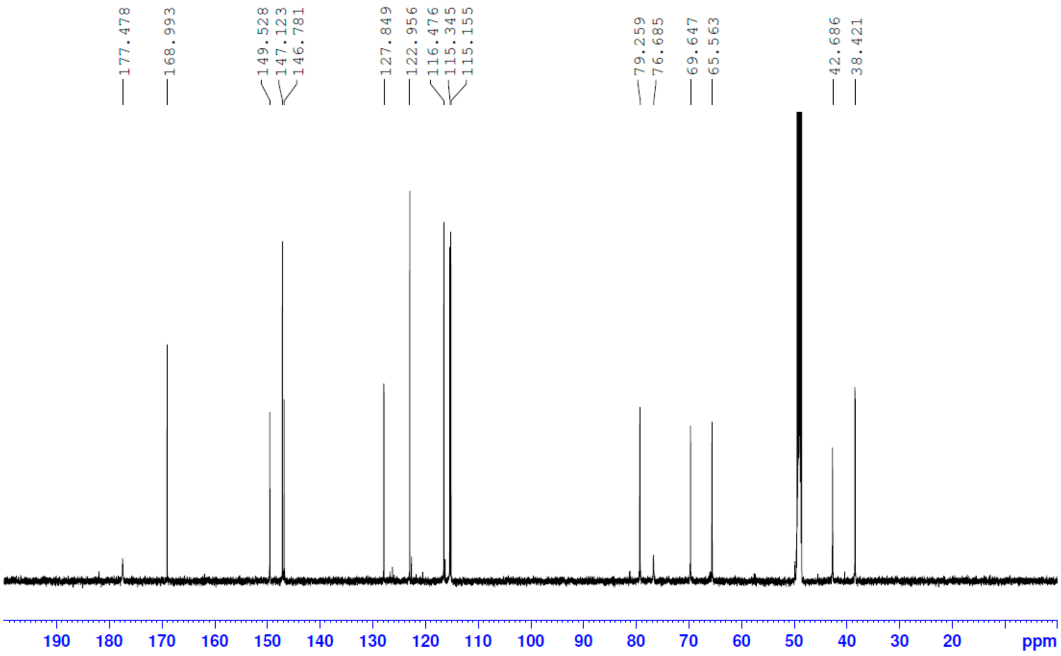

Figure S40. The ^{13}C-NMR (150MHz, CD_3OD) spectrum of compound 13

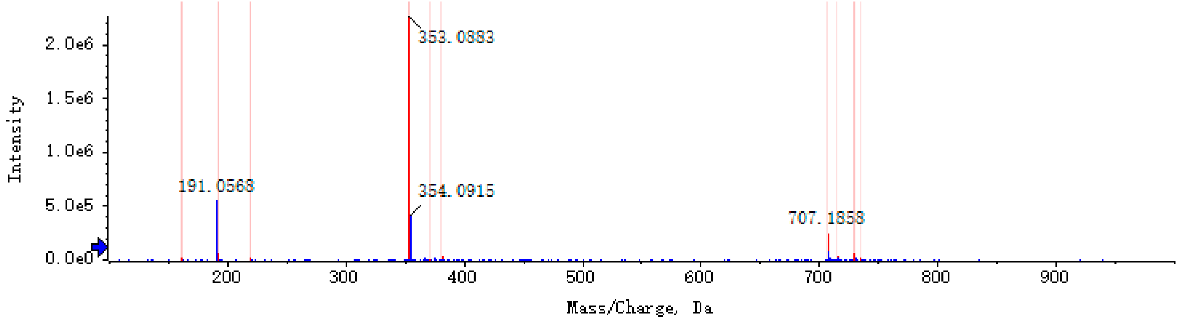

Figure S41. The HR-ESI-MS spectrum of compound 14

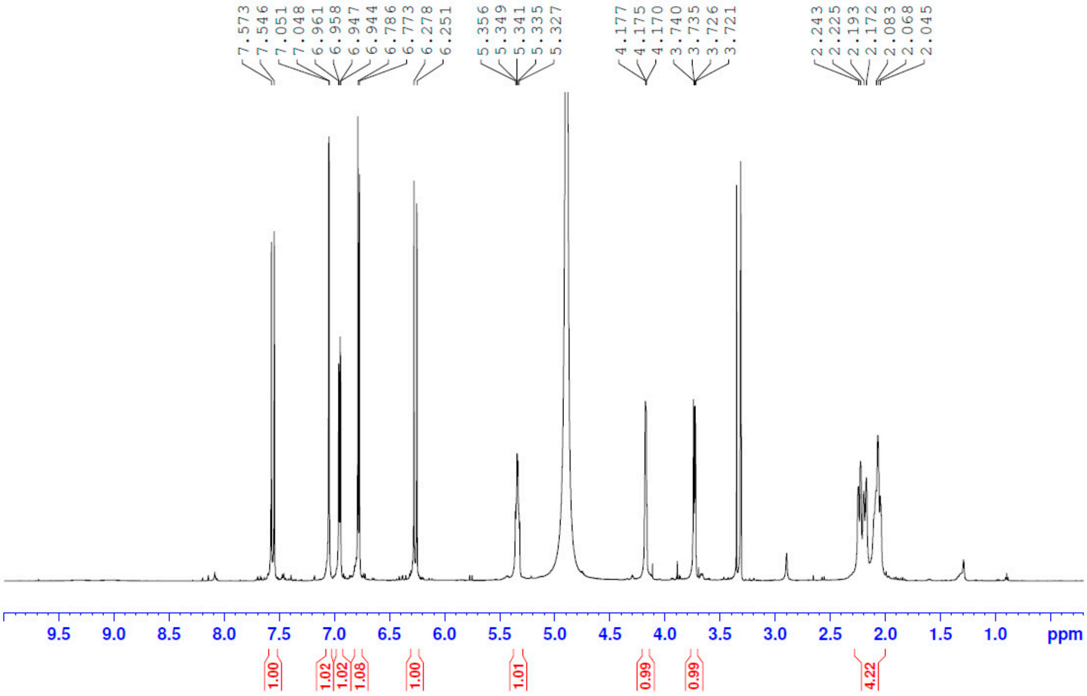

Figure S42. The ^{1}H-NMR (600MHz, CD_3OD) spectrum of compound 14

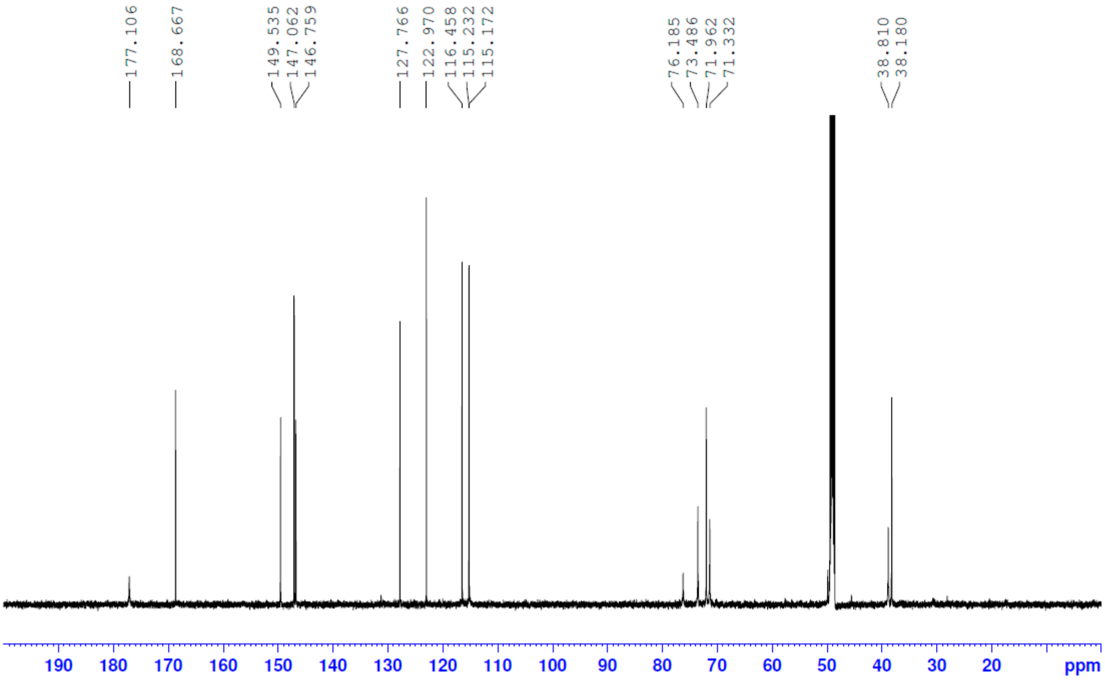

Figure S43. The ^{13}C-NMR (150MHz, CD_3OD) spectrum of compound 14

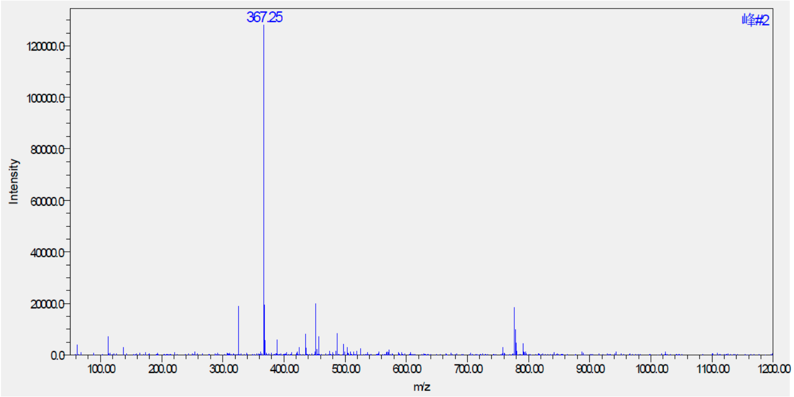

Figure S44. The ESI-MS spectrum of compound 15

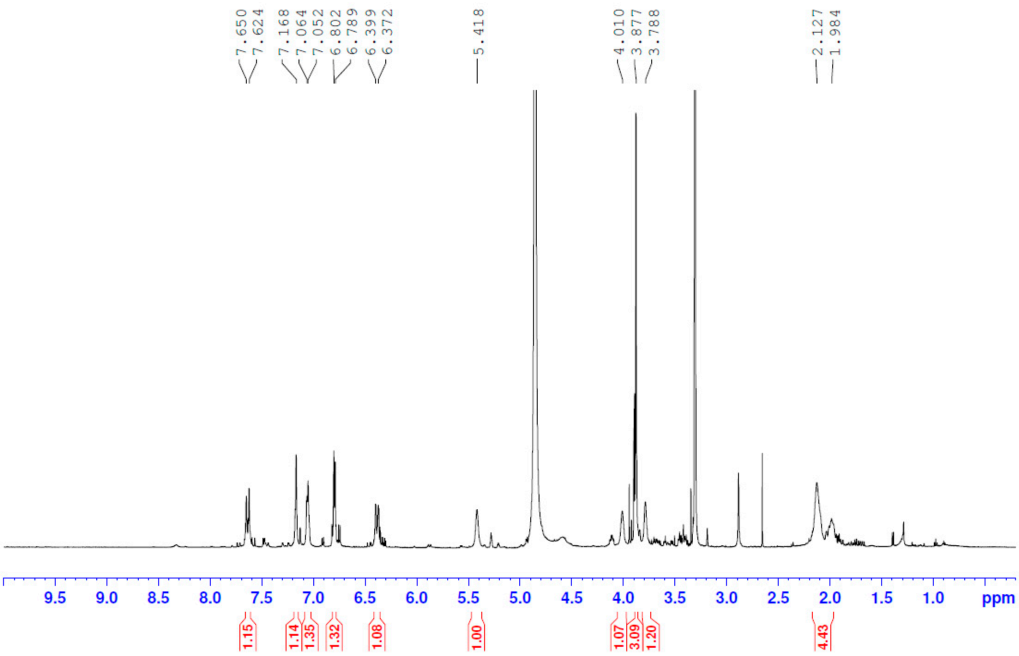

Figure S45. The ^{1}H-NMR (600MHz, CD_3OD) spectrum of compound 15

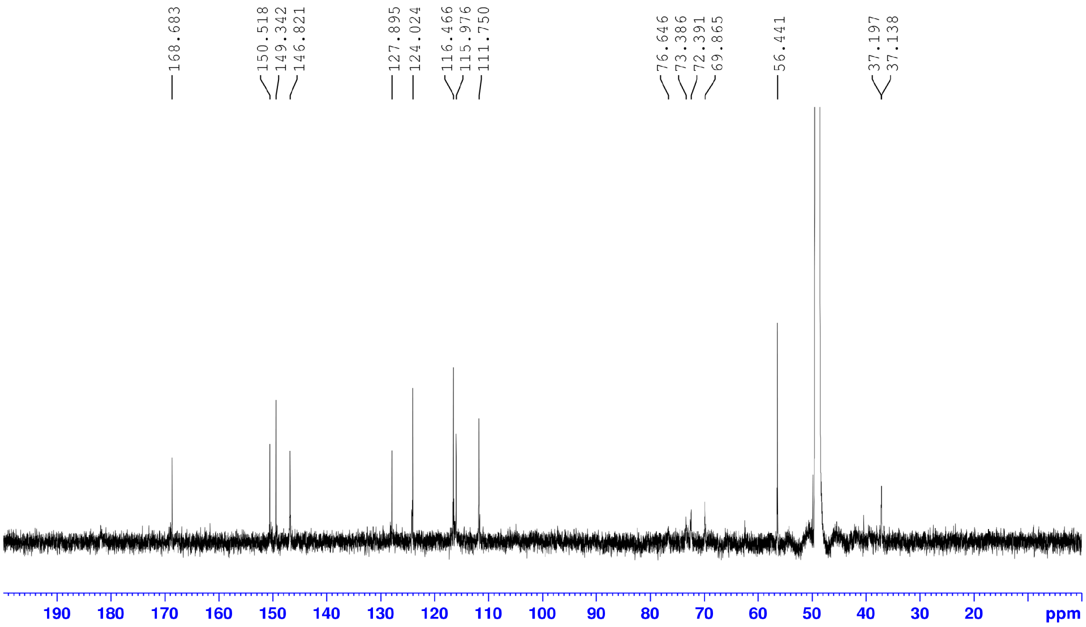

Figure S46. The ^{13}C-NMR (150MHz, CD_3OD) spectrum of compound 15

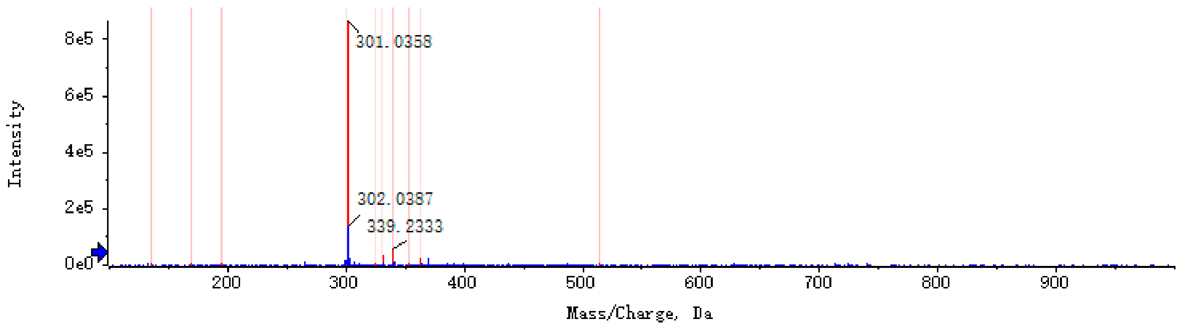

Figure S47. The HR-ESI-MS spectrum of compound 16

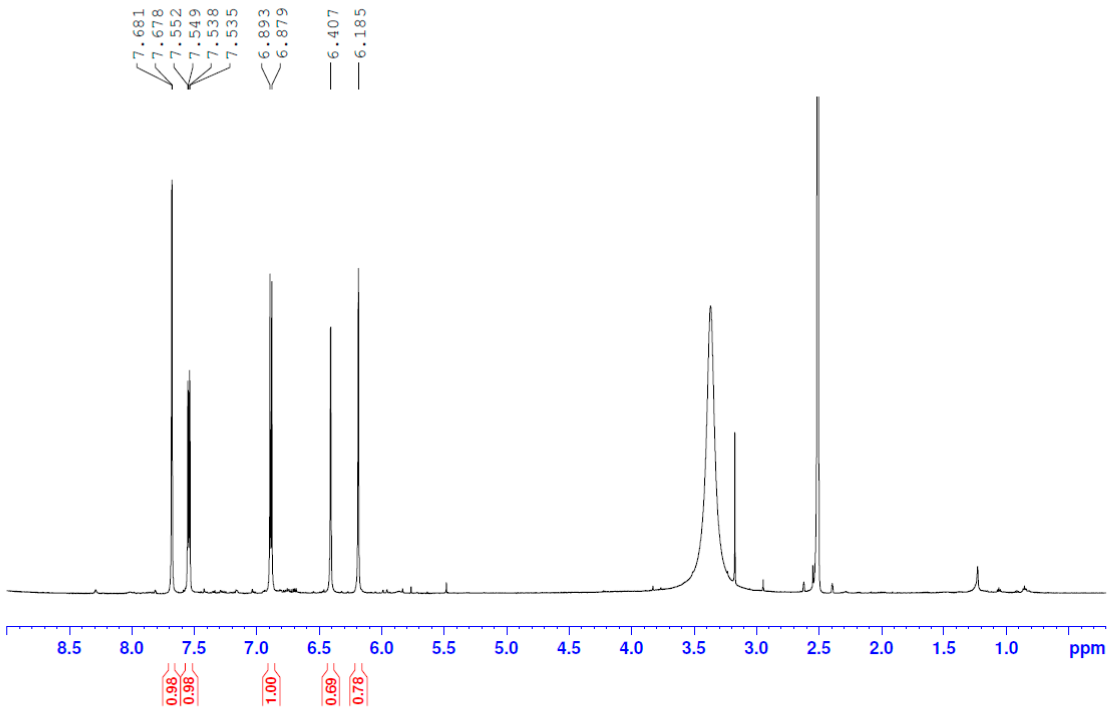

Figure S48. The ^{1}H-NMR (600MHz, DMSO-d_6) spectrum of compound 16

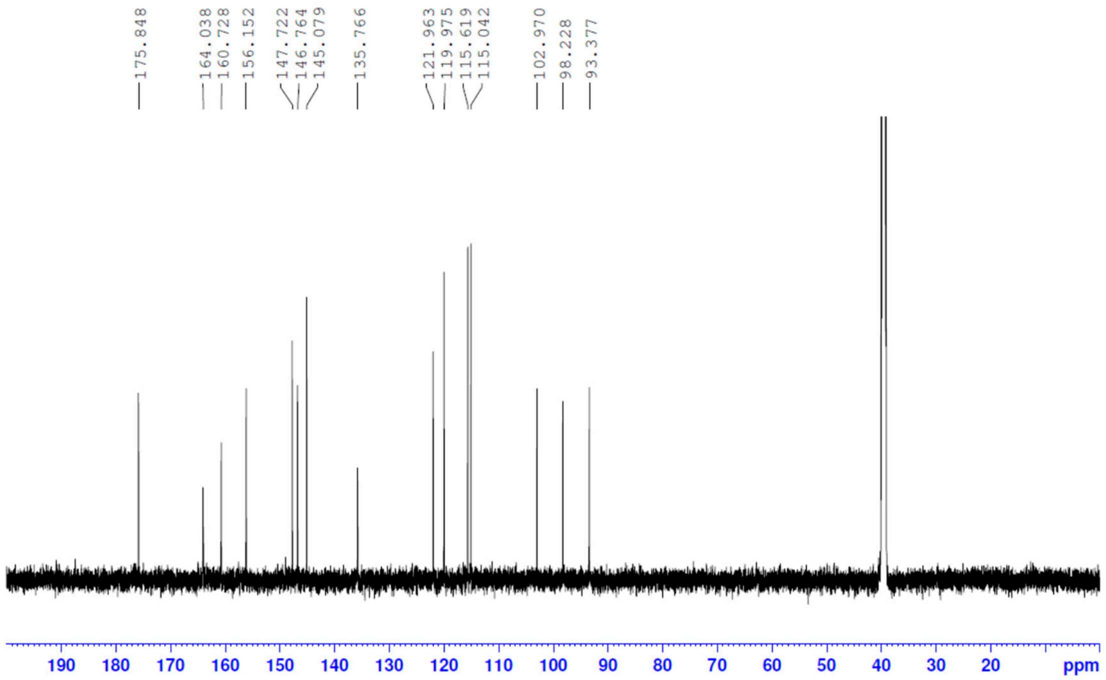

Figure S49. The ^{13}C-NMR (150MHz, DMSO-d_6) spectrum of compound 16

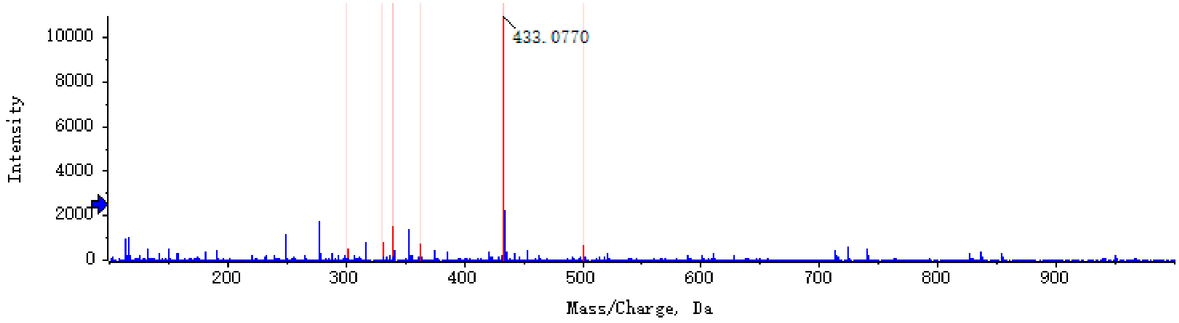

Figure S50. The HR-ESI-MS spectrum of compound 17

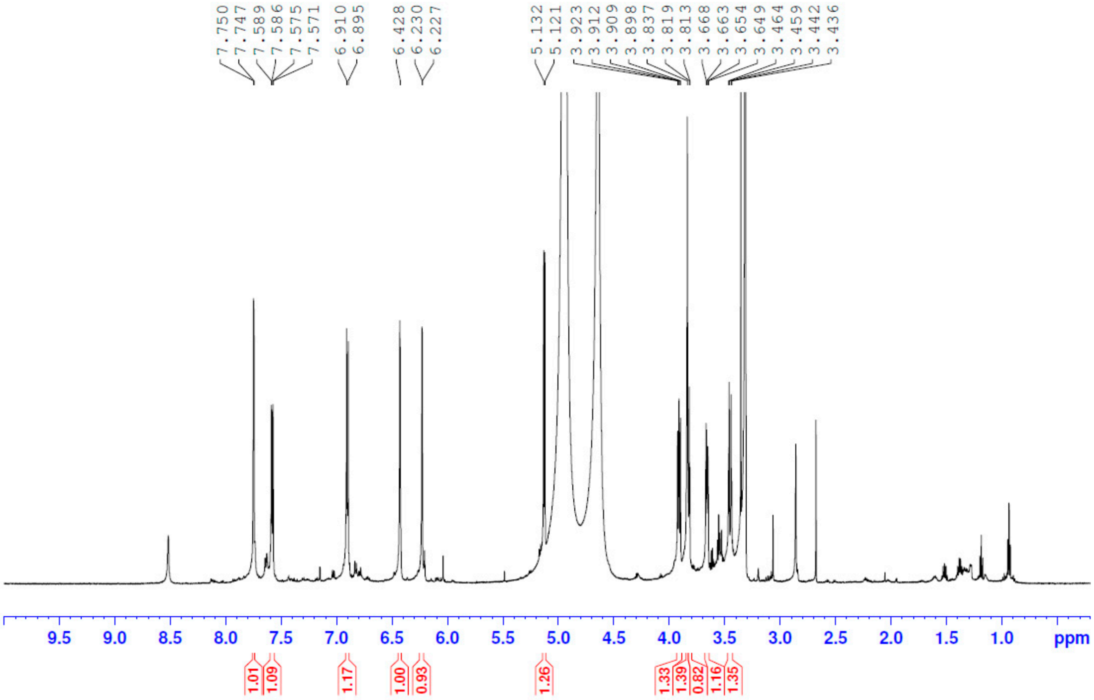

Figure S51. The ^{1}H-NMR (600MHz, CD_3OD) spectrum of compound 17

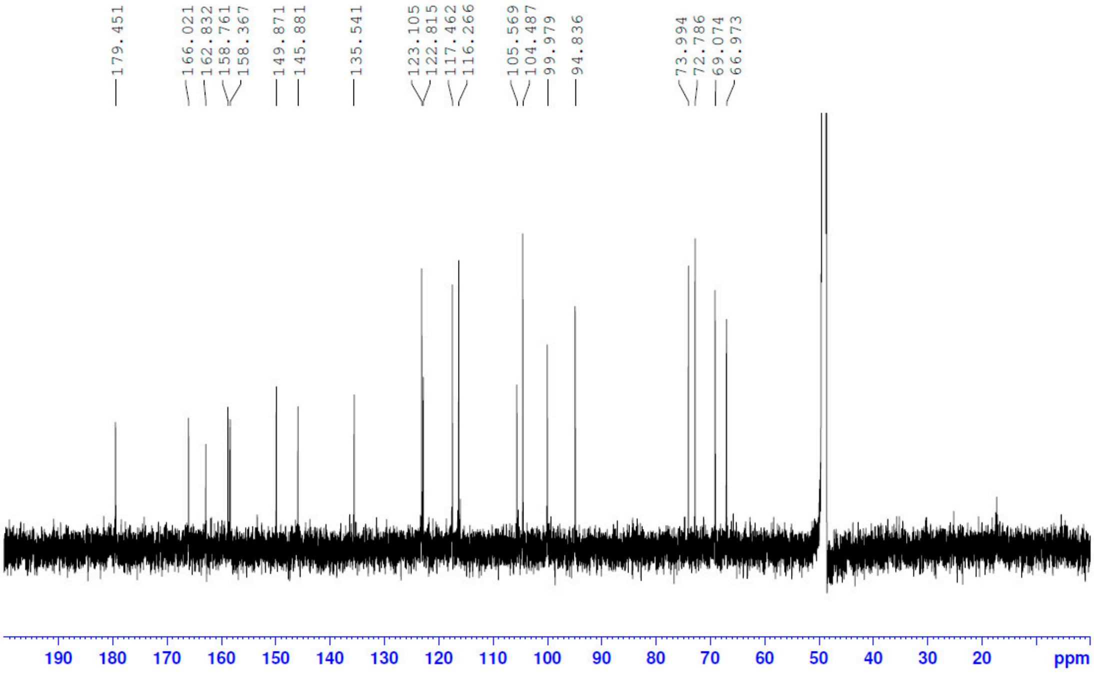

Figure S52. The ^{13}C-NMR (150MHz, CD_3OD) spectrum of compound 17

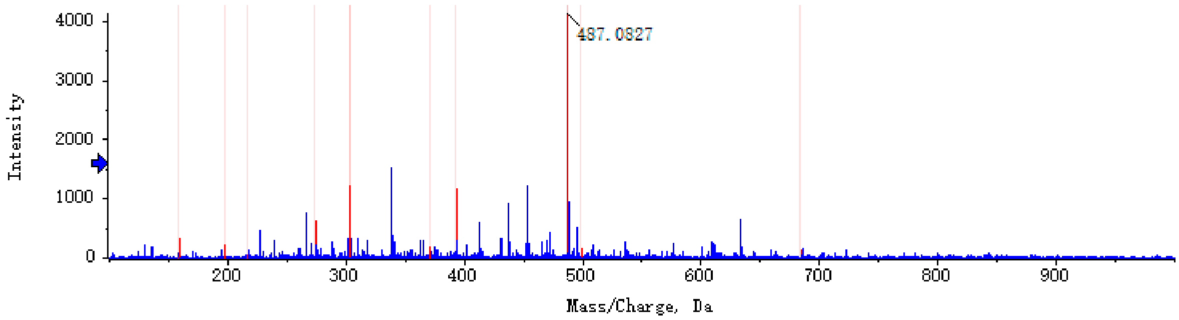

Figure S53. The HR-ESI-MS spectrum of compound 18

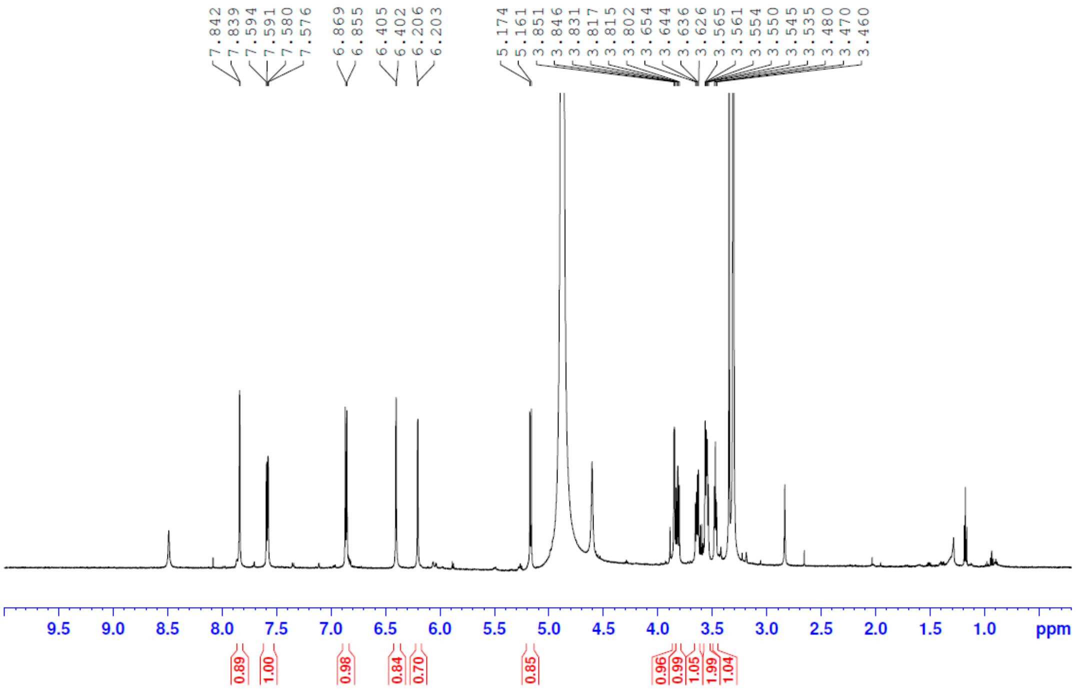

Figure S54. The ^{1}H-NMR (600MHz, CD_3OD) spectrum of compound 18

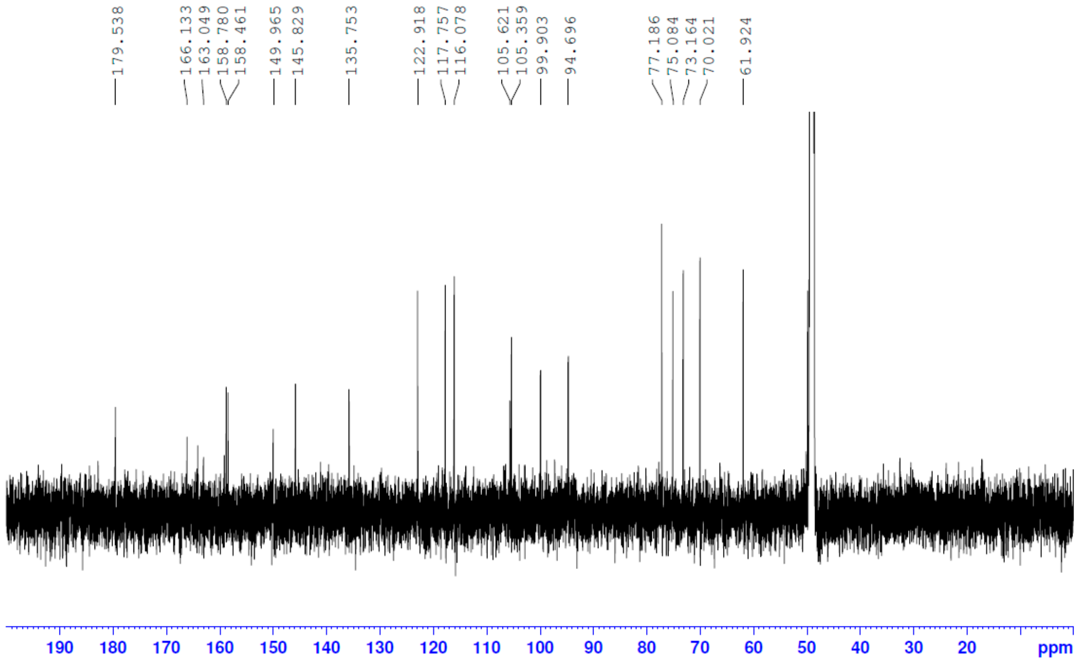

Figure S55. The ^{13}C-NMR (150MHz, CD_3OD) spectrum of compound 18

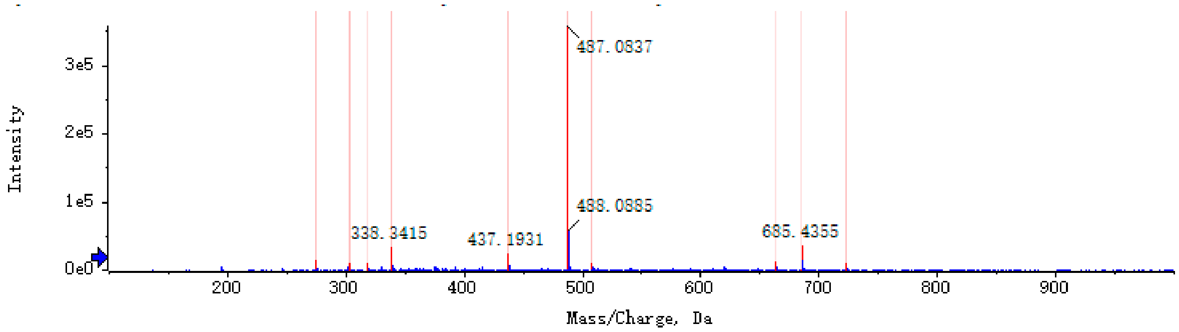

Figure S56. The HR-ESI-MS spectrum of compound 19

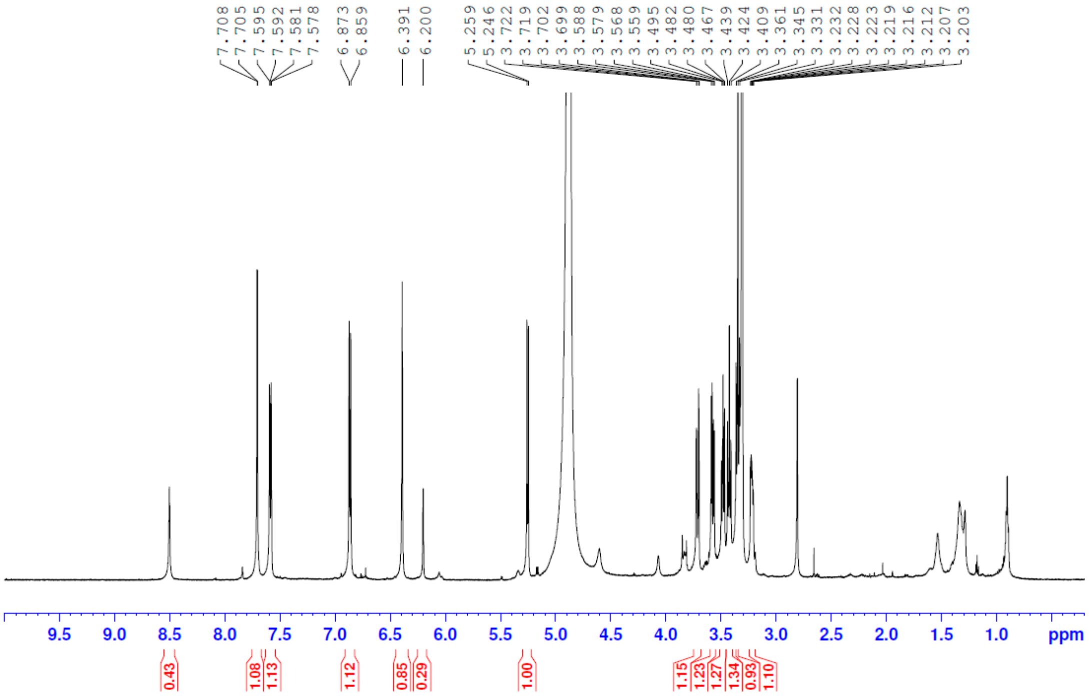
Figure S57. The ^{1}H-NMR (600MHz, CD_3OD) spectrum of compound 19

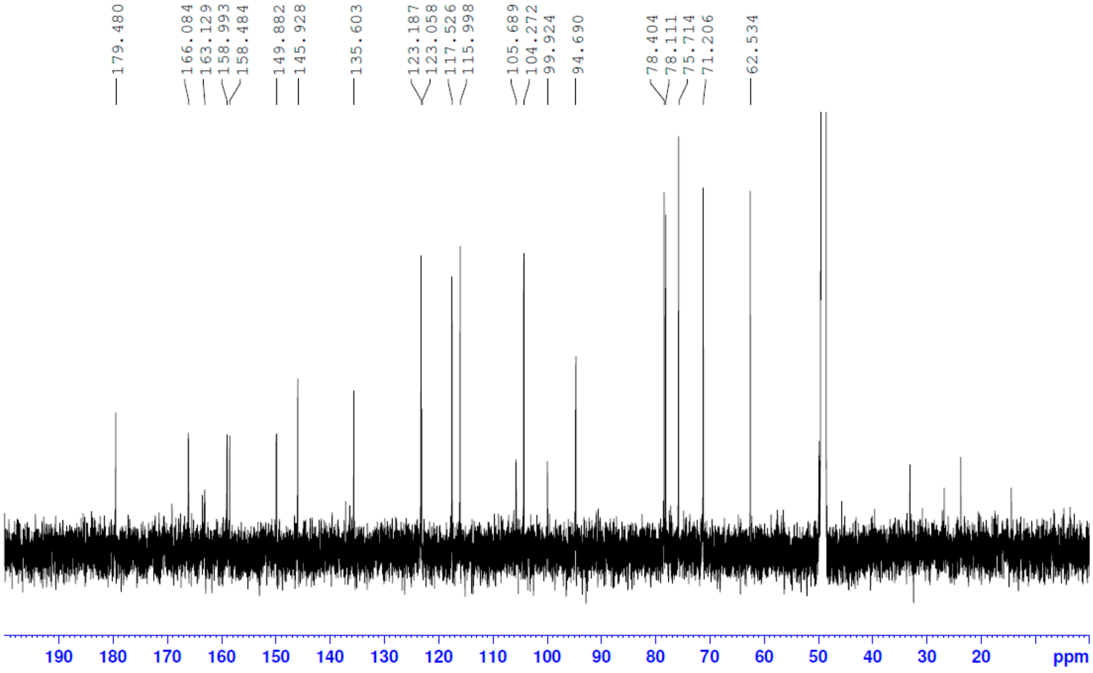
Figure S58. The ^{13}C-NMR (150MHz, CD_3OD) spectrum of compound 19

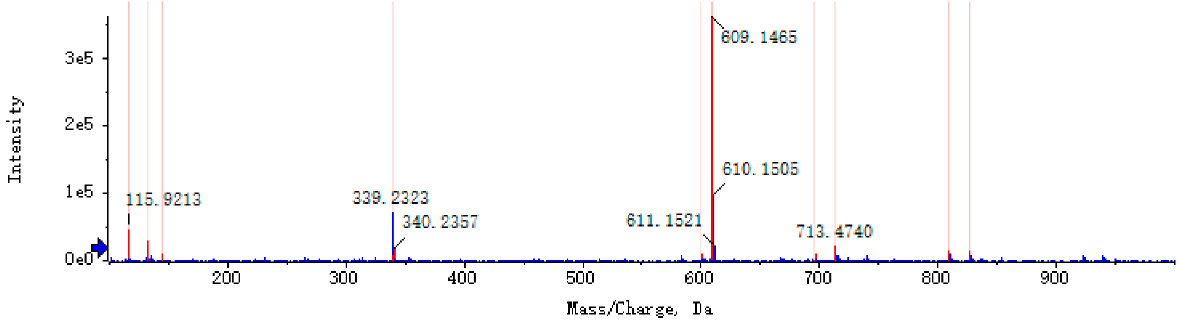

Figure S59. The HR-ESI-MS spectrum of compound 20

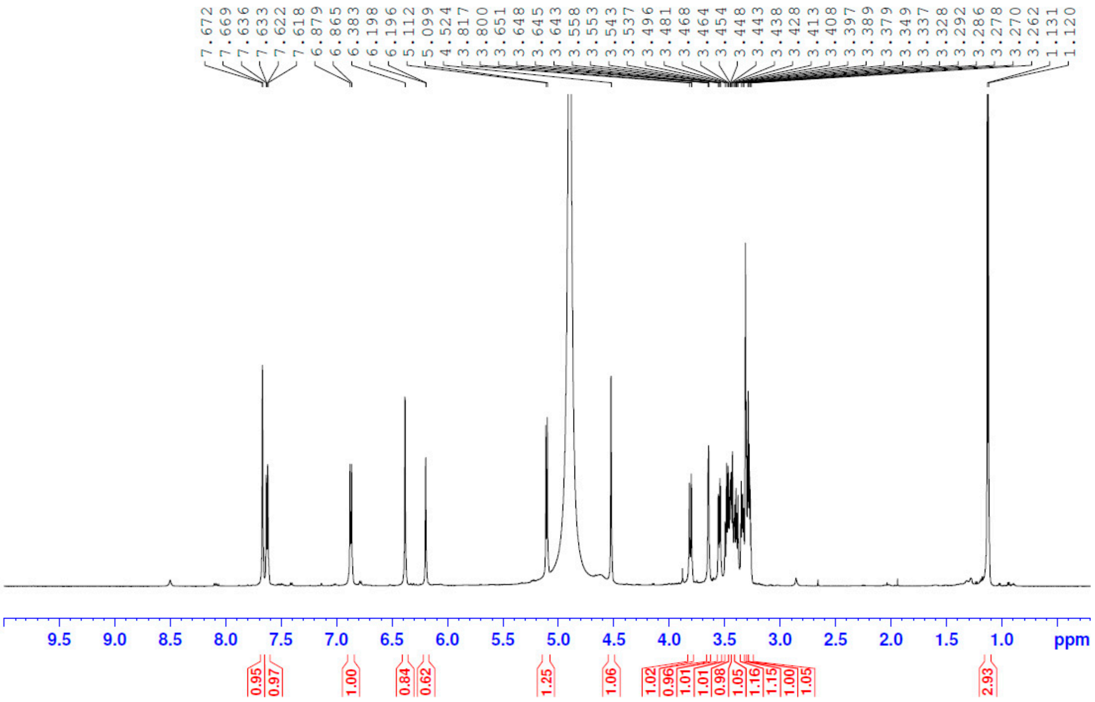

Figure S60. The ^{1}H-NMR (600MHz, CD_3OD) spectrum of compound 20

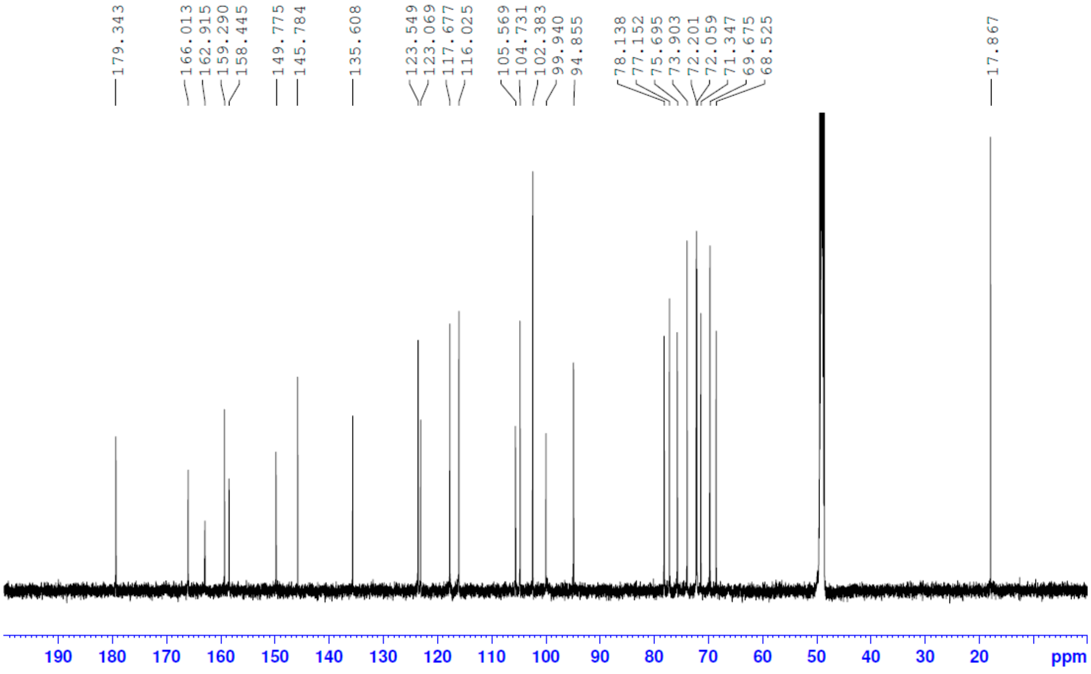

Figure S61. The ^{13}C-NMR (150MHz, CD_3OD) spectrum of compound 20

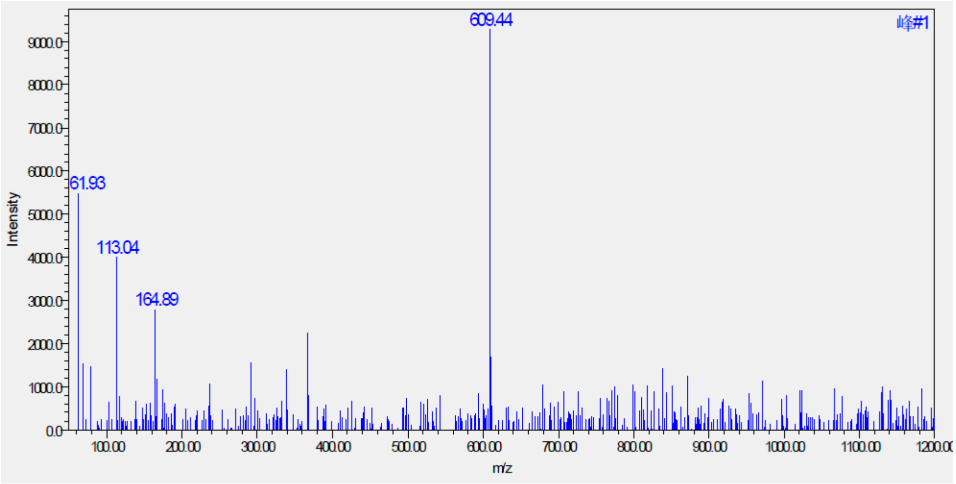

Figure S62. The ESI-MS spectrum of compound 21

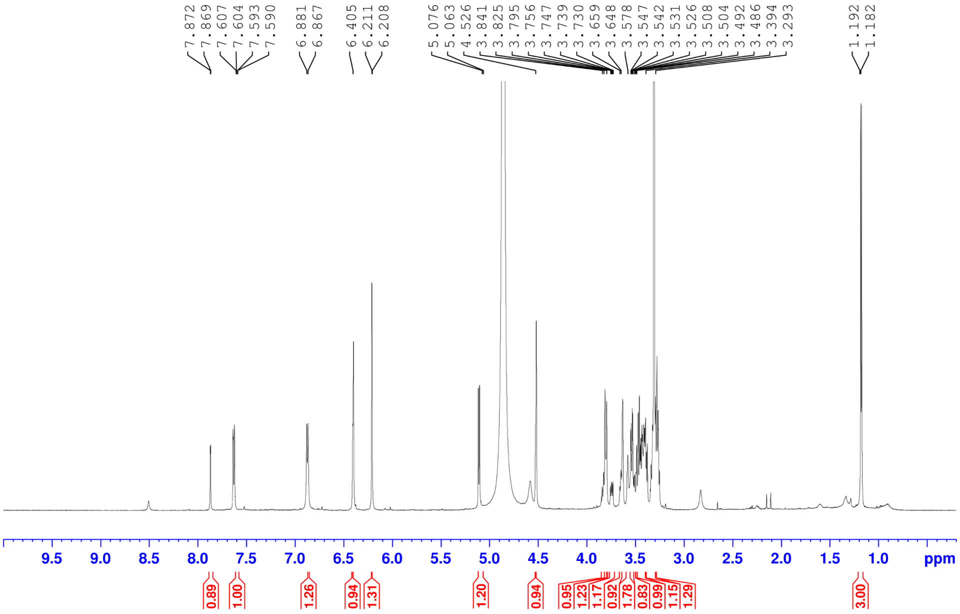

Figure S63. The ^{1}H-NMR (600MHz, CD_3OD) spectrum of compound 21

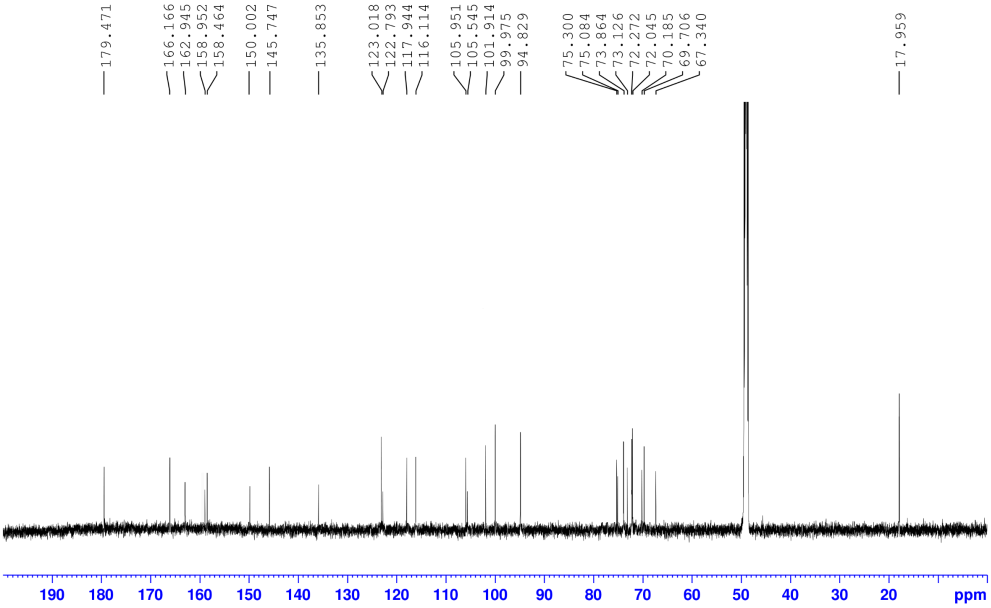

Figure S64. The ^{13}C-NMR (150MHz, CD_3OD) spectrum of compound 21

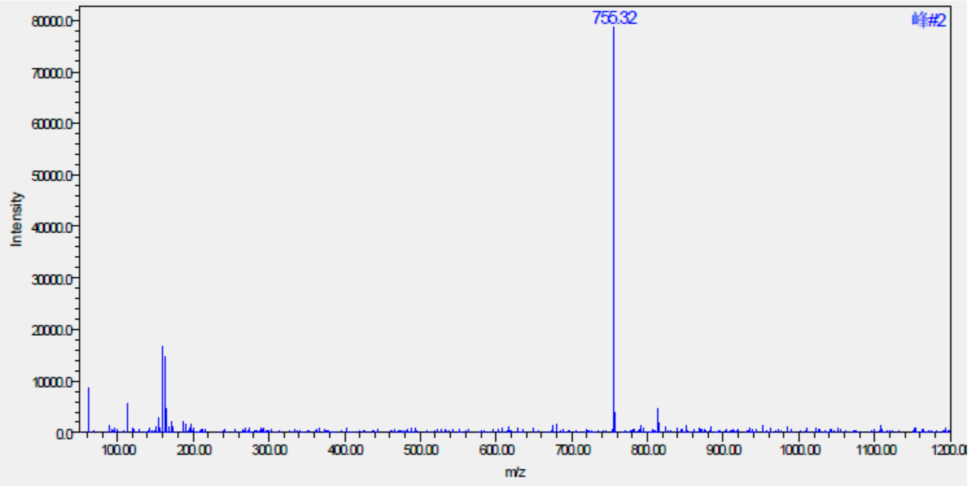

Figure S65. The ESI-MS spectrum of compound 22

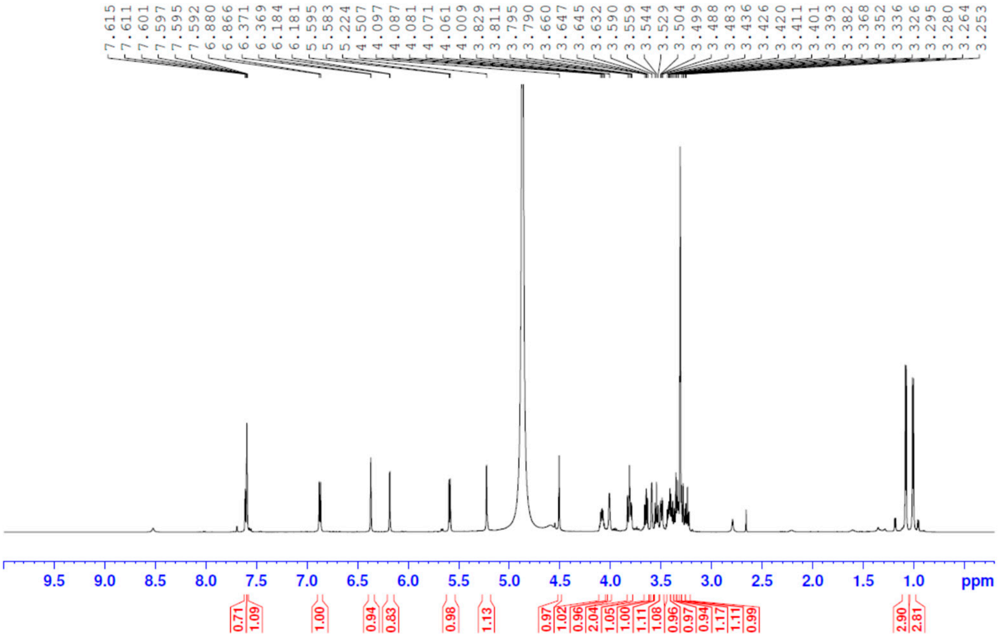

Figure S66. The ^{1}H-NMR (600MHz, CD_3OD) spectrum of compound 22

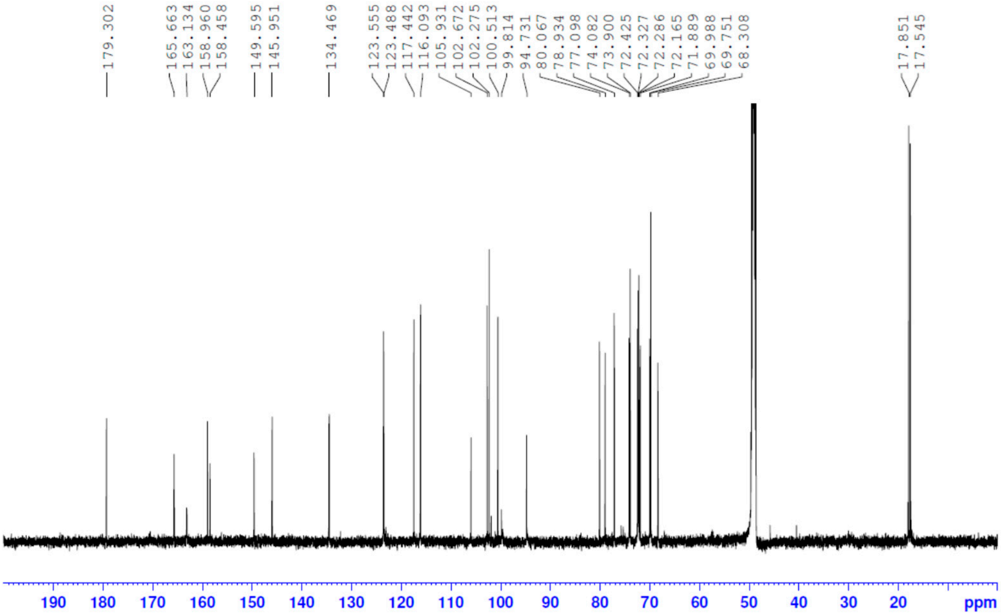

Figure S67. The ^{13}C-NMR (150MHz, CD_3OD) spectrum of compound 22

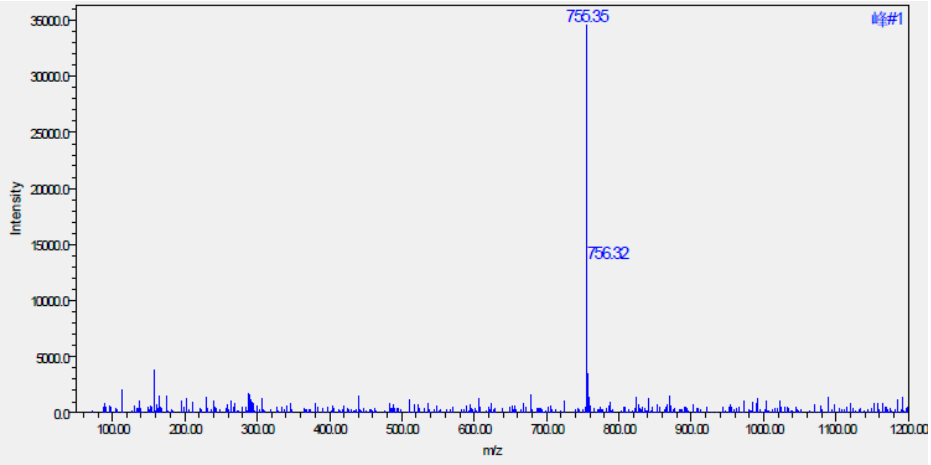

Figure S68. The ESI-MS spectrum of compound 23

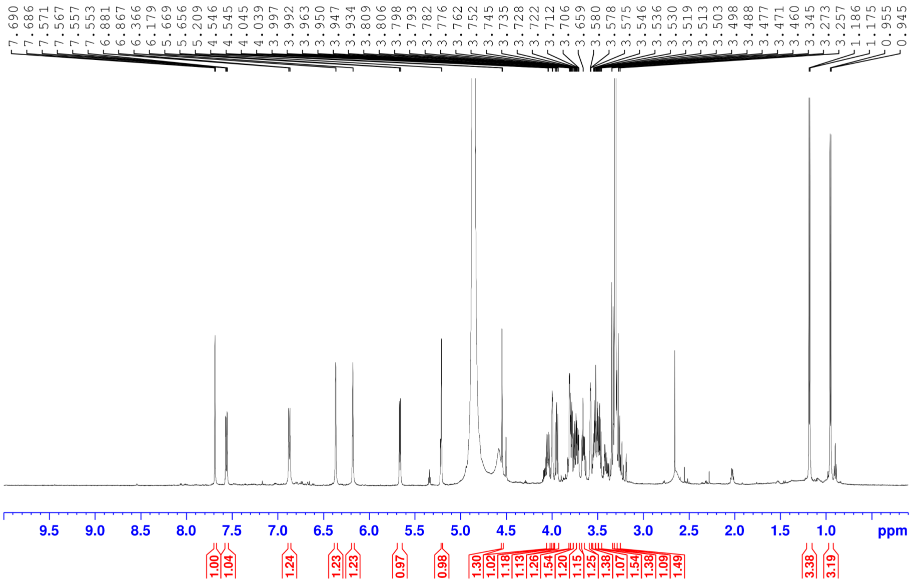

Figure S69. The ^{1}H-NMR (600MHz, CD_3OD) spectrum of compound 23

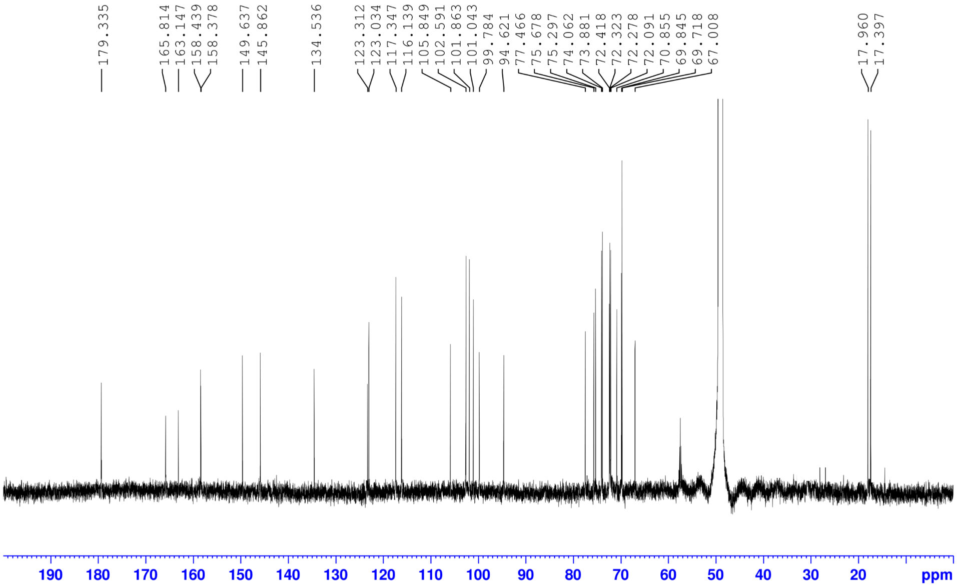

Figure S70. The ^{13}C-NMR (150MHz, CD_3OD) spectrum of compound 23

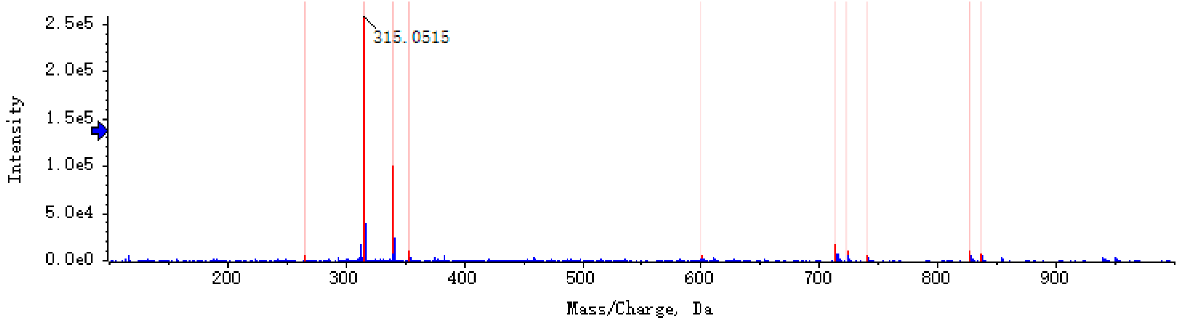

Figure S71. The ESI-MS spectrum of compound 24

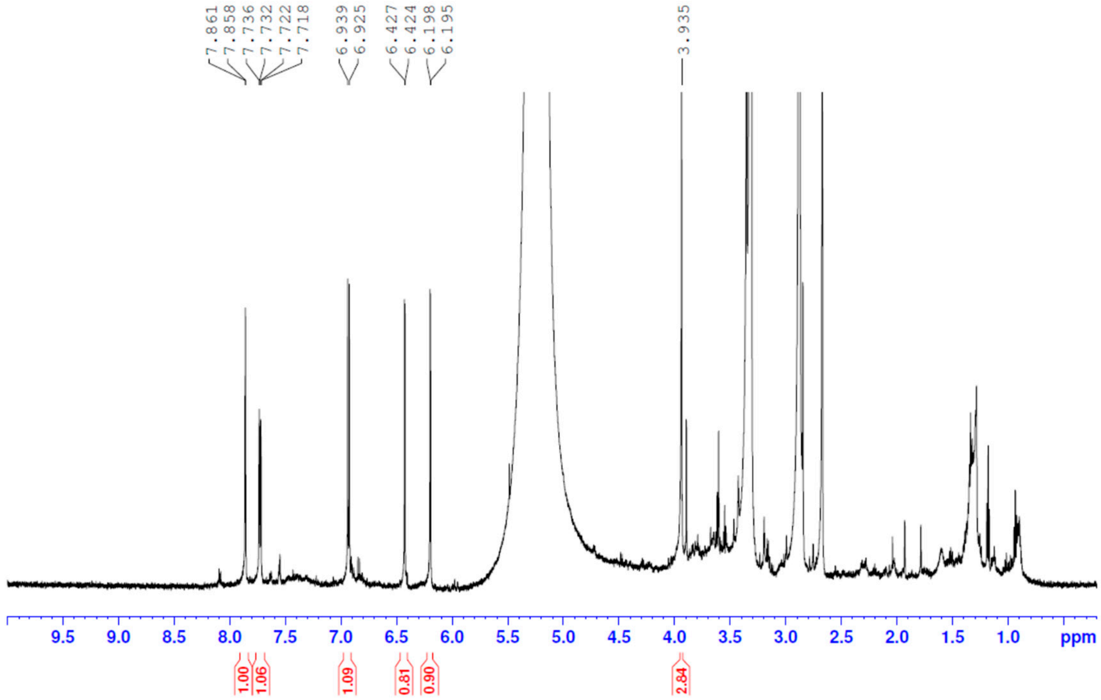

Figure S72. The ^{1}H-NMR (600MHz, CD_3OD) spectrum of compound 24

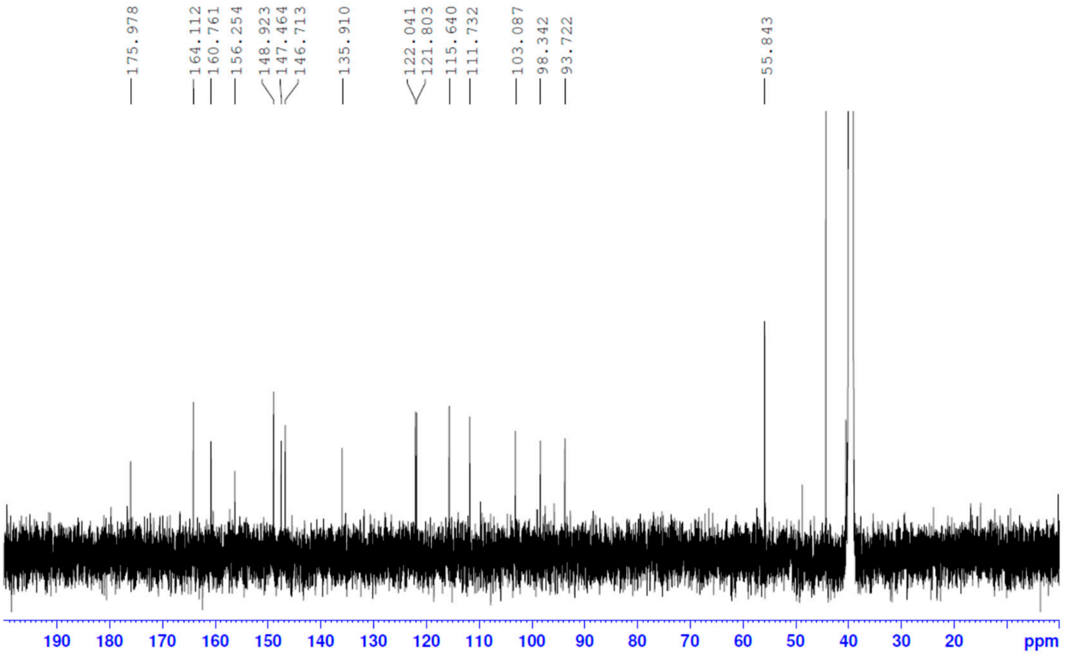

Figure S73. The ^{13}C-NMR (150MHz, DMSO-d_6) spectrum of compound 24

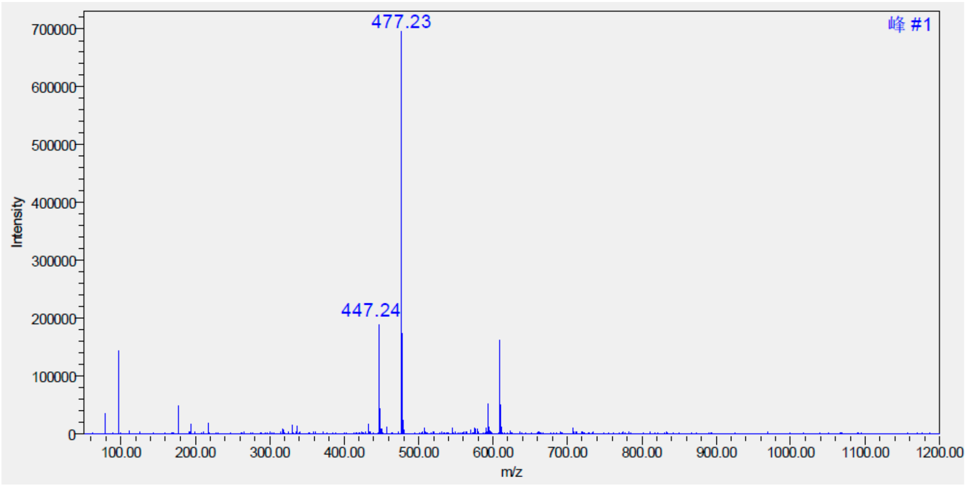

Figure S74. The ESI-MS spectrum of compound 25

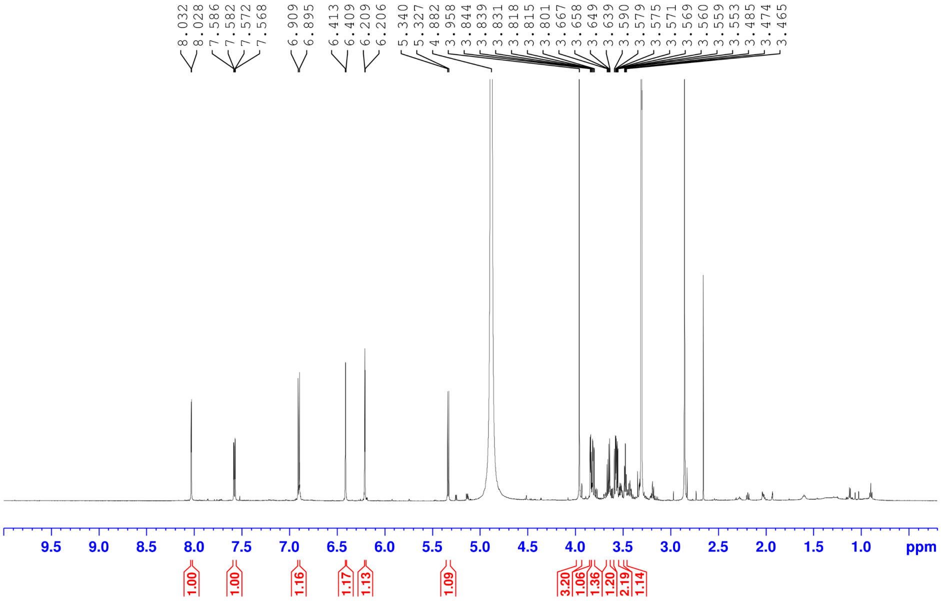

Figure S75. The ^{1}H-NMR (600MHz, CD_3OD) spectrum of compound 25

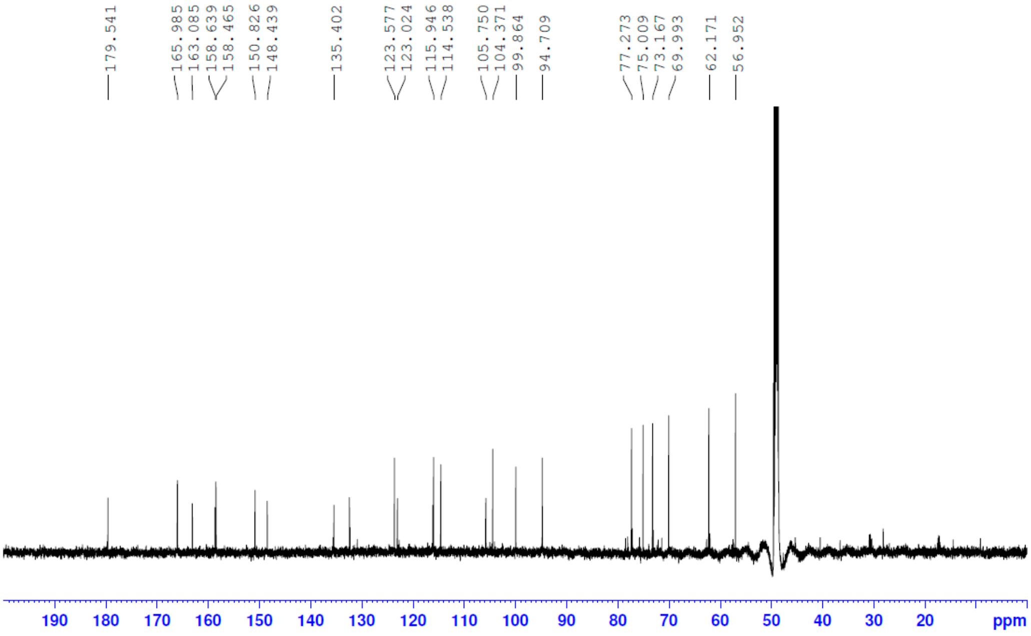

Figure S76. The ^{13}C-NMR (150MHz, CD_3OD) spectrum of compound 25

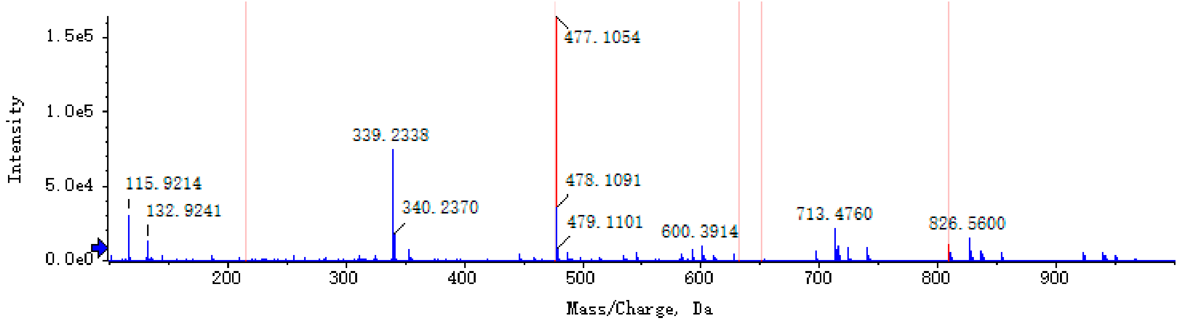

Figure S77. The ESI-MS spectrum of compound 26

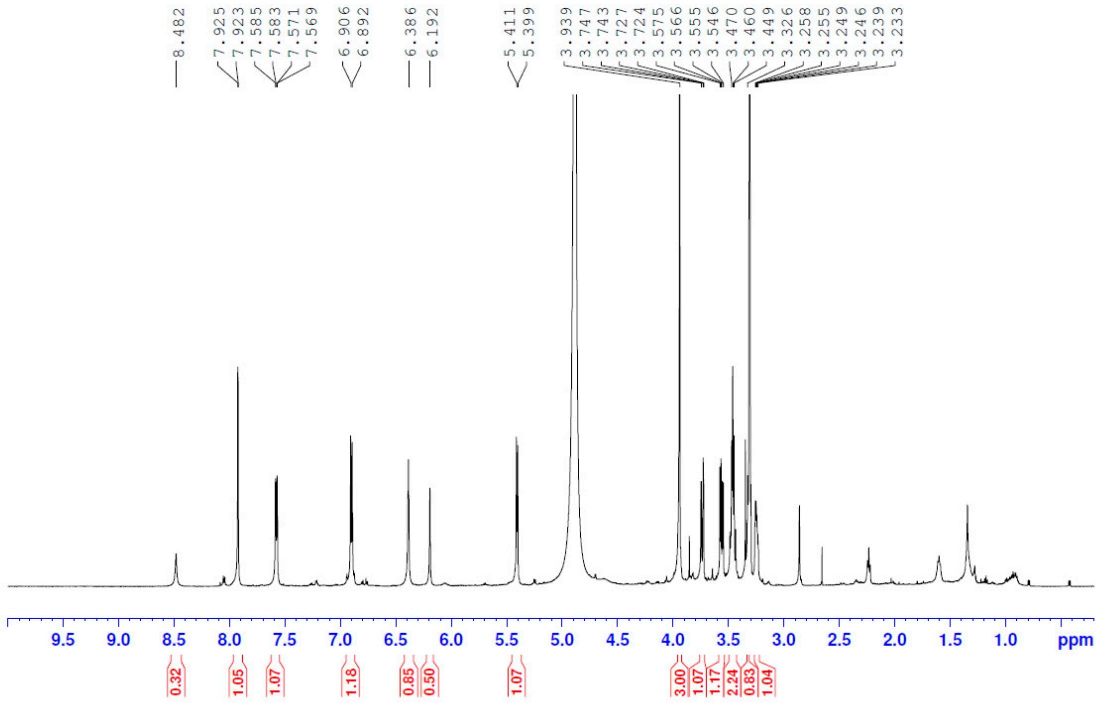

Figure S78. The ^{1}H-NMR (600MHz, CD_3OD) spectrum of compound 26

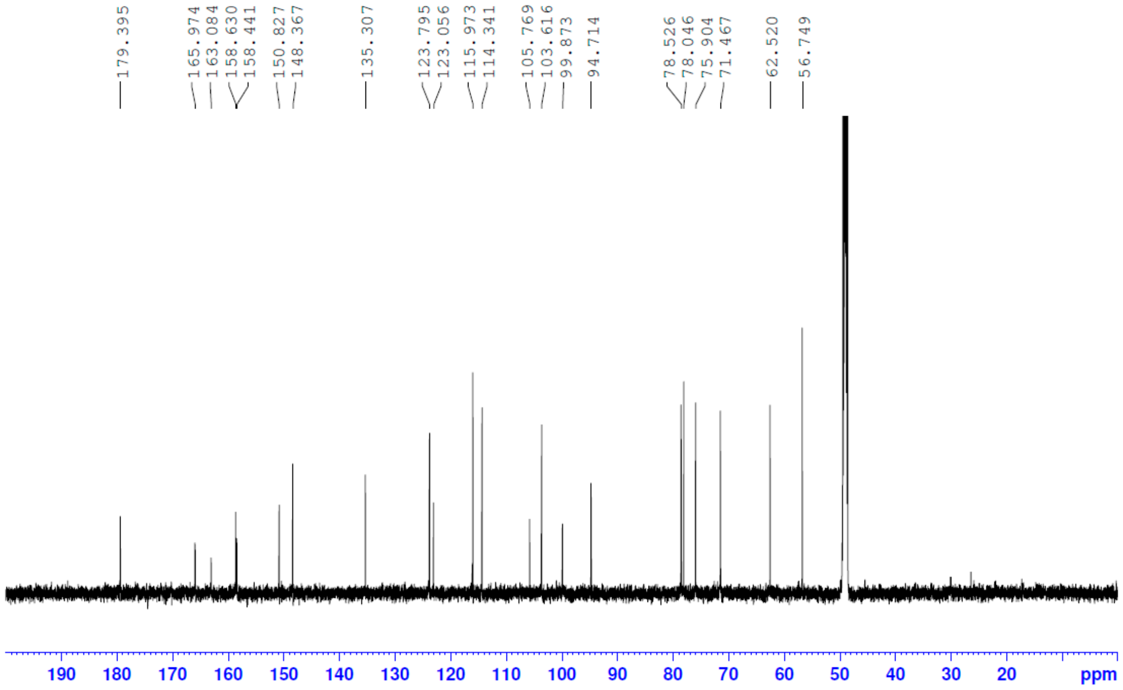

Figure S79. The ^{13}C-NMR (150MHz, CD_3OD) spectrum of compound 26

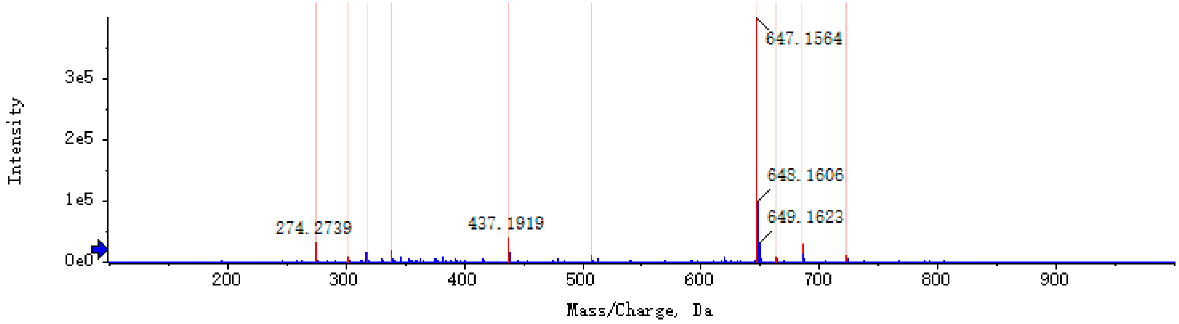

Figure S80. The ESI-MS spectrum of compound 27

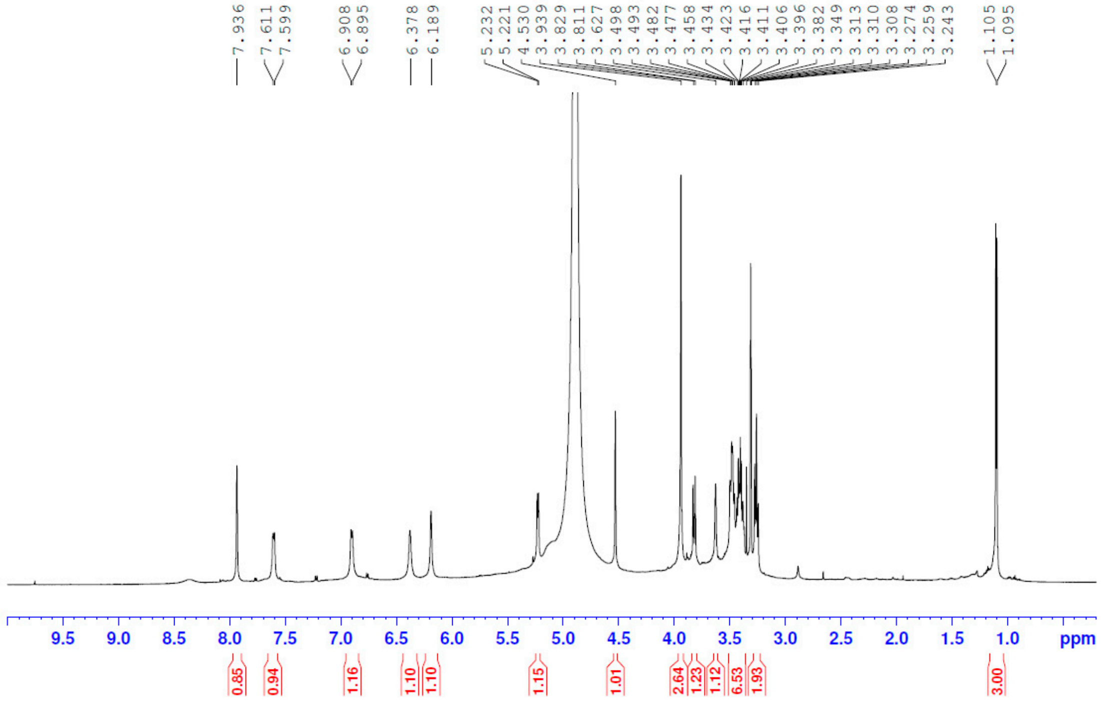

Figure S81. The ^{1}H-NMR (600MHz, CD_3OD) spectrum of compound 27

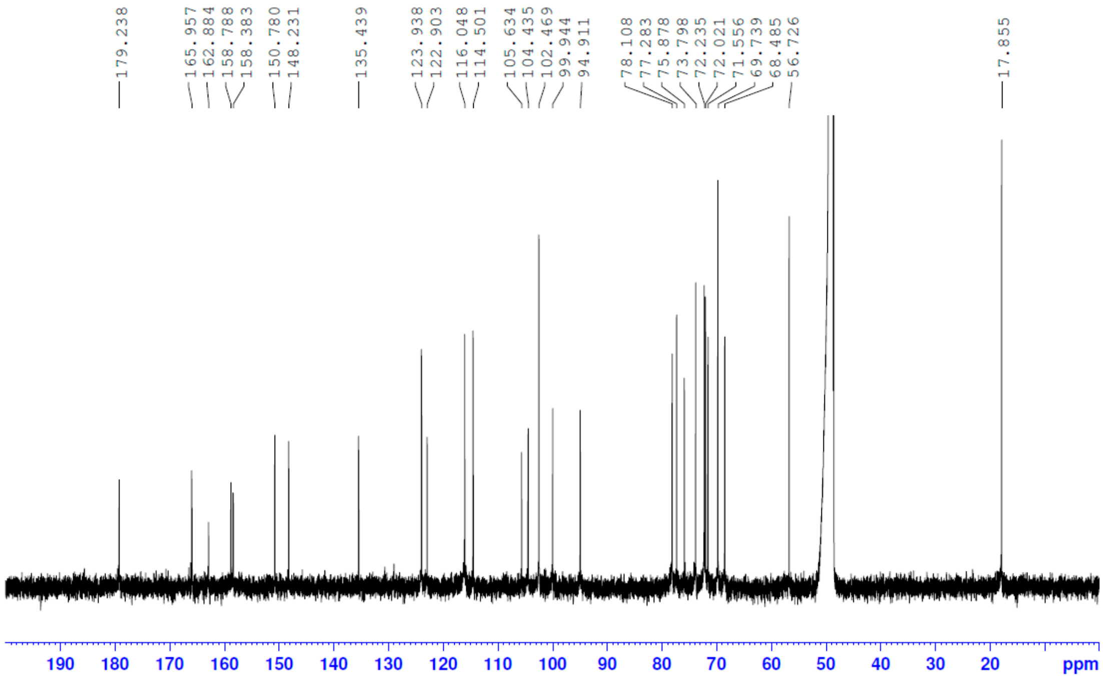

Figure S82. The ^{13}C-NMR (150MHz, CD_3OD) spectrum of compound 27

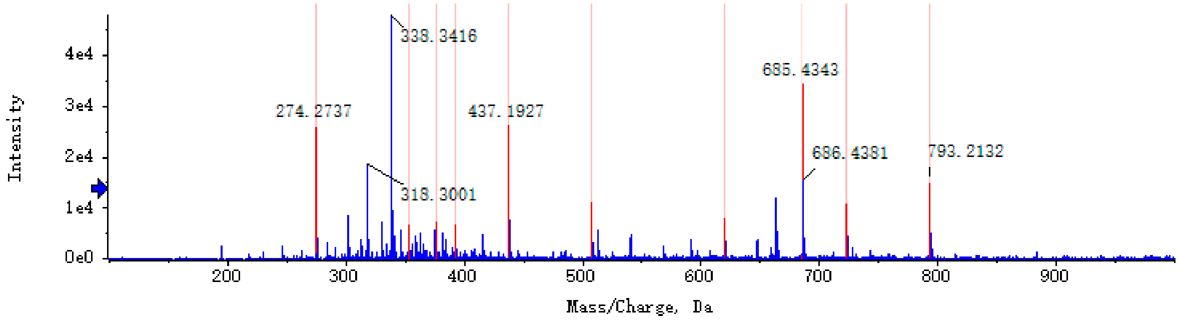

Figure S83. The HR-ESI-MS spectrum of compound 28

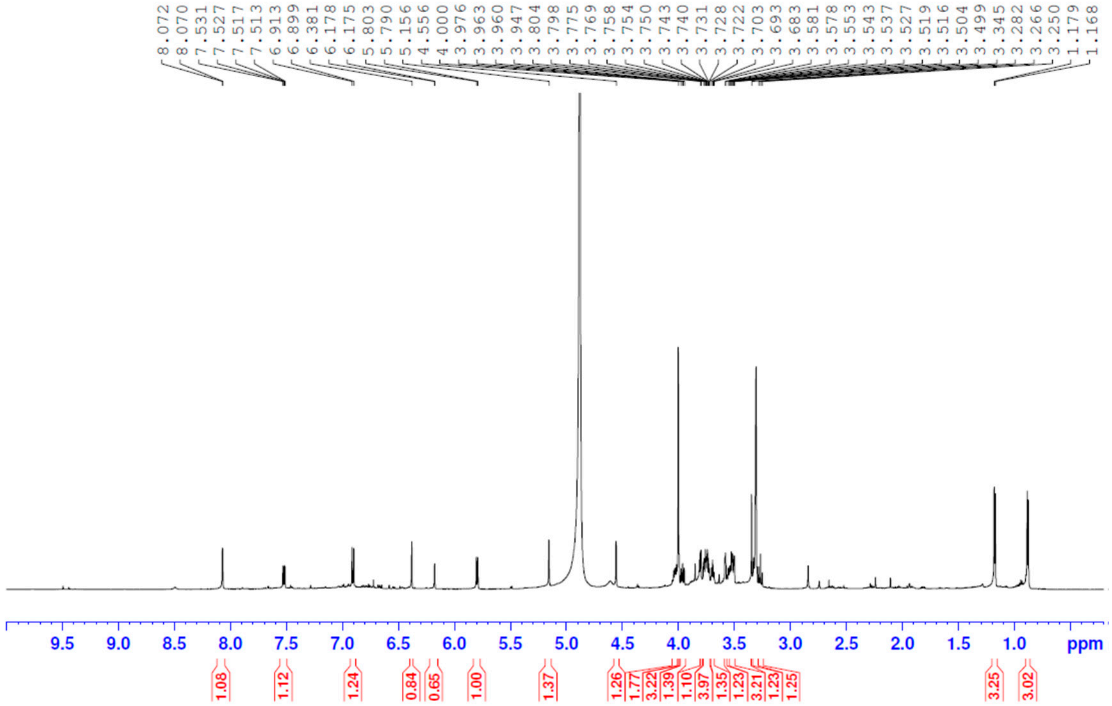

Figure S84. The ^{1}H-NMR (600MHz, CD_3OD) spectrum of compound 28

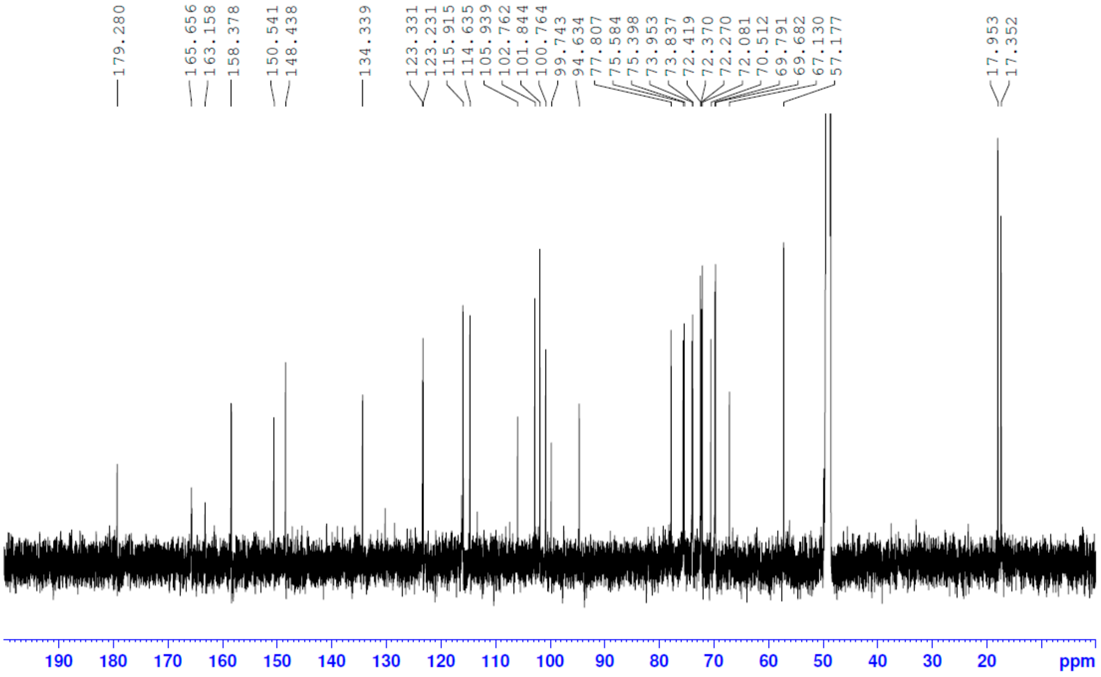

Figure S85. The ^{13}C-NMR (150MHz, CD_3OD) spectrum of compound 28

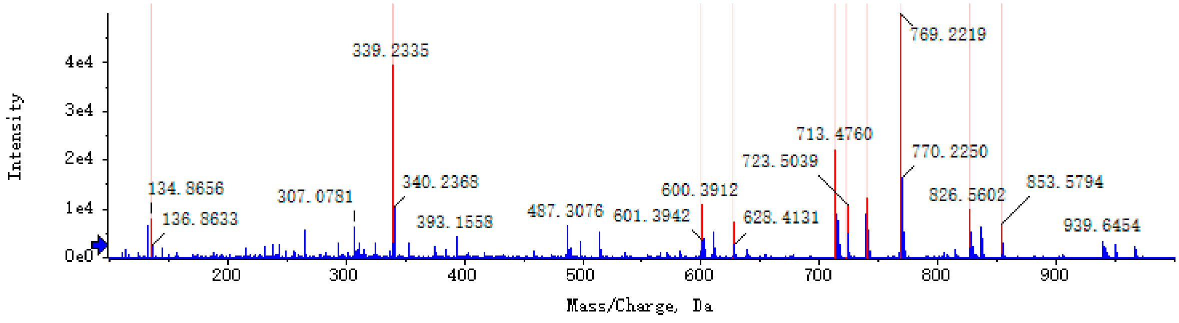

Figure S86. The HR-ESI-MS spectrum of compound 29

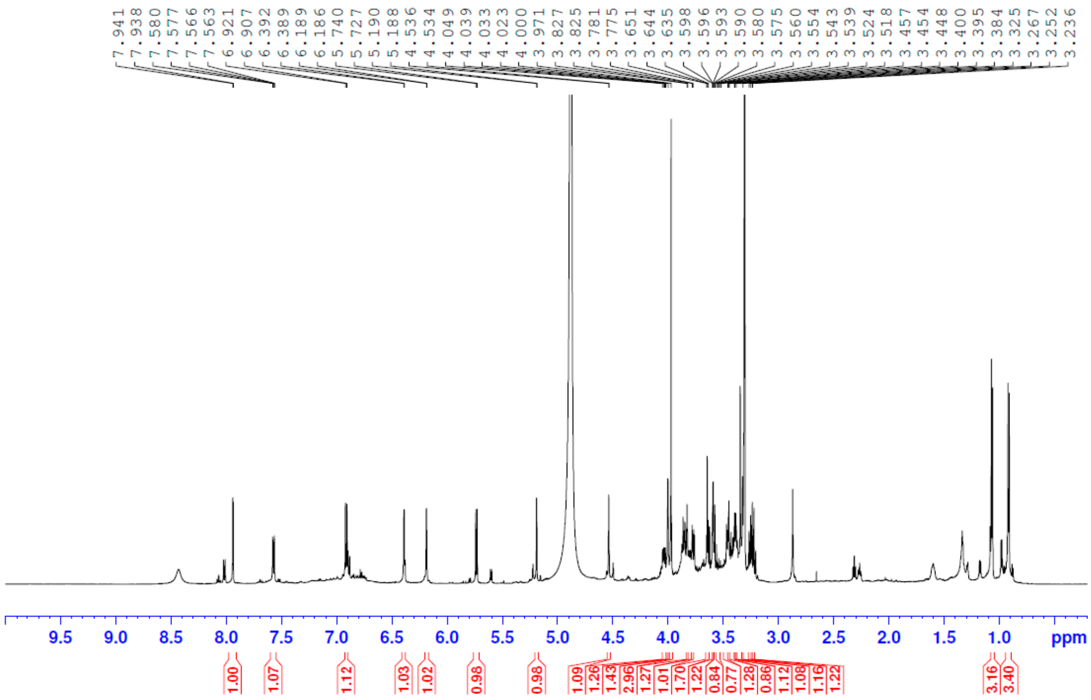

Figure S87. The ^{1}H-NMR (600MHz, CD_3OD) spectrum of compound 29

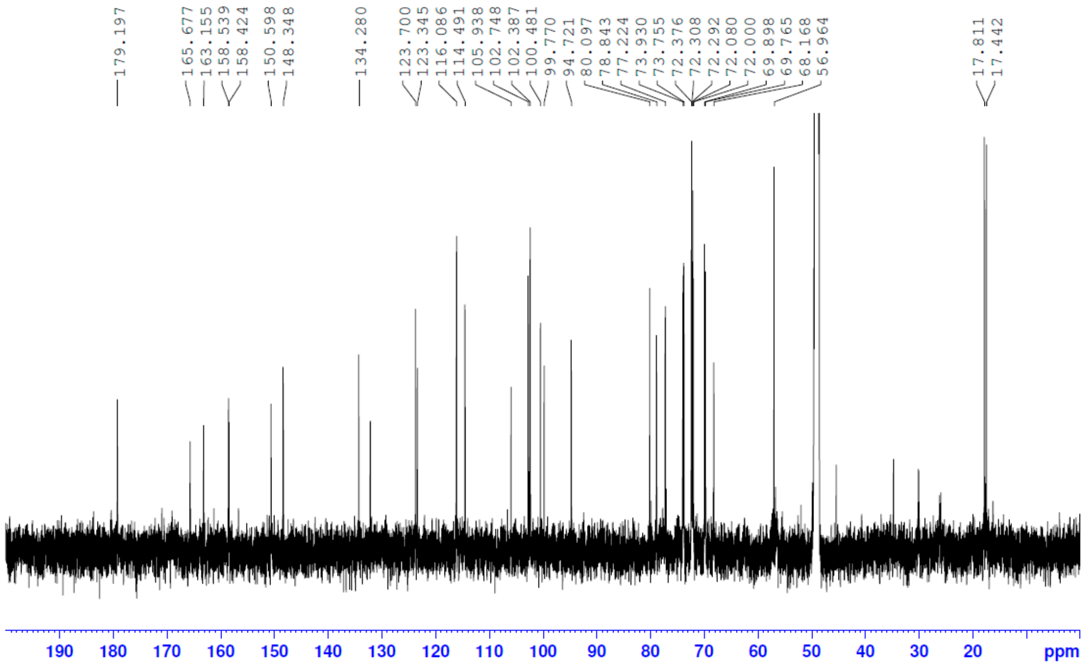

Figure S88. The ^{13}C-NMR (150MHz, CD_3OD) spectrum of compound 29

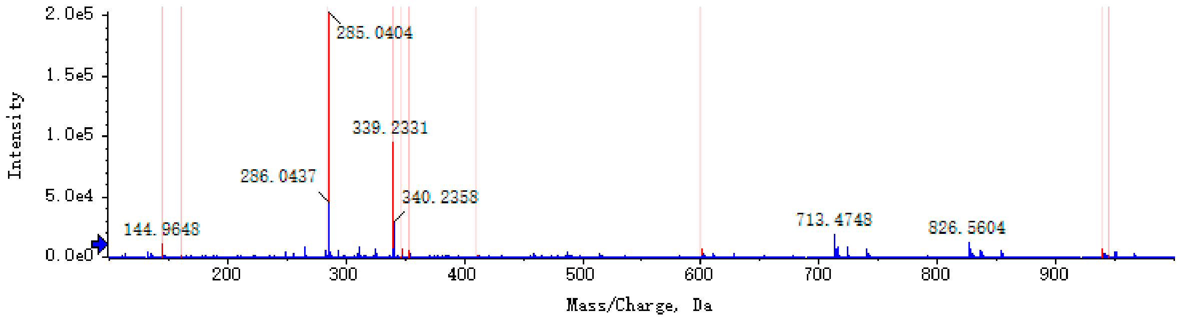

Figure S89. The HR-ESI-MS spectrum of compound 30

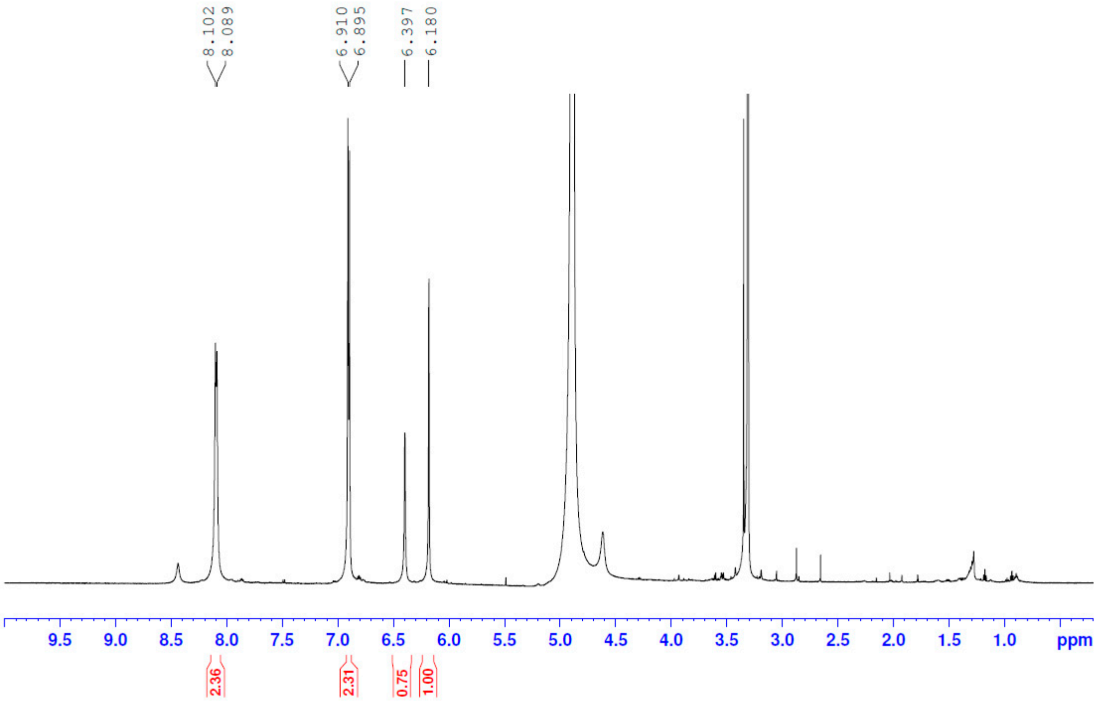

Figure S90. The ^{1}H-NMR (600MHz, CD_3OD) spectrum of compound 30

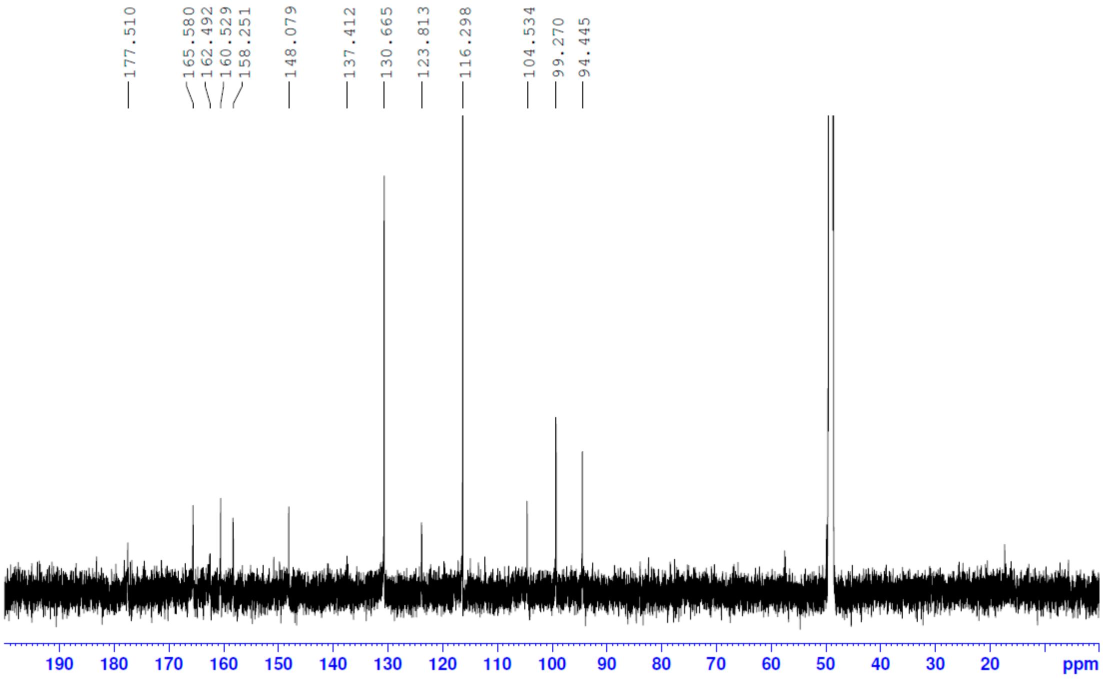

Figure S91. The ^{13}C-NMR (150MHz, CD_3OD) spectrum of compound 30

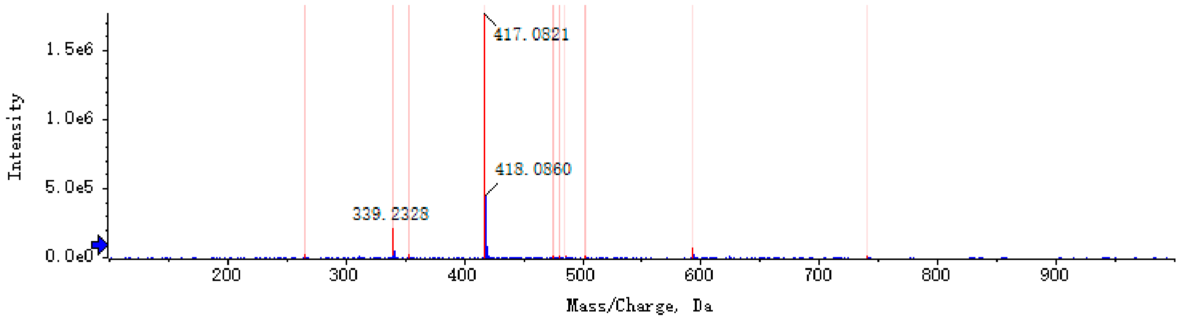

Figure S92. The HR-ESI-MS spectrum of compound 31

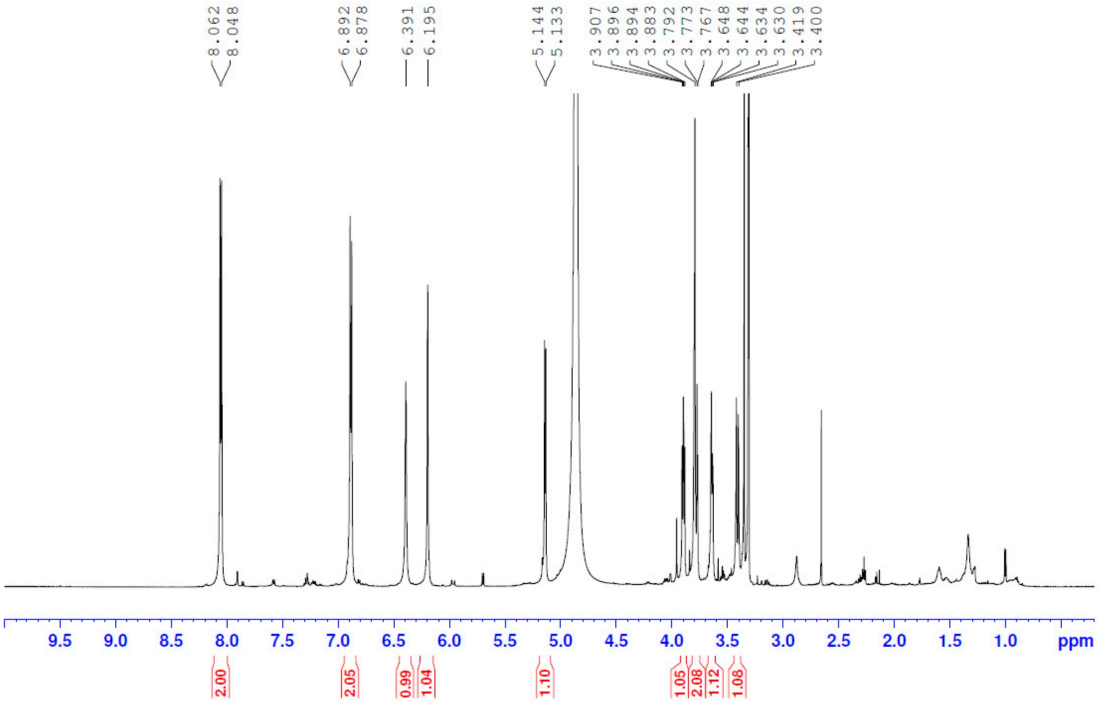

Figure S93. The ^{1}H-NMR (600MHz, CD_3OD) spectrum of compound 31

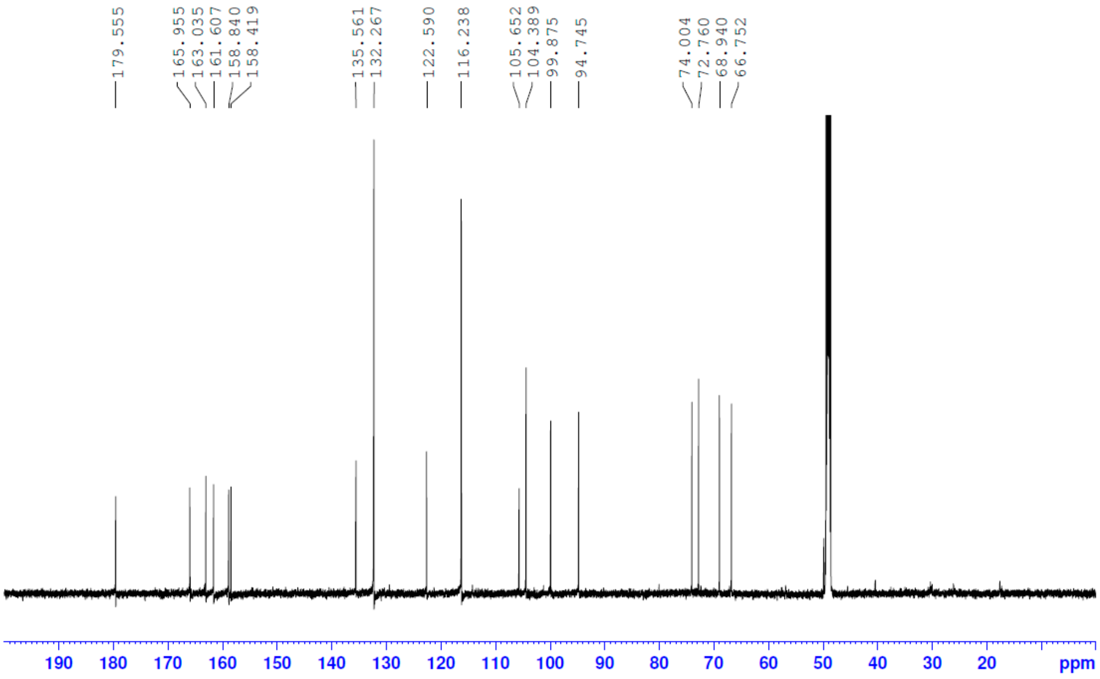

Figure S94. The ^{13}C-NMR (150MHz, CD_3OD) spectrum of compound 31

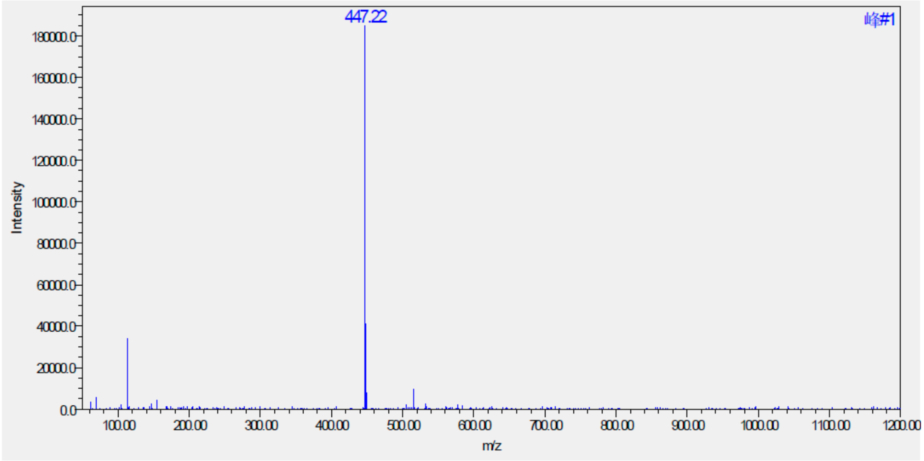

Figure S95. The ESI-MS spectrum of compound 32

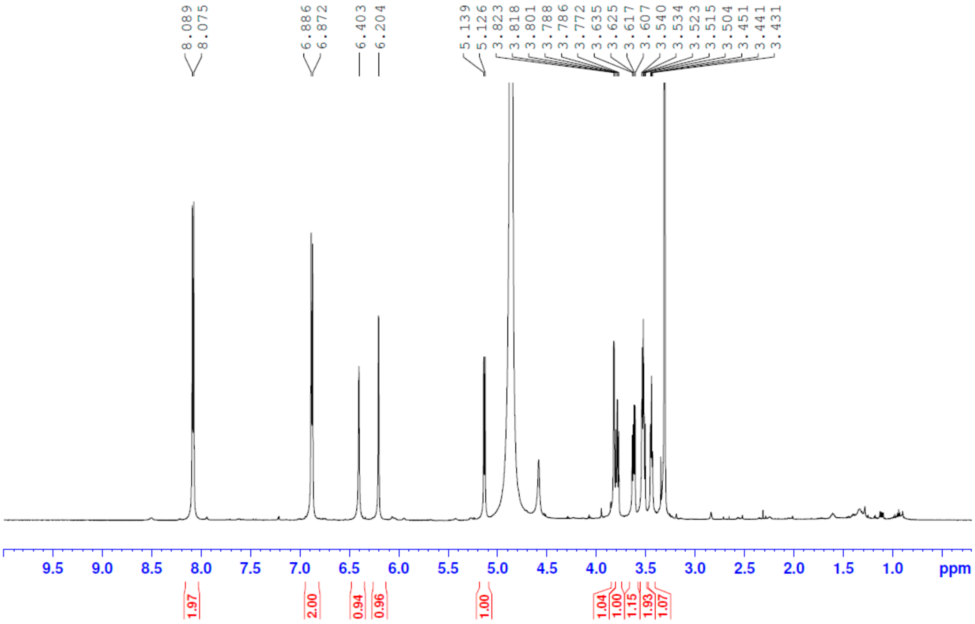

Figure S96. The ^{1}H-NMR (600MHz, CD_3OD) spectrum of compound 32

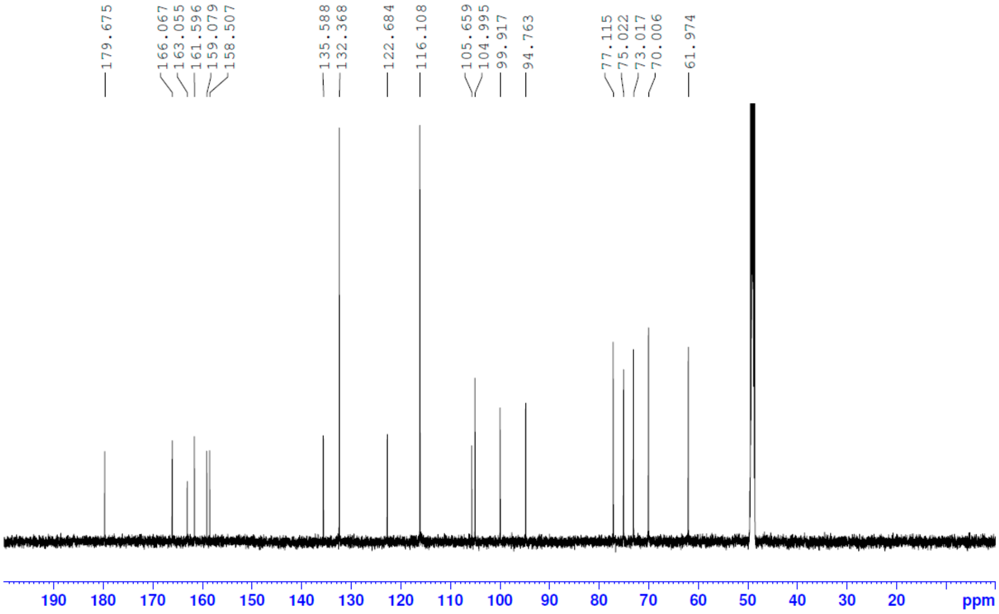

Figure S97. The ^{13}C-NMR (150MHz, CD_3OD) spectrum of compound 32

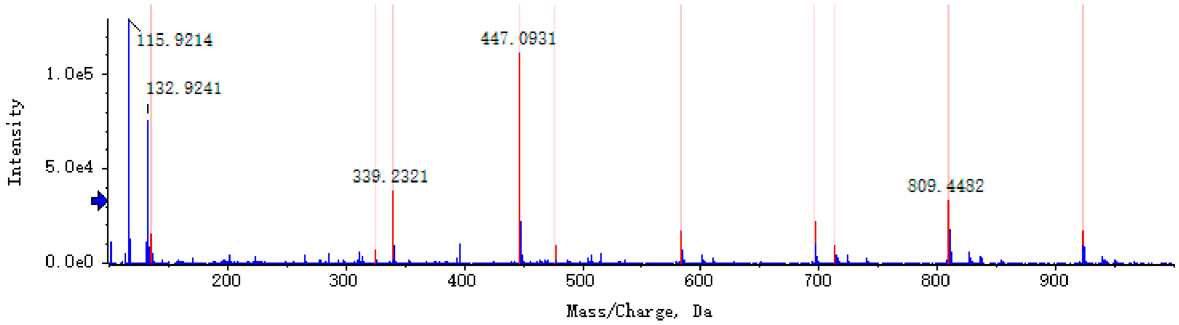

Figure S98. The ESI-MS spectrum of compound 33

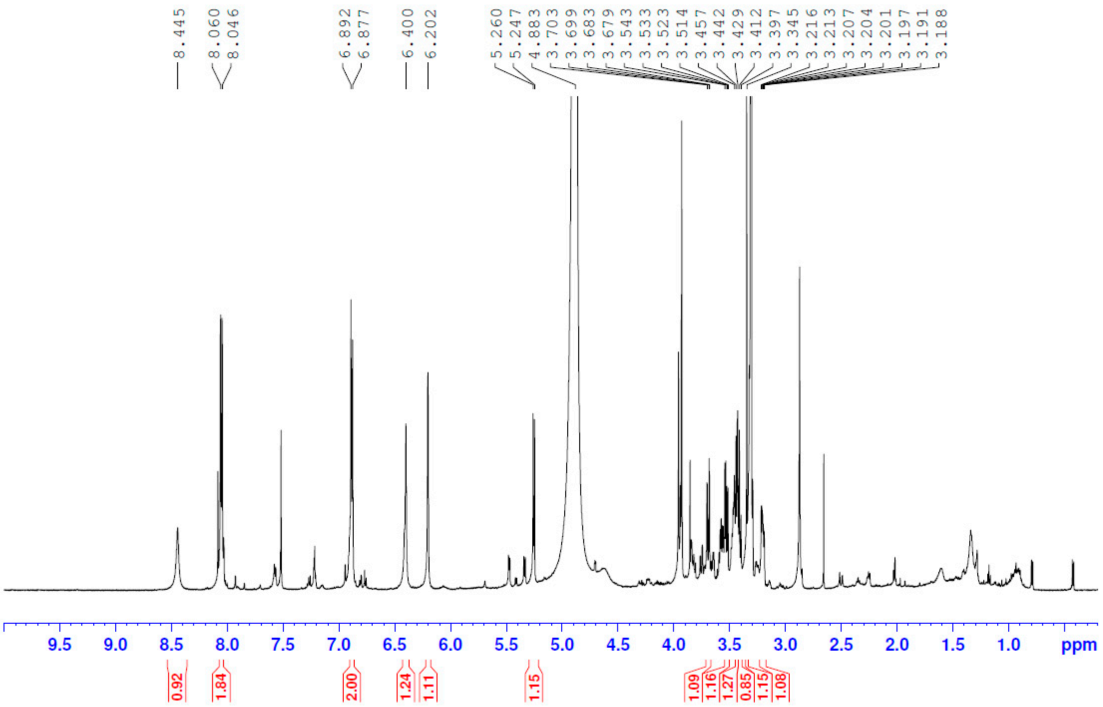

Figure S99. The ^{1}H-NMR (600MHz, CD_3OD) spectrum of compound 33

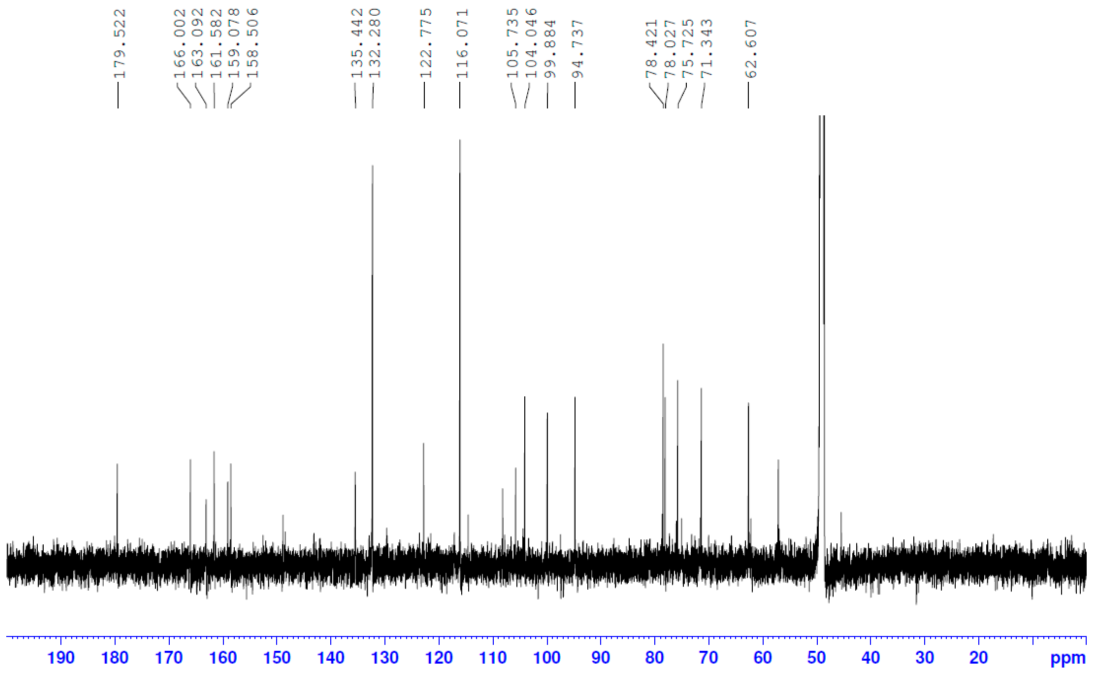

Figure S100. The ^{13}C-NMR (150MHz, CD_3OD) spectrum of compound 33

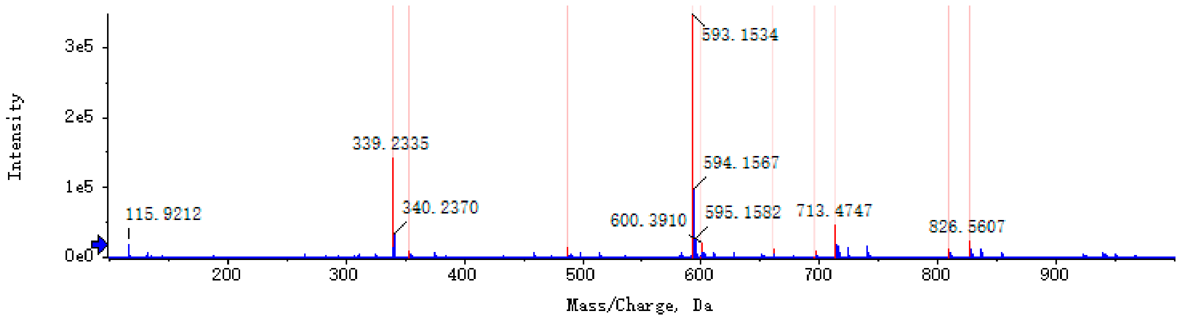

Figure S101. The ESI-MS spectrum of compound 34

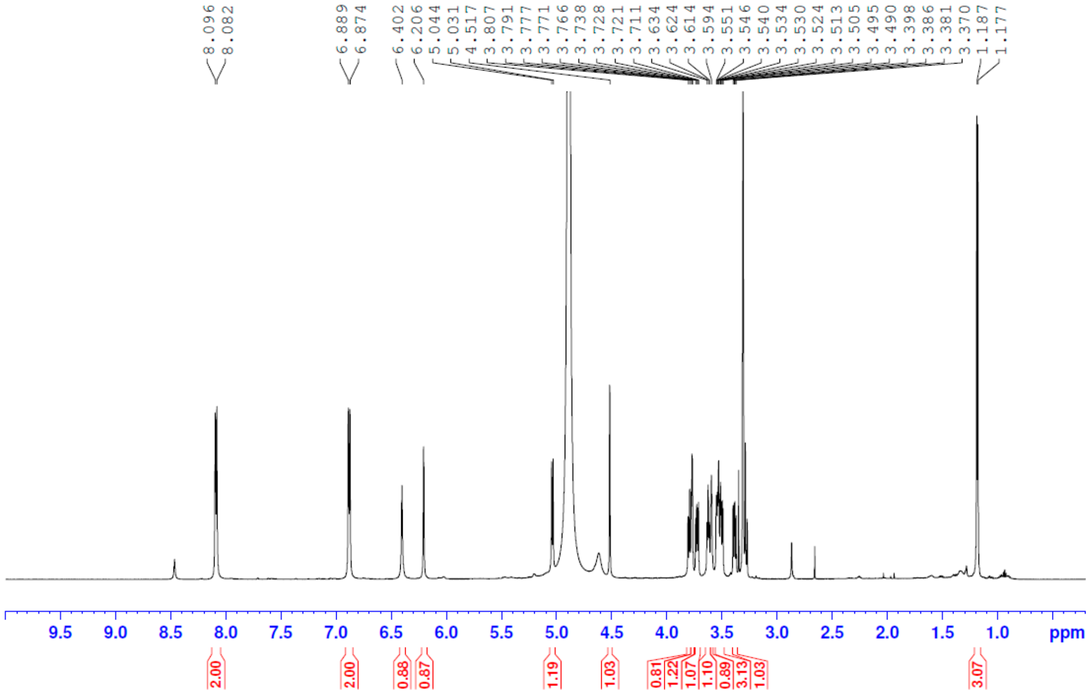

Figure S102. The ^{1}H-NMR (600MHz, CD_3OD) spectrum of compound 34

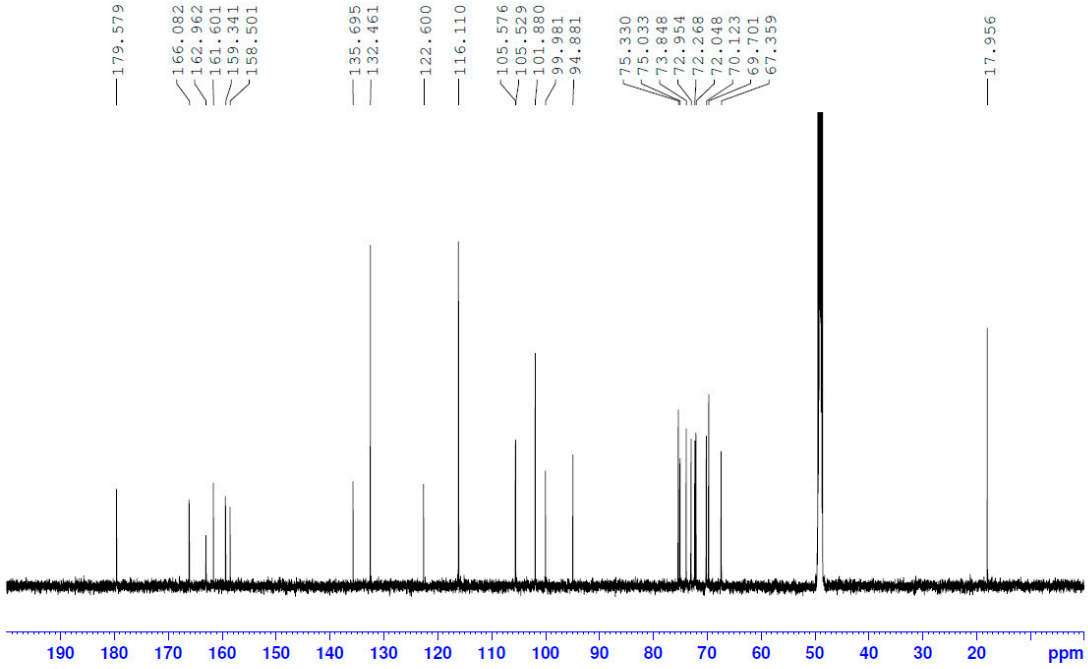

Figure S103. The ^{13}C-NMR (150MHz, CD_3OD) spectrum of compound 34

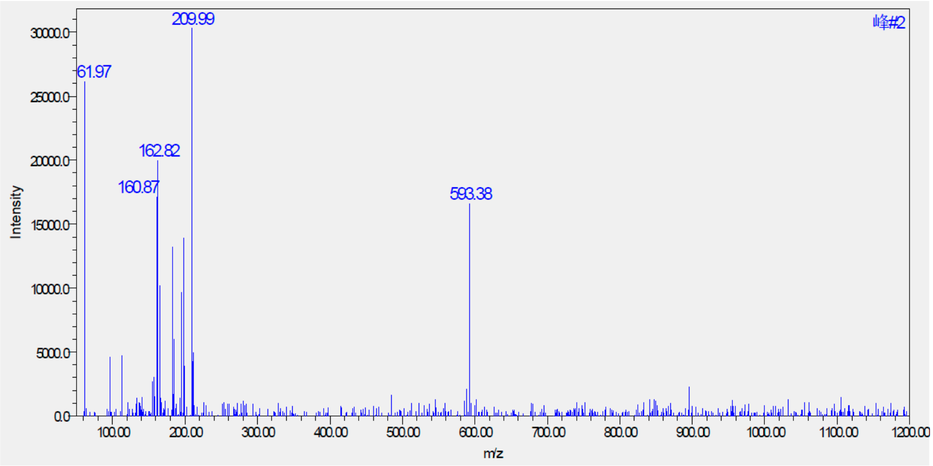

Figure S104. The ESI-MS spectrum of compound 35

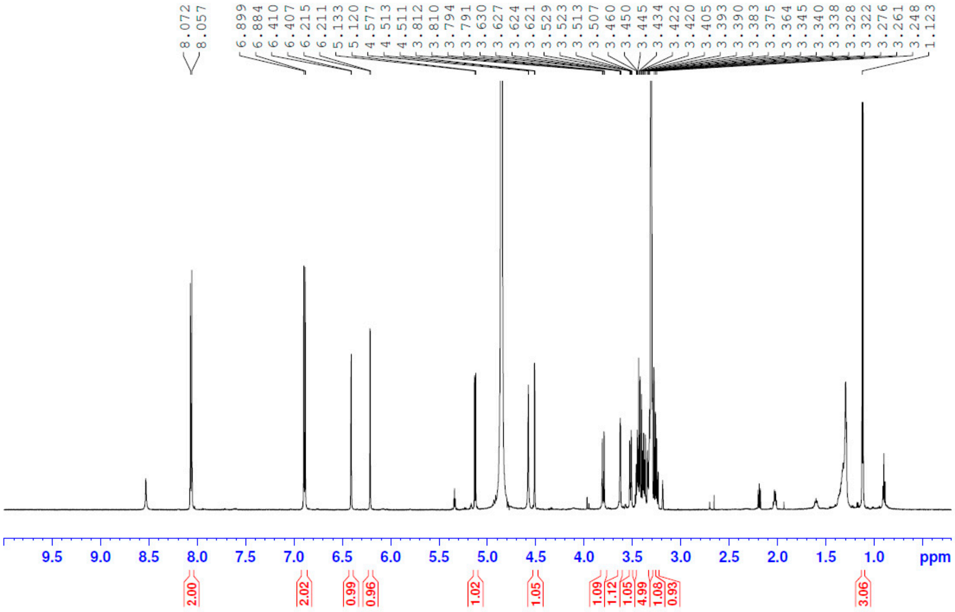

Figure S105. The ^{1}H-NMR (600MHz, CD_3OD) spectrum of compound 35

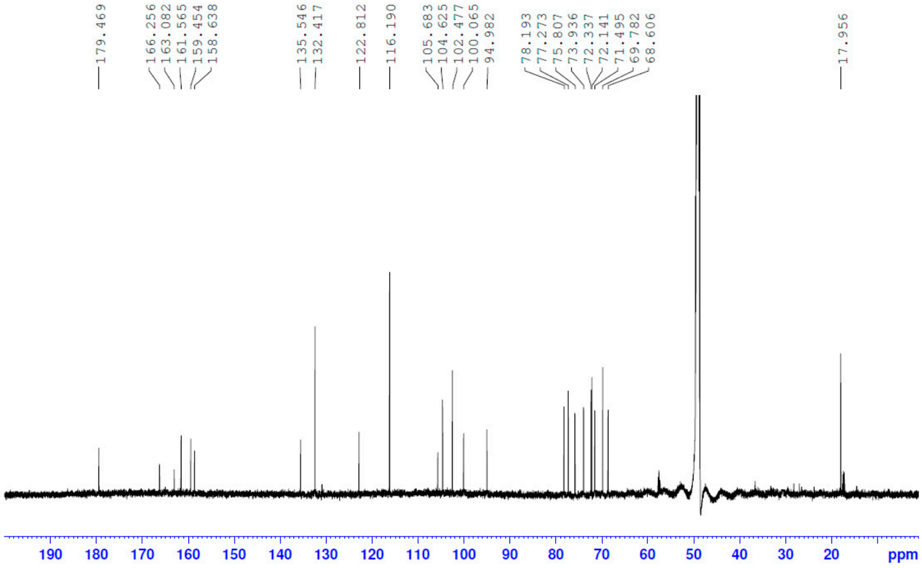

Figure S106. The ^{13}C-NMR (150MHz, CD_3OD) spectrum of compound 35

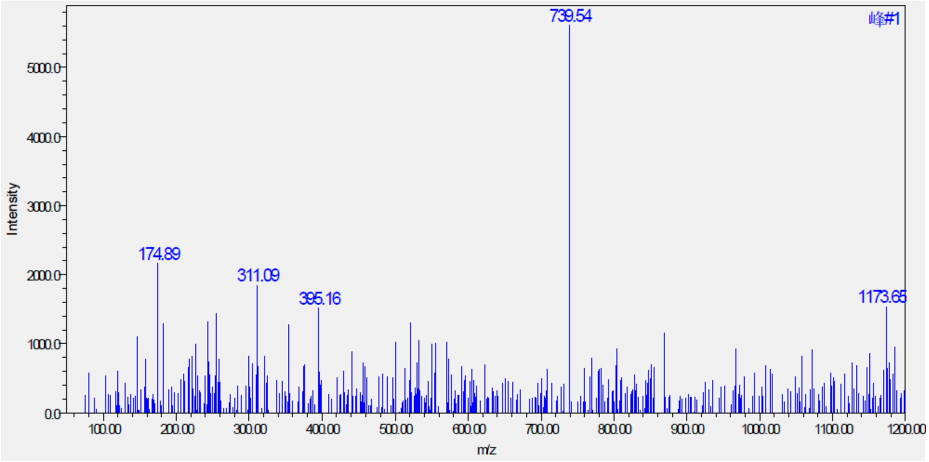

Figure S107. The ESI-MS spectrum of compound 36

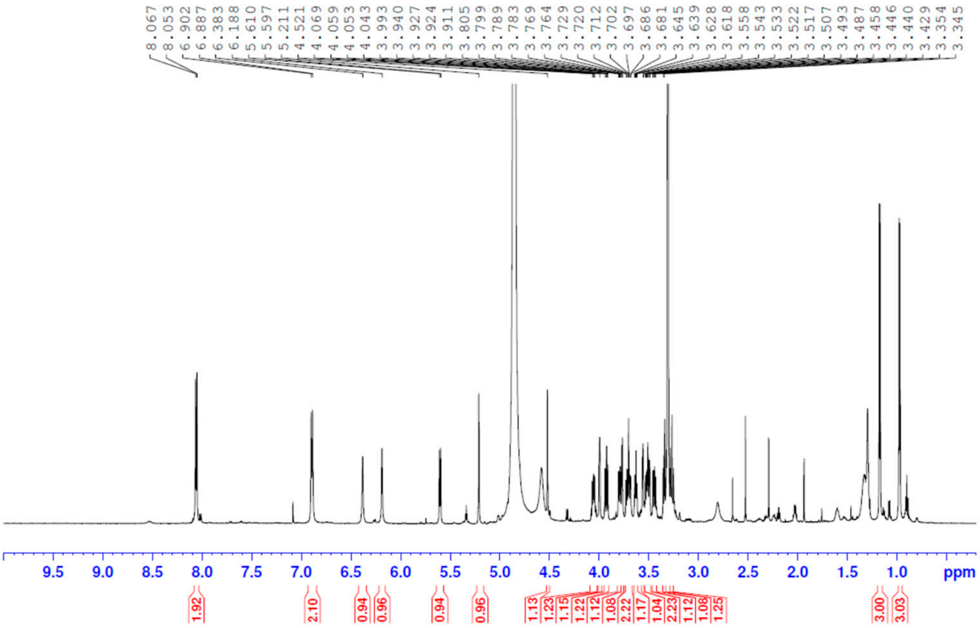

Figure S108. The ^{1}H-NMR (600MHz, CD_3OD) spectrum of compound 36

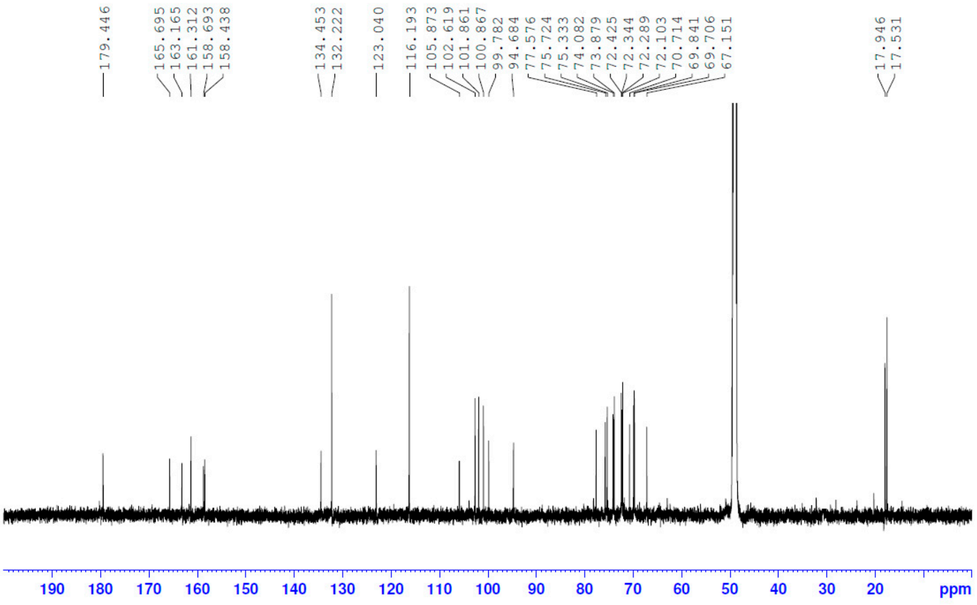

Figure S109. The ^{13}C-NMR (150MHz, CD_3OD) spectrum of compound 36

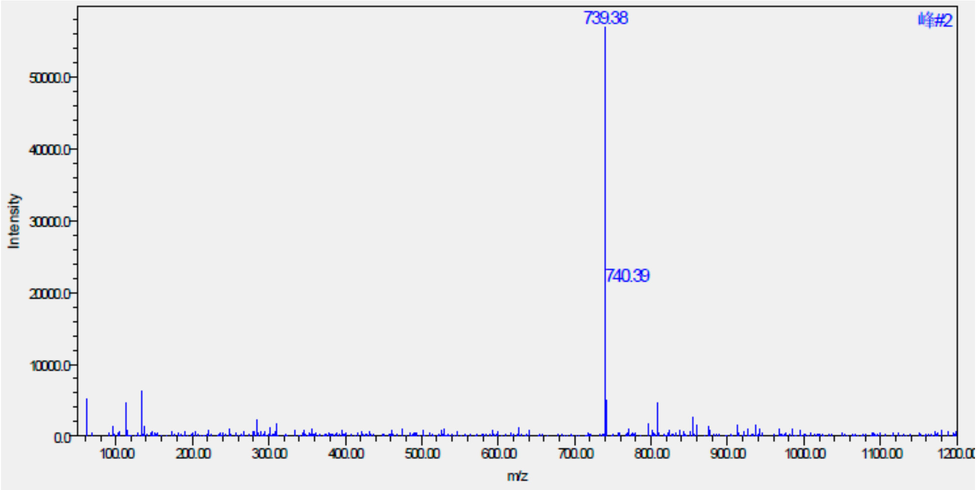

Figure S110. The ESI-MS spectrum of compound 37

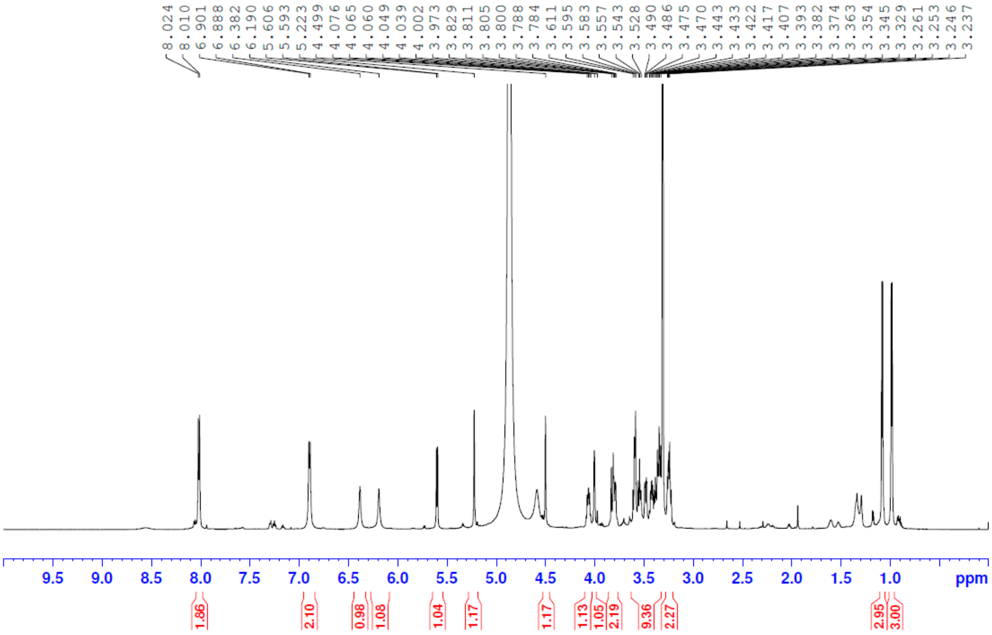

Figure S111. The ^{1}H-NMR (600MHz, CD_3OD) spectrum of compound 37

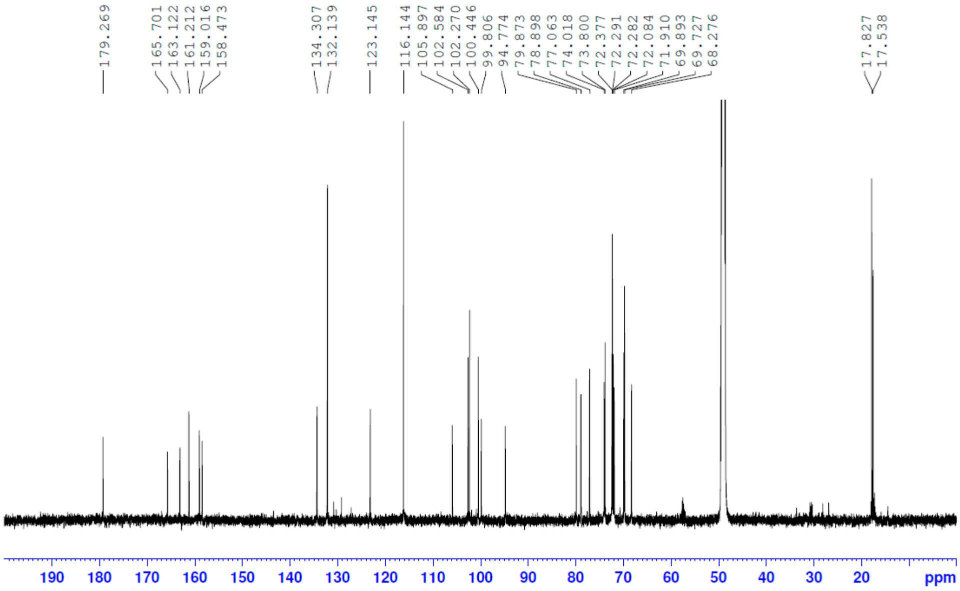

Figure S112 The ^{13}C-NMR (150MHz, CD_3OD) spectrum of compound 37

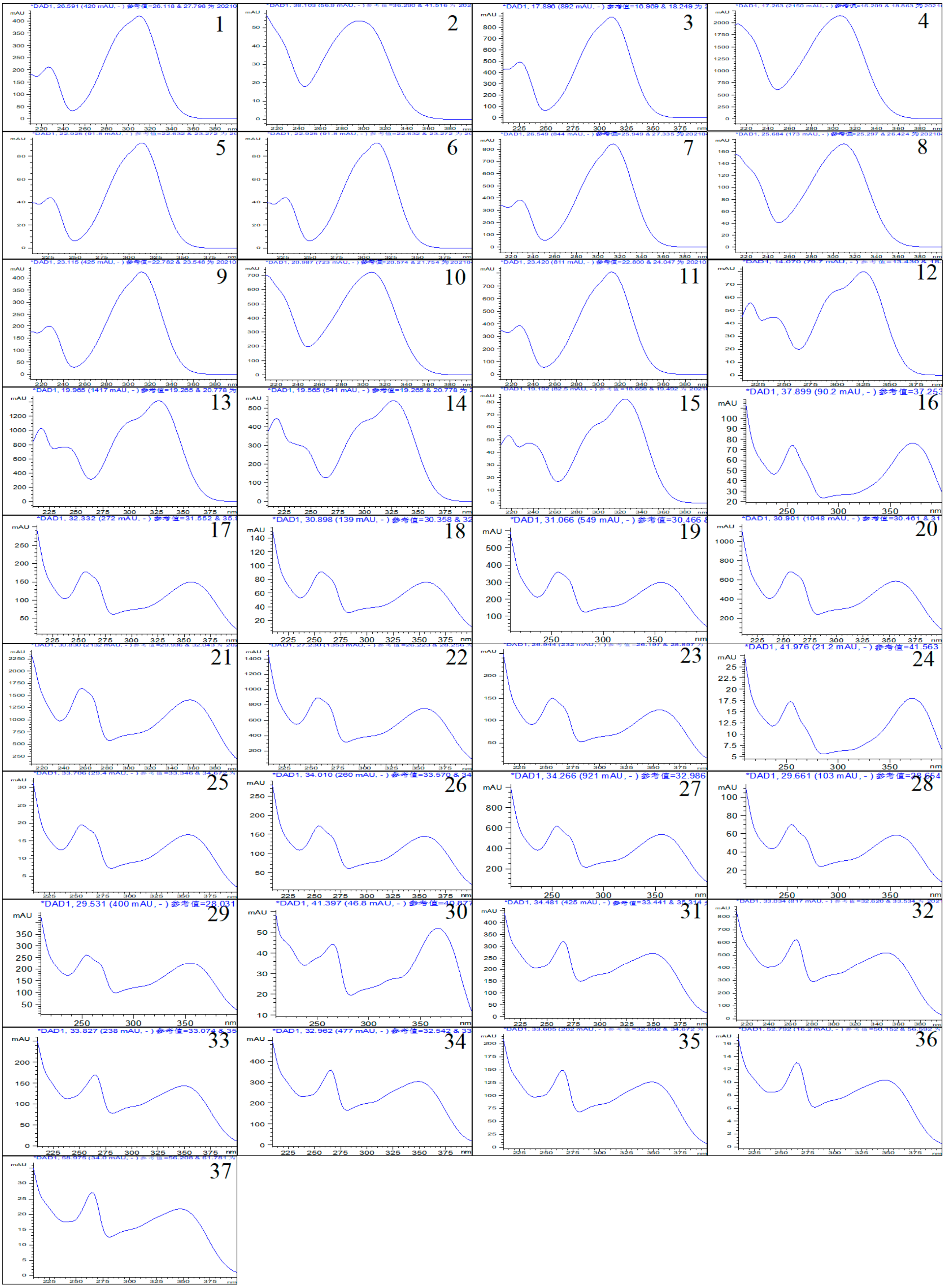

Figure S113 The UV spectrum of compound 1-37

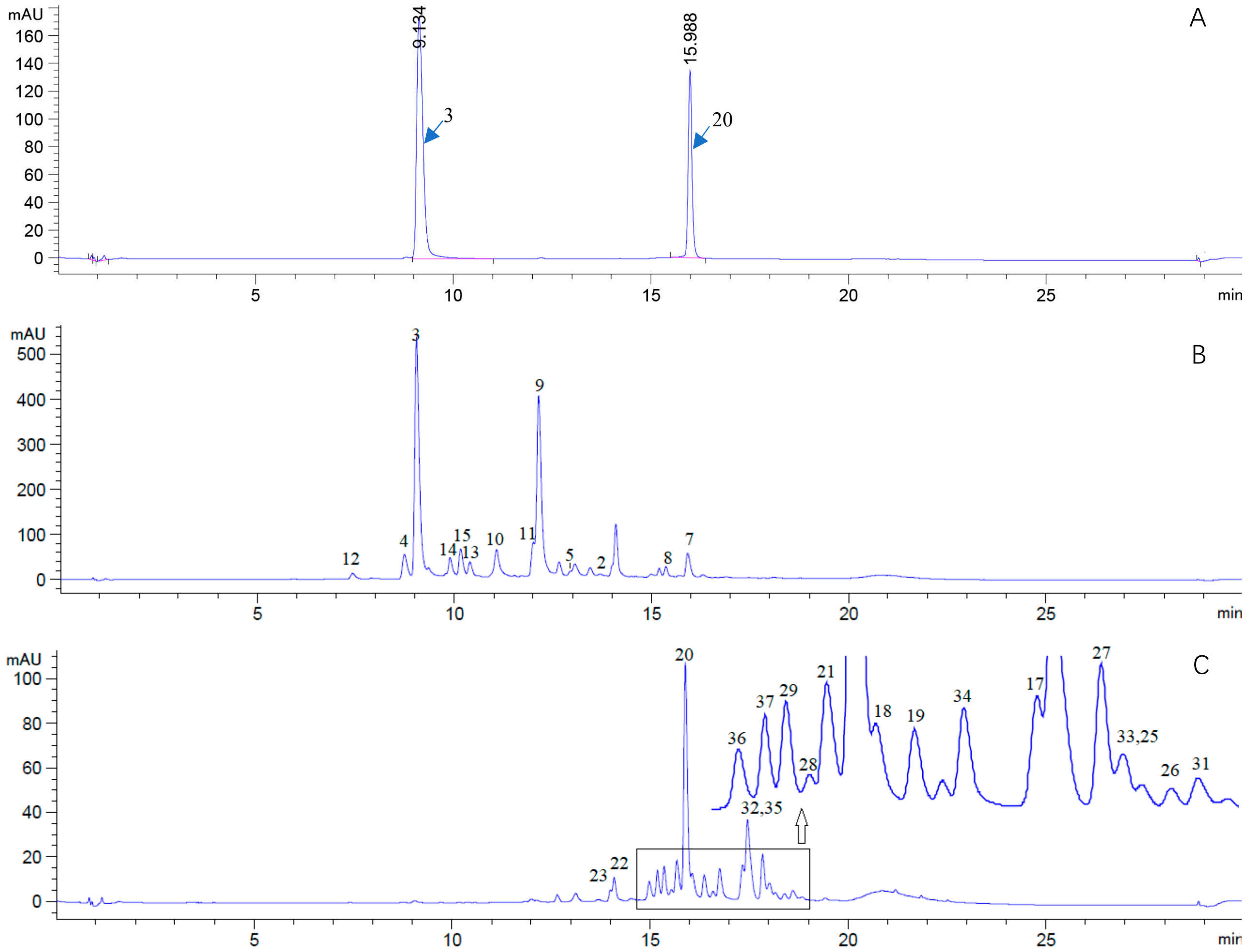

Figure S114 The UHPLC chromatogram of reference substance (A), HFPE(B) and HFPE(C). The analyses were performed by UHPLC system (Agilent, USA). The waters column (ACQUITY UPLC® HSS T3 1.7 μm, 2.1 × 100 mm) was used for separation. Mobile phases were water with 0.1% of formic acid (A) and acetonitrile with 0.1% of formic acid (B). Chromatographic separation was achieved using a gradient elution as follows: 0.01–2 min, 2%; 2–16 min, 2% to 20% B; 16–19 min, 20% to 23% B; 19–30 min, 23% to 100% B. A sample volume of 4 μL was injected and introduced to the column with 0.4 $mL \cdot min^{-1}$ of the solvent flow rate. The column temperature was set at 35℃.

Table S1 The phenylpropanoids compounds content in HFPE

Compound No.	Name	Retention time	Peak area	Content (mg/g)
12	neochlorogenic acid	7.420	118.59	2.62
4	3-O-*(Z)*-*p*-coumaroylquinic acid	8.736	441.09	9.75
3	3-*O-(E)*-*p*-coumaroylquinic acid	9.042	4482.65	99.13
14	chlorogenic acid	9.892	268.64	5.94
15	3-O-*(E)*-feruloylquinic acid	10.160	387.67	8.57
13	crypto-chlorogenic acid	10.396	217.97	4.82
10	4-O-*(Z)*-*p*-coumaroylquinic acid	11.069	618.46	13.68
11	5-O-*(E)*-*p*-coumaroylquinic acid	12.006	284.23	6.29
9	4-O-(*E*)-*p*-coumaroylquinic acid	12.138	3499.13	77.38
5	3-O-(*E*)-*p*-coumaroylquinic acid methyl ester	12.930	51.00	1.13
2	*(Z)*-*p*-coumaric acid	13.704	33.89	0.75
8	3-O-(*Z*)-*p*-coumaroylquinide	15.366	141.96	3.14
7	3-O-(*E*)-*p*-coumaroylquinide	15.920	418.99	9.27

Note: (*E*)-*p*-Coumaric acid (comp. 1) and 3-O-(*Z*)-*p*-coumaroyl quinic acid methyl ester (comp. 6) were not detected in UPLC, The content of each phenylpropanoids compound in HFPE was calculated by the external standard one point method using comp. 3 as reference substance.

Table S2 The flavonoids compounds content in HFFE

Compound No.	Compound name	Retention time	Peak area	Content (mg/g)
23	quercetin-3-O-*α*-L-rhamnopyranosyl-(1→2)-[*α*-L-rhamnopyranosyl-(1→6)-*β*-D-galactpyranoside]	14.003	20.98	3.41
22	quercetin-3-O-*α*-L-rhamnopyranosyl-(1→2)-[*α*-L-rhamnopyranosyl-(1→6)-*β*-D-glucopyranoside]	14.098	65.48	10.63
36	kaempferol-3-O-[*α*-L-rhamnopyranosyl-(1→6)]-[*α*-L-rhamnopyranosyl-(1→2)]-*β*-D-galactopyranoside	14.983	46.37	7.53
37	kaempferol-3-O-[*α*-L-rhamnopyranosyl-(1→6)]-[*α*-L-rhamnopyranosyl-(1→2)]-*β*-D-glucopyranoside	15.190	62.97	10.22
29	isorhamnetin-3-O-*α*-L-rhamnopyranosyl-(1→2)-[*α*-L-rhamnopyranosyl-(1→6)-*β*-D-glucopyranoside]	15.359	68.85	11.18
28	isorhamnetin-3-O-*α*-L-rhamnopyranosyl-(1→2)-[*α*-L-rhamnopyranosyl-(1→6)-*β*-D-galactopyranoside]	15.542	11.49	1.87
21	quercetin-3-O-*α*-L-rhamnopyranosyl-(1→6)-*β*-D-galactpyranoside	15.682	99.15	16.10
20	quercetin-3-O-rutinoside	15.893	659.98	107.14
18	quercetin-3-O-*β*-D-galactopyranoside	16.070	56.70	9.20
19	quercetin-3-O-*β*-D-glucopyranoside	16.369	59.59	9.67
34	kaempferol-3-O-*α*-L-rhamnopyranosyl-(1→6)-*β*-D-galactpyranoside	16.760	90.45	14.68
17	quercetin-3-O-*α*-L-arabinopyranoside	17.343	88.27	14.33
32, 35	kaempferol-3-O-*β*-D-galactopyranoside, kaempferol-3-O-*α*-L-rhamnopyranosyl-(1→6)-*β*-D-glucopyranoside	17.465	297.70	48.33
27	isorhamnetin-3-O-[*α*-L-rhamnopyranosyl-(1→6)-β-D-glucopyranoside	17.846	113.82	18.48
33, 25	kaempferol-3-O-*β*-D-glucopyranoside, isorhamnetin-3-O-*β*-D-galactopyranoside	18.016	34.52	5.60
26	isorhamnetin-3-O-*β*-D-glucopyranoside	18.397	14.09	2.29
31	kaempferol-3-O-*α*-L-arabinoside	18.613	28.72	4.66

Note: Quercetin (comp. 16), isorhamnetin (comp. 24) and kaempferol (comp. 30) were not detected in UPLC. The content of each flavonoid compound in HFFE was calculated by the external standard one point method using comp. 20 as reference substance. The two compounds of comp. 32 and comp. 35 were not separated by HPLC, their contents were calculated together. So as comp. 33 and comp. 25.

Determination of total phenolic

Both phenylpropanoids and flavonoids belong to total phenolic, so we measure total phenolic expressed phenylpropanoids and flavonoids. The total phenolic content of *H. citrina* was determined using Folin–Ciocalteu assay[1]with gallic acid as a standard. In brief, 1 mL sample methanolic solution was mixed with 0.5 mL Folin–Ciocalteu reagent (0.25 mol/L). After incubation for 3 min, 1.0 mL of Na_2CO_3 aqueous solution (15%, w/v) was added. After standing in dark for 30 min, the mixture was centrifuged at 3000 r/min for 5 min. The absorbance of the supernatant was measured at 760 nm (Aoxi UV1901PC, Shanghai, China). The total phenolic content of *H. citrina* was calculated based on a linear regression equation of Gallic acid (y = 10.226 x - 0.0773, x for gallic acid concentration, y for absorbance at 760 nm, *r*=0.9993), and was expressed as gallic acid equivalents 9.58 mg/g in *H. citrina.*

References

1. Li, X.; Hu, Q.; Jiang, S.; Li, F.; Lin, J.; Han, L.; Hong, Y.; Lu, W.; Gao, Y.; Chen, D., Flos Chrysanthemi Indici protects against hydroxyl-induced damages to DNA and MSCs via antioxidant mechanism. *J. Saudi. Chem. Soc.* **2015,** *19,* *454-460.*
